# Antitumor activity of the novel multi-kinase inhibitor EC-70124 in triple negative breast cancer

**DOI:** 10.18632/oncotarget.4736

**Published:** 2015-08-12

**Authors:** María Dolores Cuenca-López, Gemma Serrano-Heras, Juan Carlos Montero, Verónica Corrales-Sánchez, Mónica Gomez-Juarez, Maria José Gascón-Escribano, Jorge Carlos Morales, Veronique Voisin, Luz Elena Núñez, Francisco Morís, Gary D. Bader, Atanasio Pandiella, Alberto Ocaña

**Affiliations:** ^1^ Translational Research Unit, Albacete University Hospital, Albacete, Spain; ^2^ Cancer Research Center, CSIC-University of Salamanca, Spain; ^3^ The Donnelly Centre, University of Toronto, Canada; ^4^ EntreChem SL, Oviedo, Spain

**Keywords:** EC-70124, triple negative, breast cancer, kinase inhibitor

## Abstract

Disseminated triple negative breast cancer (TNBC) is an incurable disease with limited therapeutic options beyond chemotherapy. Therefore, identification of druggable vulnerabilities is an important aim. Protein kinases play a central role in cancer and particularly in TNBC. They are involved in many oncogenic functions including migration, proliferation, genetic stability or maintenance of stem-cell like properties. In this article we describe a novel multi-kinase inhibitor with antitumor activity in this cancer subtype. EC-70124 is a hybrid indolocarbazole analog obtained by combinatorial biosynthesis of Rebeccamycin and Staurosporine genes that showed antiproliferative effect and *in vivo* antitumoral activity. Biochemical experiments demonstrated the inhibition of the PI3K/mTOR and JAK/STAT pathways. EC-70124 mediated DNA damage leading to cell cycle arrest at the G2/M phase. Pathway analyses identified several deregulated functions including cell proliferation, migration, DNA damage, regulation of stem cell differentiation and reversion of the epithelial-mesenchymal transition (EMT) phenotype, among others. Combination studies showed a synergistic interaction of EC-70124 with docetaxel, and an enhanced activity *in vivo*. Furthermore, EC-70124 had a good pharmacokinetic profile. In conclusion these experiments demonstrate the antitumor activity of EC-70124 in TNBC paving the way for the future clinical development of this drug alone or in combination with chemotherapy.

## INTRODUCTION

Breast cancer is a heterogeneous disease as demonstrated at a genomic level with the description of different breast cancer subtypes with independent clinical outcome [1, 2, 3, 4]. Among them, triple negative breast cancer (TNBC) lacks the expression of hormone receptors and HER2 overexpression, representing 15% of all breast tumors [5]. It is associated with a specific pattern of tumor relapse and an increased sensitivity to chemotherapy [6, 7]. TNBC has been classified into several subtypes with different sensitivities to treatment [8]. Although the identification of subgroups in triple negative cancer represents a major advance, unfortunately the implementation of this classification for therapeutic purposes is unclear [9]. Indeed available therapeutic options for patients with TNBC are restricted to standard treatment with chemotherapy [9].

Membrane receptors and downstream kinase mediators are involved in the regulation of many cellular activities associated with human transformation [10, 11], including survival, response to DNA damage, migration, morphogenesis, cell adhesion, stem cell differentiation or proliferation, among others [12]. TNBC is a tumor subtype characterized by a high metastatic capacity, presence of stem cell precursors and a high proliferation rate [13, 14]. Similarly, alterations of the DNA repair machinery seem to be of great significance in this cancer [15, 16]. This breast cancer subgroup is enriched with genes associated to DNA damage response, when evaluated by gene expression analyses [8]. There is also an increased presence of somatic and acquired mutations in DNA repair genes, mainly BRCA1 and BRCA2, involved in the homologous recombination (HR) repair mechanism [15, 16]. Protein kinases also participate in the regulation and maintenance of genetic integrity [17].

Using human samples, our group and others evaluated the kinase profile of TNBCs, observing that a number of these proteins are constitutively activated [18, 19]. These included receptors and downstream mediators like ERK1/2, SRC, ERK5, STAT1 and STAT3 [19]. Of particular relevance was the activation of components of the PI3K/mTOR/AKT pathway such as pS6 or AKT. Interestingly, inhibition of the PI3K/mTOR route produced a proliferative arrest in cellular models and a growth reduction in tumors implanted in xenografted animals or generated using transgenic models [19]. Sequencing approaches have also confirmed the relevance of this route in TNBC suggesting that the PI3K/mTOR pathway is a significant druggable oncogenic alteration in this disease [20].

The translation of these findings to the clinical setting has been occasionally disappointing. Clinical studies evaluating drugs targeting individual proteins or pathways have shown negative results suggesting that therapeutic strategies should be designed to inhibit a number of key oncogenic nodes [21, 22], rather than individual kinases alone. This was the case for the negative results when targeting the Epidermal Growth Factor Receptor (EGFR) in TNBC [22]. Based on the global importance of protein kinases in TNBC [23], the development of novel polypharmacology inhibitors that could hit relevant signalling pathways simultaneously, is a desirable goal.

EC-70124 is a hybrid indolocarbazole analog obtained by combinatorial biosynthesis of Rebeccamycin and Staurosporine gene pathways and produced by fermentation [24]. EC-70124 shows antitumor activity in a wide range of solid tumors through the inhibition of key signaling nodes at a nanomolar range [24]. EC-70124 has completed its safety evaluation in preclinical models and is starting its clinical development [24].

In the present study we evaluated the anti-tumor properties of this novel multi-kinase inhibitor in TNBC. We observed that EC-70124 is active against a panel of TNBC cells and in xenografted mice. EC-70124 inhibited relevant pathways in TNBC including the PI3K/mTOR pathway or STAT1/3. In addition, it modified several cellular functions including DNA damage, migration and stem cell differentiation, among others. Finally EC-70124 synergized *in vivo* and *in vitro* with taxanes.

## RESULTS

### Antitumor effect of EC-70124 in TNBC

**Figure 1:**
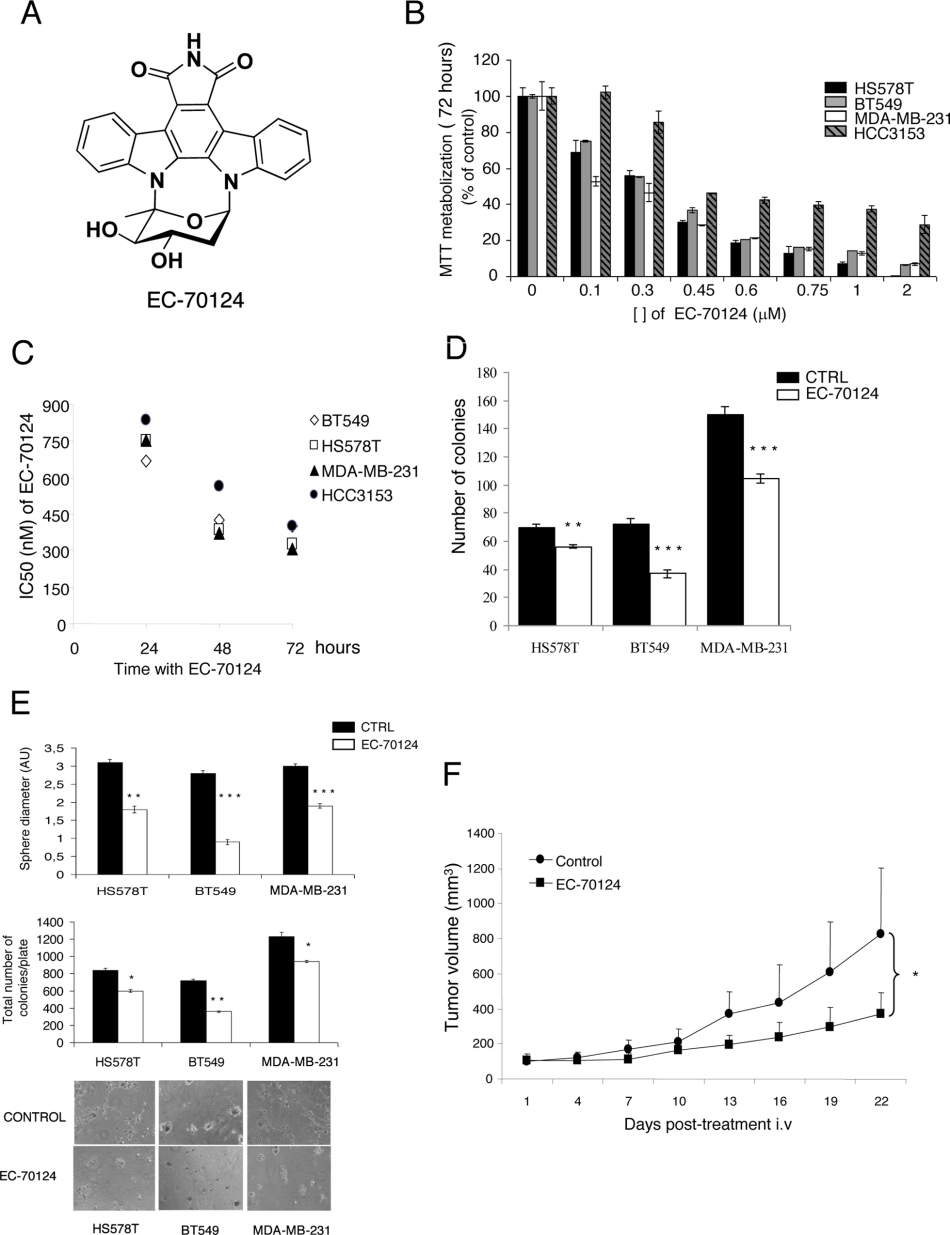
Chemical structure of EC-70124 and anti-tumor action *in vitro* and *in vivo* **A.** Representation of the chemical structure of EC-70124. **B, C.** Effect of EC-70124 on cell proliferation in HS578T, BT549, MDA-MB-231 and HCC3153. (B) Cells were cultured in DMEM and RPMI + 10% FBS in the presence of EC-70124 at a concentration range of 0.1–2 μM, in a dose-dependent manner, for 72 hours (C) The inhibitory effect, as the IC50, was calculated after 24, 48, 72 hours of drug incubation using MTT assays. Metabolization of MTT in viable cells was determined by spectrophotometry. The mean absorbance values of untreated cell lines (controls) were taken as 100%. **D.** Action of EC-70124 on colony formation in HS578T, BT549, MDA-MB-231. Cells were treated with EC-70124 (300 nM) for 24 h. Data are represented as the mean ± s.d. of triplicate experiments. Student's test was used to calculate statistical significance: **p < 0.005, ***p < 0.001. **E.** Effect of EC-70124 on cell growth in semi-solid medium in HS578T, BT549 and MDA-MB-231. Cells were plated in 48 multiwell plates and grown in medium containing matrigel for 7 days in the presence of EC-70124 (500 nM). All images were taken at ×20 magnification. The quantitation of sphere diameter was performed manually by tracing a straight line across the sphere diameter of untreated cells (controls) and scoring its value as arbitrary length units. Total number of colonies per plate was manually counted. * Indicates a *P* < 0.05, ** indicates *p* < 0.005, and *** indicates *p* < 0.001 with respect to each control. **F.** Growth inhibition of human breast tumor xenografts in female BALB/c nude mice by treatment with EC-70124. Animals were randomly assigned to two groups and received either intravenous EC-70124 (18 mg/kg/3 days/i.v.) or vehicle. The *p*-value (* < 0.05) was calculated on day 22 comparing the tumor volume of both groups using the Student's *t*-test. Bars indicate s.d.

Figure 1A represents the chemical structure of EC-70124. To explore the antitumor effect of this compound on TNBC, we used a panel of representative cell lines including HS578T, BT549, MDA-MB-231 and HCC3153. In these cells, treatment with EC-70124 inhibited MTT metabolization in a dose and time dependent manner (Figure 1B, 1C). As shown in Figure 1C the IC50 values for different time points were in the nanomolar range.

The clonogenic cell survival assay determines the ability of a cell to proliferate indefinitely, thereby retaining its reproductive ability to form a large colony or a clone. Treatment with EC-70124 strongly decreased the number of colonies formed at 10 days in HS578T, BT549 and MDA-MB-231 (Figure 1D).

The effect of EC-70124 on three-dimensional growth were also assessed. For this purpose, we cultured HS578T, BT549 and MDA-MB-231 in matrigel, a semisolid media where cells grow forming spherical structures. Treatment with the drug strongly decreased the diameter of these spheres (Figure 1E) (control vs treatment, mean diameter and SD = 2.1 +/−0.15 vs 0.9 +/−0.14 and 2.6 +/−0.1 vs 1 +/−0.14, and 3 +/−0.17 vs 1 +/−0.18 for HS578T, BT549 and MDA-MB-231, respectively). Similarly, a reduction in the total number of colonies per plate was observed. This data demonstrates the effect of EC-70124 on the inhibition of cell growth in 3D cultures.

Finally, to investigate the *in vivo* effect of EC-70124 on the growth of TNBC, we implanted MDA-MB-231 into the mammary fat pad of mice. Treatment with EC-70124 (18 mg/kg i.v. every 3 days) showed anti-tumor effect compared to control (Figure 1F). The mean tumor volume (mm^3^) measured every 3 days was significantly higher in the control versus the treated group (day 22; control vs treated, mean volume 826 vs 369 mm^3^, SD +/−376 and +/−121.5, respectively; *p* = 0.036). We also measured the drug effect on body weight and other toxicity parameters as mice behavior or skin modifications. We did not find differences between control vs treated animals in any of these parameters (Supplementary Figure S1A).

### Effect of EC-70124 on the TNBC kinase profile

**Figure 2:**
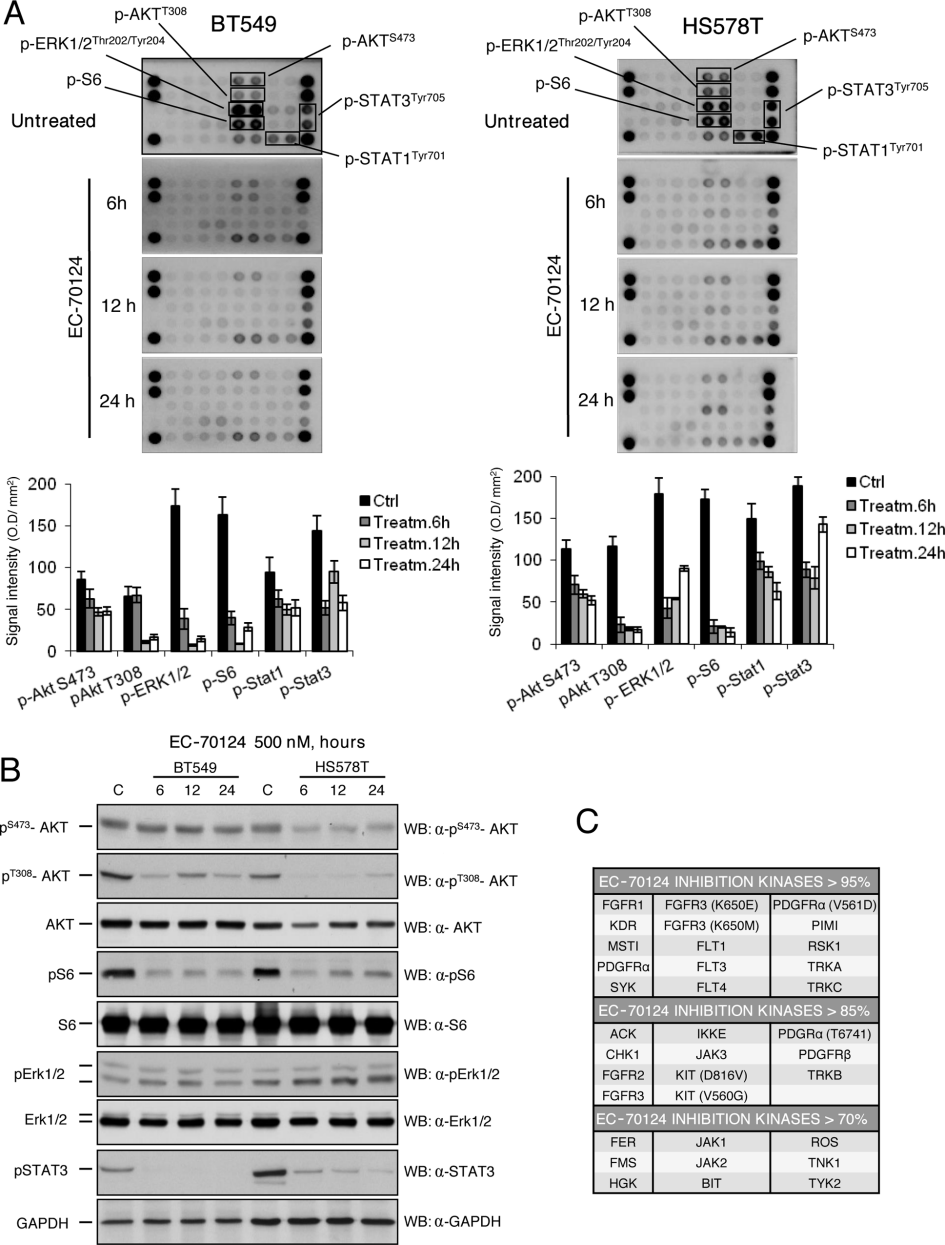
Effect of EC-70124 on signaling routes and kinase inhibitory profile **A.** Expression of activated signaling proteins in BT549 and HS578T; and effect of EC-70124 on their phosphorylated status. Cell lysates were examined for the phosphorylation of a panel of eleven intracellular kinases using an antibody array described in “Material and methods”. Bar graphs represent the expression of pS473-AKT, pT308-AKT, pS6, pErk1/2, pSTAT1 and pSTAT3. **B.** Expression of pAKT, AKT, pERK1/2, Erk1/2, pS6, S6 and pSTAT3 in BT549 and HS578T; and effect of EC-70124 at 500 nM. Cell lysates were prepared and the activation state of the mentioned proteins using Western blot analysis. GAPDH was used as a loading control. Antibodies used are described in “Material and methods”. **C.** EC-70124 kinase activity was profiled at 100 nM on a 169 kinase panel using a mobility shift assay at 1 mM ATP concentration for all kinases. A detailed description is given in “Material and methods”.

To evaluate the effect of EC-70124 on several protein kinases in HS578T and BT549, we used two phospho-kinase array kits. EC-70124 was able to inhibit downstream components of the PI3K/AKT pathway including AKT (phosphorylated at T308 and S473) and pS6 (Figure 2A). We confirmed these results by Western-blot analyses, except for the inhibition of pAKT-S473 in BT549 (Figure 2B). Similarly, p-Stat3 and p-Stat1 were also inhibited in HS578T and BT549 (Figure 2A, 2B). Of note, we were unable to confirm by Western-blot the inhibition of pErk1/2 observed in the kinase array.

To have a global view of the kinase activity of EC-70124, a 169 kinase panel using a mobility shift assay at 1mM ATP concentration for all kinases (well above their Km value and similar to the intracellular ATP concentration) was profiled by Carna Biosciences (Kobe, Japan). Figure 2C shows a list of kinases with various degrees of inhibition at 100 nM EC-70124. Some of the RTKs for which EC-70124 showed inhibitory effect in the kinase assay were not affected in HS578T and BT549. This effect although surprising could be explained by the limited phosphorylated expression of RTK in cell lines in basal conditions compared with other downstream mediators (Supplementary Figure S1B).

### EC-70124 induces G2/M arrest

**Figure 3:**
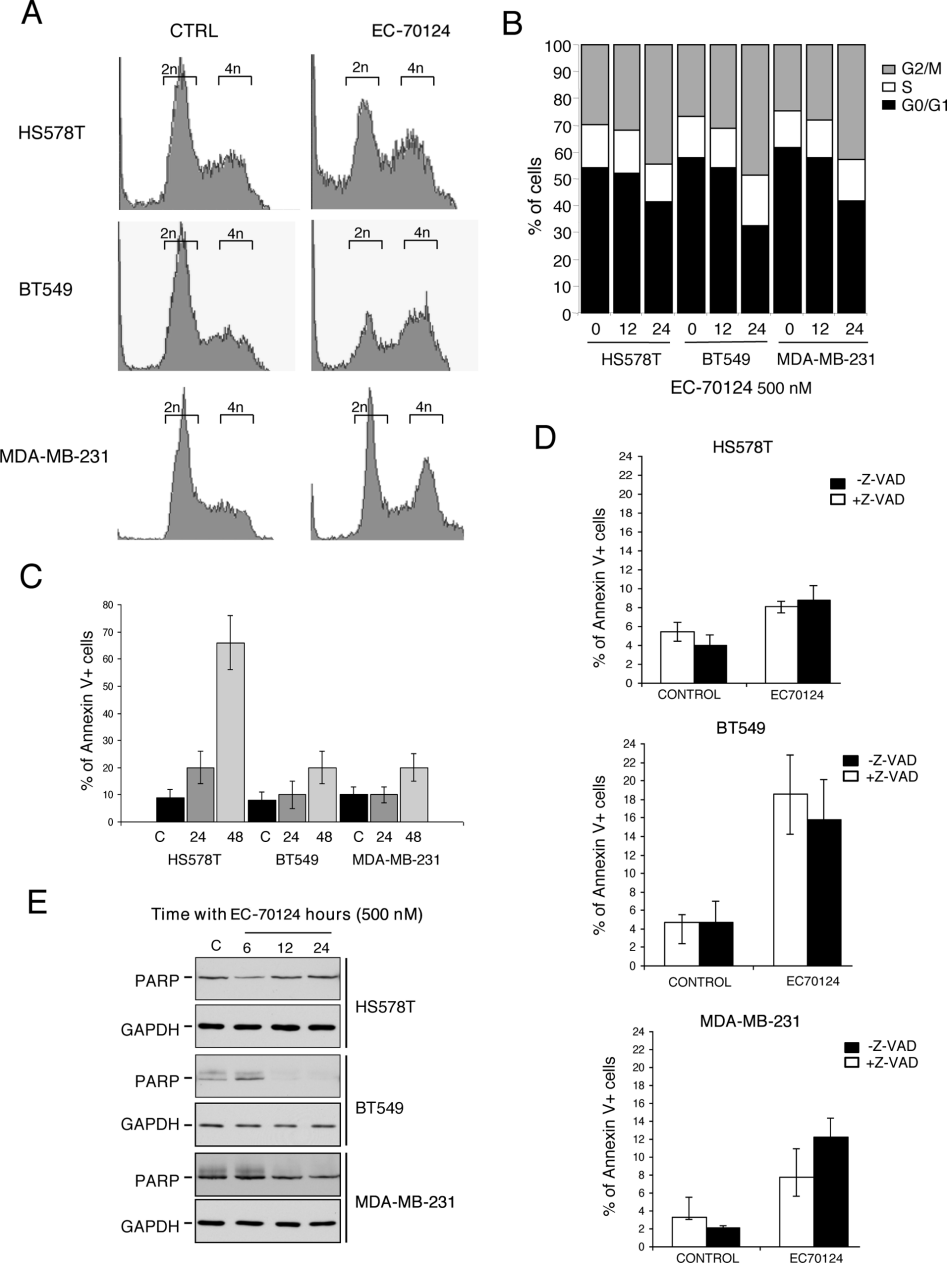
Cell cycle analyses and effect on apoptosis of EC-70124 **A, B.** Effect of EC-70124 on cell cycle in HS578T, MDA-MB-231 and BT549 (A) and graphical representation (B) Cells were cultured in DMEM 10% FBS and treated with 500 nM EC-70124. Cell cycle progression was examined after 12 and 24 hours of treatment by flow cytometry using propidium iodide DNA staining. The histogram (B) shows the mean percentage of cells in different phases of cell cycle after 12 and 24 hours of treatment. **C.** Apoptotic effect of EC-70124 in HS578T, MDA-MB-231 and BT549. Cells were stained with Annexin V after 24 and 48 hours of treatment. The histogram represents the mean percentage of cells positive or negative for Annexin V staining. **D.** Induction of apoptosis with EC-70124 (500 nM) in the presence or absence of the pancaspase inhibitor Z-VAD-FMK. MDA-MB-231, HS578T and BT549 were cultured in DMEM 10% FBS and treated with 500 nM EC-70124 for 24 hours with and without the inhibitor. The histogram represents the mean percentage of cells positive or negative to Annexin V staining. **E.** Evaluation of PARP cleavage by Western blotting in HS578T, BT549 and MDA-MB-231 after treatment with EC-70124 at 6, 12 and 24 hours. GADPH was used as a loading control. The antibody used is described in “Material and methods”.

To gain insights into the mechanism of the antiproliferative action of EC-70124 we explored the effect of the drug on cell cycle and apoptosis. Propidium iodide staining of HS578T, BT549 and MDA-MB-231 showed that EC-70124 induced accumulation of cells in G2/M phase (Figure 3A, 3B), an effect which was more pronounced at 24 h (15%, 22% and 18% increase for HS578T, BT549 and MDA-MB-231, respectively). Next, we used Annexin V staining to explore the effect of EC-70124 on apoptosis, observing an increase at 48 hours, more evident in HS578T (Figure 3C).

Induction of apoptosis can be caspase-dependent or caspase-independent. To explore the mechanism of cell death induced by the drug we treated HS578T, BT549 and MDA-MB-231 with the pancaspase inhibitor Z-VAD-FMK and evaluated Annexin V staining at 48 hours. We found no modifications in apoptosis with and without the presence of the pancaspase inhibitor in HS578T and BT549, and a small decrease in apoptosis in MDA-MB-231, showing that the proapoptotic effect is mainly independent of caspases (Figure 3D). In addition, treatment with EC-70124 did not produce cleavage of PARP at different time points (Figure 3E).

### EC-70124 modifies different pathways

**Figure 4:**
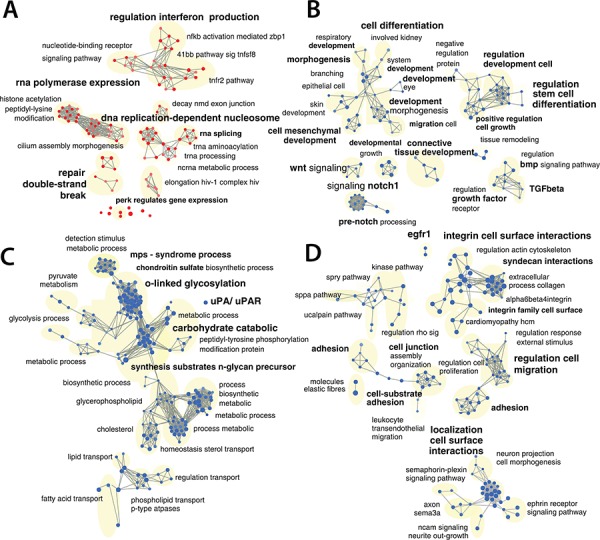
Gene-set enrichment analyses of EC-70124 **A, B, C, D.** Network representation of enriched gene-sets (*p*-value <= 0.025 and a FDR < 0.25), deregulated by EC-70124 at 500 nM including gene-sets associated with DNA damage, translation and regulation of gene expression (A), cell mesenchymal development and regulation morphogenesis of stem cell differentiation (B), EMT phenotype (C), adhesion, migration and cell proliferation (D) The red nodes (circles) are gene sets (pathways) significantly enriched in genes up-regulated in the EC-70124 treated samples, and the blue nodes are significantly enriched in genes down-regulated. Gray edges are lines that connect 2 gene sets if they contain overlapping genes. Gene sets connected by multiple edges form a cluster also called a module because they represent a similar biological process.

In parallel to the biochemical analyses, we also performed mRNA expression profiling studies. For this purpose, we treated MDA-MB-231 with EC-70124 using the IC50, and extracted mRNA at 24 hours. A gene set enrichment analysis (GSEA) based on a comprehensive pathway database (4319 gene sets) identified 938 gene sets affected by the drug treatment (*p*-value <= 0.025 and FDR <= 0.25), representing diverse biological functions (Supplementary Table S1 – GSEA_table). A network was created and grouped these gene-sets in 166 functional modules. Based on the gene overlap between these modules (map layout) as well as the functional annotations, it was possible to organize and summarize the results in 4 main categories (Figure 4A, 4B, 4C and 4D): gene sets associated with DNA damage response, translation and regulation of gene expression were up-regulated as shown in Figure 4A; gene sets down-regulated in the treated samples included functions or pathways such as: cell mesenchymal development/morphogenesis and regulation of stem cell differentiation (Figure 4B); EMT related glycan metabolism (Figure 4C); cell adhesion, extracellular matrix, migration and proliferation (Figure 4D). A list of selected modified genes included in these gene sets is shown in Supplementary Table S2.

**Figure 5:**
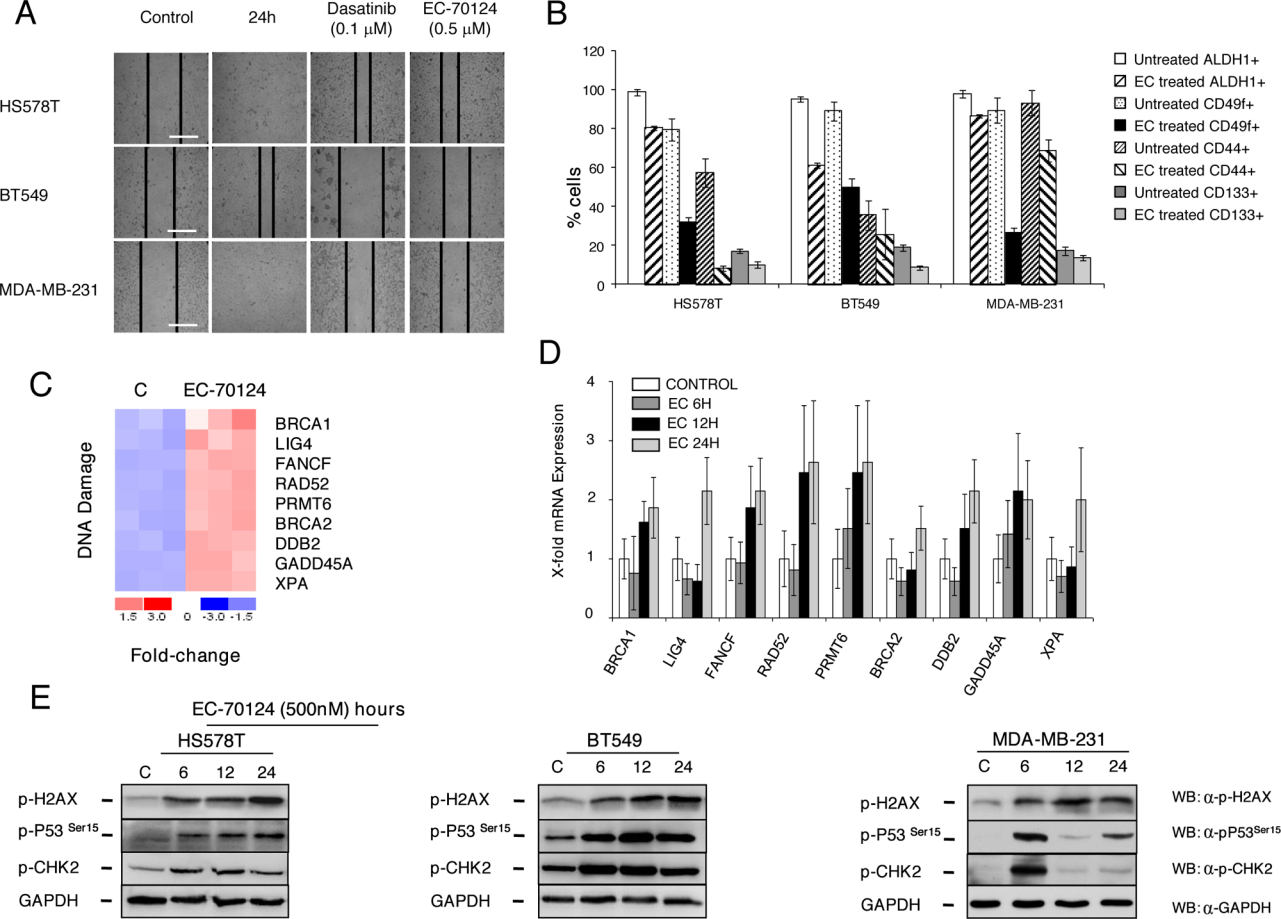
Effect of EC-70124 of migration, expression of stem-cell markers, and induction of DNA damage and activation of gene response **A.** Effect of EC-70124 on cell migration. HS578, BT549T and MDA-MB-231 were treated with EC-70124 at 500 nM and photographs were taken at 24 hours. Dasatinib at 100 nM were used as a control for inhibition of migration. **B.** Expression of CD44, CD49f, CD133 and ALDH1 after treatment with EC-70124 measured by flow cytometry in MDA-MB-231, HS578T and BT549. The histogram represents the percentage of positive cells. **C.** Fold change of genes related to DNA damage in treated versus untreated MDA-MB-231 cells. Red and blue colors represent the level of gene expression at greater than mean, and lower than mean, respectively. The numeric scale indicates the fold change of expression of each gene in each sample with respect to its mean expression value across all samples. Gene expression analyses is described in “Material and methods”. **D.** Representation of the transcription level of selected genes related to DNA damage in untreated and treated MDA-MB-231 cells (500 nM), at different time points (6, 12, 24 hours) measured by qRT-PCR. **E.** Effect of EC-70124 on p-H2AX, p-P53 and p-Chk2 in HS578T, BT549 and MDA-MB-231. Cells were treated with EC-70124 (500 nM) and mentioned proteins were evaluated by Western-blot. Antibodies used are described in “Material and methods”.

Next, we explored whether some of the observed functions could be confirmed with cellular and biochemical experiments. As shown in Figure 5A treatment with EC-70124 reduced cell migration in MDA-MB-231, BT549 and HS578T. Similarly we confirmed the reduced expression of CD44, ALDH1, CD49f and CD133 after treatment with EC-70124, all representative stem cell markers associated with the “regulation of stem cell differentiation” pathway (Figure 5B).

### EC-70124 induces DNA damage

Among causes that produce an increase of DNA repair genes and an arrest at G2/M phase is the presence of lesions in the DNA and the subsequent intent to repair and maintain its integrity [17]. In the gene enrichment analyses we observed that EC-70124 induced genes related to DNA repair mechanism at 24 hours that were also confirmed by qPCR (PRMT6, RAD52, GADD45A, XPA, LIG4, FANCF, BRCA1, BRCA2, DDB2) (Figure 5C, 5D). Therefore, to investigate if EC-70124 induced DNA damage, we analyzed the levels of phosphorylated γH2AX. It is known that this protein is required for checkpoint-mediated cell cycle arrest and DNA repair following double-stranded DNA breaks [17, 25]. Treatment with EC-70124 in HS578T, BT549 and MDA-MB-231 showed an increase in the phosphorylated levels of γH2AX at early time points (Figure 5E). In response to DNA DSBs, ATM phosphorylates multiple substrates including, Chk2, p53, and γH2AX [17, 26]. We observed that EC-70124 induced the phosphorylation of p53 and Chk2 (Figure 5E) confirming the induction of DNA damage.

### EC-70124 synergizes with standard of care chemotherapies

**Figure 6:**
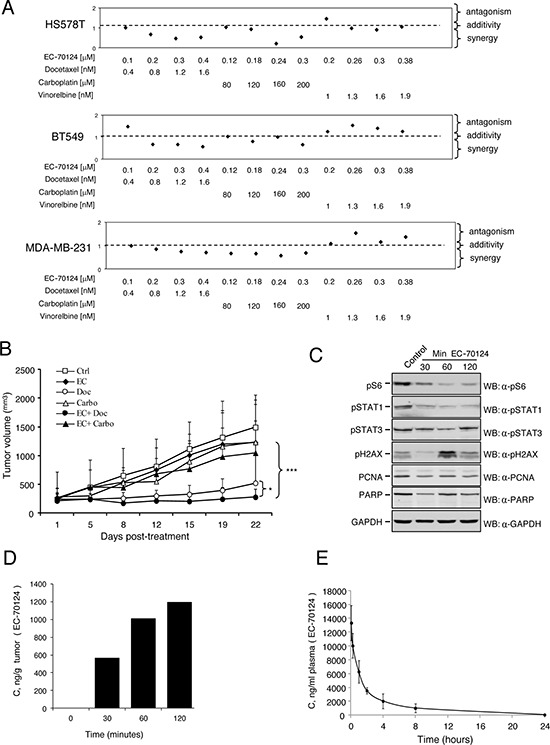
Combinational studies *in vitro* and *in vivo*; and pharmacokinetic and pharmacodynamic evaluation **A.** Effect of the combination of EC-70124 and standard of care drugs used in TNBC on cell proliferation. HS578T, BT549 and MDA-MB-231 cells were treated at the indicated concentrations of EC-70124, vinorelbine, carboplatin and docetaxel, alone and in combination, and their MTTs values were calculated. Combination indexes for the different drug combinations were obtained using CalcuSyn program and plotted. **B.** *In vivo* antitumor action of EC-70124 with docetaxel and carboplatin Nude mice were injected with MDA-MB-231. When tumors reached 200 mm^3^ animals were treated with drugs alone or in combination, at the mentioned doses described in “Material and methods”. Data represent the mean +/− s.d. *P < 0.05, ***P < 0.001. **C.** Effect of EC-70124 on expression of p-S6, p-STAT1, p-STAT3, p-H2AX, PCNA and PARP in mice tumors. Animals were treated for 30, 60 and 120 minutes with EC-70124 (18 mg/kg iv) and activation of the indicated proteins were analyzed by Western-blotting. GAPDH was used as a loading control. **D.** EC-70124 accumulation in xenografted tumor: 4 nu/nu mice implanted with MDA-MB-231 were treated intravenously with 18 mg/Kg EC-70124, after the time specified, two tumors per time point were processed for LC-MS pharmacokinetics analysis. **E.** Phamacokinetic profile of EC-70124 administered by intravenous bolus injection (20 mg/Kg) in CD1 mice, each point represents the average of 3 mice plasma measurements, 1 ng/mL equals 2.2 nM EC-70124.

As success in cancer therapy is based on drug combinations, we investigated the effect of EC-70124 in association with chemotherapies used in the clinical setting for triple negative tumors; including vinorelbine, docetaxel, and carboplatin. We did first a dose response curve with these chemotherapies to select doses around the IC50 (data not shown). Next we combined EC-70124 with these agents. In general, administration of EC-70124 with vinorelbine, carboplatin and docetaxel increased the anti-proliferative effect of each agent given alone (data not shown). To identify synergistic interactions we combined several doses of EC-70124 in the nanomolar range with doses of these agents around or below the IC50 in BT549, HS578T and MDA-MB-231. For this purpose we used the Chou-Talalay algorithm for combination index analysis [27]. Combinations with vinorelbine were antagonistic in MDA-MB-231 and BT549, and only additive for some doses in HS578T (Figure 6A). By contrast most doses for docetaxel and carboplatin were strongly synergistic or additive in the three cell lines (Figure 6A). The strongest synergism for the three cell lines was observed with the combination of EC-70124 with docetaxel. These findings were confirmed by clonogenic analyses using docetaxel and carboplatin. In HS578T, BT549 and MDA-MB-231 the number of colonies formed were reduced with the combined treatments compared with agents given alone (Supplementary Figure S1C).

Given the fact that docetaxel and carboplatin were synergistic, we evaluated the effect on apoptosis of the combination of these agents with EC-70124. The administration of EC-70124 with docetaxel and carboplatin to BT549 and HS578T clearly induced apoptosis at 48 hours (Supplementary Figure S1D). These findings suggest that the addition of some chemotherapies to EC-70124 augmented the cell death obtained with the kinase inhibitor.

### *In vivo* evaluation and pharmacodynamic/pharmacokinetic analyses

Finally we explored the effect of the combination of EC-70124 with carboplatin and docetaxel in xenografted mice. Doses of EC-70124 were reduced to 14 mg/kg alone or in addition to docetaxel and carboplatin both given at 15 mg/kg ip weekly for 3 consecutive weeks. At this dose, EC-70124 showed less activity than at a higher dose, but in the same range as carboplatin alone or the combination EC-70124 + carboplatin. Docetaxel clearly inhibited tumor growth and the combination of EC-70124 with docetaxel reduced tumor volume in a statistically significant manner compared with docetaxel alone (Figure 6B). No differences in mice weight were observed among different groups (data not shown).

To explore the pharmacodynamic effect of EC-70124 *in vivo*, we treated mice injected with MDA-MB-231, and sacrificed the animals at different time points after iv administration of EC-70124 alone. In the extracted tumors we observed a clear reduction of pS6 and pSTAT1 at 30 minutes. pSTAT3 was also slightly affected. Induction of pγH2AX, as a marker of DNA damage, was observed at 60 minutes (Figure 6C). No degradation of PARP was observed and a slight reduction of PCNA, a marker of proliferation was identified at short times (we used the leukemic cell K562 treated with doxorubicin as a positive control for PARP degradation; Supplementary Figure S2A). These data confirm the findings observed in our biochemical studies in cell lines showing that EC-70124 reduced pS6 and STAT1/STAT3, in addition to an induction of DNA damage. In addition, we treated animals with EC-70124 (18 mg/kg iv) for 24 hours and evaluated pS6, Ki67 and TUNEL by immunohistochemistry. We observed a clear reduction of pS6, Ki67; and an induction of apoptosis (Supplementary Figure 3A, 3B).

In parallel we evaluated the concentration of the drug in two resected tumors per time point. Figure 6D shows time-dependent accumulation, reaching over 1000 ng/g (>2.2 μM) well above levels used in successful cell line experiments. Finally, by using another set of animals (healthy CD1 mice) we did a pharmacokinetic evaluation of EC-70124 at 20 mg/kg. As can be seen in Figure 6E, EC-70124 shows plasma levels well above needed for therapeutic action. The pharmacokinetic profile was typical of iv administration, with Cmax >25 μM at 5 min and the levels going exponentially down over time, at 1 h 12 μM, at 4 h 4.5 μM and 8 h 2.2 μM and at 24 h 100 nM. The area under the curve (AUC) was 33 μg/mL/h and the half life 3.1 h.

## DISCUSSION

The prognosis of TNBC patients is poor due to the limited therapeutic options and the lack of specific targeted agents [28]. Given the importance of protein kinases including RTKs or downstream mediators in this subtype of breast cancer, we explored the antitumor activity and mechanism of action of the novel kinase inhibitor EC-70124. In addition, as basal-like tumors show an increase in proliferation, metastatic spreading, early relapse and an impairment of DNA repair mechanisms, it would be desirable to identify a drug that would affect some of these deregulated functions.

Natural products are well known by their pleiotropic mechanism of action. The combinatorial biosynthesis of Rebeccamycin and Staurosporine gene pathways led to EC-70124, a hybrid indolocarbazole analog obtained by recombinant fermentation [24]. Treatment with EC-70124 reduced proliferation and colony formation in several TNBC cell models. Studies in animals showed the anti-tumor activity of EC-70124 *in vivo* with no evidence of relevant toxicity. When the mechanism of action was evaluated, we observed that administration of EC-70124 induced DNA damage at short times measured by the phosphorylation of γH2AX and other proteins including the phosphorylated forms of p53 and Chk2, all of them downstream effectors of ATM and ATR in response to DNA insults [25, 26, 29].

In TNBC several membrane and intracellular kinases are concomitantly activated suggesting that combinatorial strategies or inhibition of central signaling nodes could be more effective than single target inhibition [19, 21]. The PI3K/mTOR and the MAPK pathway are commonly phosphorylated in this tumor type [19]. Less frequent activated kinases include STAT1, STAT3 or SRC, among others [19]. Our data corroborated these findings and furthermore describe the kinase inhibitory profile of EC-70124 on these routes. EC-70124 produced a decrease in phosphorylation of the mTORC1 downstream component pS6. Interestingly, inhibition of the mTORC2 route by EC-70124 resulted distinct in BT549 and HS578T. In the latter cell line, treatment with the drug inhibited AKT phosphorylation at serine 473, a site whose phosphorylation is due to the activity of mTORC2 complexes. In contrast, S473 phosphorylation was unaffected by EC70124 in BT549 cells, indicating that the mTORC2-inhibiting capability of the drug may differ among distinct cell lines. The effect on the JAK pathway was confirmed when we evaluated the kinase profile of the drug. In our experiments doses of the drug in the nanomolar range were able to produce growth inhibition in a panel of TNBC cell lines at the same dose that inhibited efficiently the mentioned routes. Among those the PI3K/mTOR seems to be of substantial importance. In previous experiments inhibition of this pathway in TNBC produced a profound antitumor activity in cell lines and in xenografted tumors [19]. Those findings helped guide the development of inhibitors against this pathway in the clinical setting [19, 30]. EC-70124 inhibited other relevant pathways such as the JAK/STAT; route that is involved in the genesis of breast tumors with stem cell properties, a finding that is in line with the down-regulation of gene-sets related to regulation of stem cell differentiation and the reduction of several stem-cell markers measured with flow cytometry [13]. Of note, no effect on RTK was observed as there was a minimal constitutive phosphorylation in the cell lines. The inhibition of the mentioned proteins were also confirmed in pharmacodynamic experiments showing that the drug reached the tumor at early time points. The presence of the drug was evaluated in two tumors per time point showing a remarkable exposure. Finally pharmacokinetic experiments confirmed a tipical iv elimination curve with concentrations of the drug above the micromolar range at 8 hours.

Gene set enrichment analyses (GSEA) revealed deregulation of many cellular functions including cell morphogenesis; regulation of stem cell differentiation; EMT phenotype, migration and DNA damage, among others. As a multikinase inhibitor with effect on different cancer-relevant kinases, it is expected to observe the inhibition of several functions. Among them, cell migration was inhibited by the drug in the same amount as other kinase inhibitors with known capabilities to reduce this function. Several markers used to identify populations with stem cell characteristics, were also reduced after treatment with EC-70124 [13, 31]. Furthermore, the JAK/STAT pathway, that is required for the growth of stem cell-like breast cancers [13], was also inhibited by EC-70124 in our biochemical studies.

DNA repair was one of the functions altered by the drug. At longer times we observed an increased expression of genes involved in DNA repair; genes that probably were synthesized in response to a DNA insult. This observation was confirmed by qPCR of the upregulated genes including BRCA2, LIG4, GADD45 or XPA, among others. Although induction of DNA damage is an infrequent effect of kinase inhibitors, we have observed before that the combination of dasatinib, a multi-TK inhibitor, with trastuzumab, an anti-HER2 antibody, produced a similar effect on DNA [32].

Physiological repair of DNA damage requires cells to arrest [17]. In our experiment we observed by flow cytometry how treatment with EC-70124 induced an arrest in G2/M. Induction of apoptosis evaluated by Annexin V staining was observed at 48 hours suggesting that some cells were unable to repair their DNA and finally led to cell death. This induction of apoptosis was largely independent of the activation of caspases. In addition, combination of EC-70124 with chemotherapy increase apoptosis compared with each agent given alone.

When combined with chemotherapies, EC-70124 produced a synergistic effect with docetaxel opening the door for potential combinations with these agents in the clinical setting. Taxanes are one of the gold standards in the treatment of this disease. In line with this, the studies in animals showed that EC-70124 increased the antitumor effect of docetaxel, compared with the effect of each agent given alone. A much limited effect was observed with the combination of carboplatin.

Globally, our article describes a novel multi-kinase inhibitor with a remarkable antitumor activity in TNBC. The pleiotropic nature of the effects of this drug, which affects cell cycle progression, migration or stem-like properties of TNBC cells offers the possibility of acting on all those oncogenic pathways using a single agent. Moreover, its synergistic effect with drugs that are commonly used to treat TBNC patients opens the possibility for the development of EC-70124 alone or in combination with those drugs in the clinical setting.

## MATERIALS AND METHODS

### Cell culture and drug compounds

HS578T, BT549, MDA-MB-231 and HCC3153 breast cancer cell lines were provided by Drs J Losada and A Balmain (originally from Dr. JW Gray's Laboratory, who in turn obtained them from the ATCC or from collection development in the laboratories of Drs S Ethier and A Gazdar to avoid errors occurring when obtained through ‘second-hand’ sources). In addition cells were analyzed by STR at the molecular biology unit at the Salamanca University Hospital. Cell lines were maintained in DMEM (HS578T, BT549, MDA-MB-231) and RPMI (HCC3153) containing 10% fetal bovine serum (FBS), with 100 U/mL penicillin, 100 μg/mL streptomycin and 2 mM L-glutamine, respectively. The cell culture medium and supplements were obtained from Sigma Aldrich (St. Louis, MO). The multi-kinase inhibitor EC-70124 was prepared via a proprietary process by Entrechem S.L. (Oviedo, Spain). Chemotherapeutic agents (Docetaxel, Carboplatin and Vinorelbine) were purchased from Selleckchem.

### Cell proliferation studies: MTT assays, matrigel-embedded culture experiments and clonogenic assays

The effect of drugs on cell proliferation was assessed using MTT (3-(4, 5-dimethylthiazol-2-yl)-2, 5 diphenyltetrazolium bromide) screening assay. This experiment is based on the ability of living cells to convert MTT, a yellow tetrazole, into a purple colored formazan product with an absorbance maximum near 570 nm. Thus, a decrease in the amount cells amount will result in a reduced purple colouring. HS578T, BT549, MDA-MB-231 and HCC3153 cells were plated at 10,000 cells per well in 48-multiwell plates and cultured overnight in DMEM and RPMI + 10% FBS. The next day, cells were treated for three days with increasing concentrations of EC-70124 alone or in combination with various chemotherapeutics to plot the dose–response curves and for synergy studies, respectively. In parallel, the IC50 value was determined by cell treatment with increasing amounts of EC-70124 at three-time points (24 h, 48 h and 72 h of incubation). After drug administration, the medium was replaced with 400 μL of fresh medium DMEM without phenol red containing MTT (0.5 μg/μL) and incubated for 45 minutes at 37°C. The medium was then removed and 200 μL of dimethylsulfoxide (DMSO) were added to each well. The plate was agitated in the dark for 5 minutes to dissolve the MTT-formazan crystals. The absorbance of the samples was recorded at 562 nm (555–690) in a multiwell plate reader (BMG labtech). Results were plotted as the mean values of quadruplicates from a representative experiment that was repeated at least two independent times.

For drug combination studies, synergy was confirmed using Calcusyn Version 2.0 software (Biosoft, Ferguson, MO) by determining combinational index (CI) based on the algorithm reported by Chou and Talalay [27]. Values <1 represent synergistic effect on cell proliferation of the 2 drugs, values equal to 1 indicate additive effect of the drugs, and values >1 represent an antagonistic effect. Combination index values from three independent experiments were generated and plotted.

For three-dimensional cell culture experiments, HS578T, BT549 and MDA-MB-231 cells were grown in DMEM supplemented with 10% FBS. Following passage, cells were trypsinized (0.5 g porcine trypsin and 0.2 g EDTA 4Na, purchased from Sigma Aldrich) and resuspended in growth medium containing 2% Matrigel. Then, cells were seeded at a density of 12.500 cells/ml in a 48-multiwell plate containing an underlying approximately 1 mm thick bed of Matrigel and incubated at 37°C. Next day, cells were treated with EC-70124 and cultured for 7 days. The assay included the daily visualization of cells under a light microscope to monitor the phenotype.

For clonogenic studies, cells were seeded at a density of 500,000 cells in a 100 mm culture dishes, and treated, the next day, with with EC-70124 (300 nM), docetaxel (0.5 nM), EC-70124 + docetaxel, carboplatin (20 μM) and EC-70124 + Carboplatin. After 24-hours treatment, cells were trypsinized, resuspended in 5 ml of complete growth medium to perform serial dilutions 1/10 and seeded, in triplicate, in 6-multiwell plates for 10 days. Then, the medium was removed and the number of colonies were determined.

### Wound-healing assay for cell migration

HS578T, BT549 and MDA-MB-231 cells were plated at a density of 300,000 cells/60 mm dish and maintained overnight in DMEM + 10% FBS. Following incubation, culture medium was removed and a wound in the cell monolayers was generated by scratching with a 200-μl pipette tip. Photographs were taken of the initial wound for comparison. Then, DMEM + 10% FBS was added and cells were treated for 48 hours with either 500 nM EC-70124 or 100 nM Dasatinib, as a negative control. Cell migration was visualized at ×10 magnification and photographed. Each experiment was completed in duplicate.

### Cell cycle and apoptosis detection assays

For cell cycle analysis, HS578T, BT549 and MDA-MB-231 cells were plated at a density of 500,000 cells/100 mm dish and maintained overnight in DMEM + 10% FBS. Cells were then treated with 500 nM of EC-70124 for 24 hours. After drug treatment, cells were trypsinized, fixed in ice cold 70% ethanol for 30 minutes and subsequently centrifuged at 5000 rpm for 5 minutes. Cell pellets were washed in PBS + 2% BSA and treated with Propidium iodide/RNAse staining solution (Immunostep S.L., Salamanca, Spain) in the dark for 1 hour at 4°C, and analyzed then on FACSCanto II flow cytometer (BD Biosciences). The percentage of each cell cycle phase was determined by plotting DNA content against cell number using the FACS Diva software.

For Annexin V/PI experiments, 500 nM EC-70124 treated cells (at a density of 500,000 cells/100 mm dish) were washed twice with PBS, and stained with 5 μl of Annexin V-DT-634 (Immunostep S.L., Salamanca, Spain) and 3 μl of Propidium iodide (10 mg/ml) in 1x Binding Buffer (10 mM HEPES, pH 7.4, 140 mM NaOH, 2.5 mM CaCl_2_) for 1 hour at room temperature in the dark. The apoptotic cells were determined using a FACSCanto II flow cytometer (BD Biosciences). Both early apoptotic (Annexin V-positive, PI-negative) and late (Annexin V-positive and PI-positive) apoptotic cells were included in cell death determinations.

### Preparation of cell extracts and western-blotting

HS578T and BT549 cells were plated at a density of 500,000 cells/100 mm dish, maintained overnight in DMEM + 10% FBS, and treated later with EC-70124 at 500 nM for 6, 12 and 24 hours. After treatment, cells were washed with cold PBS and lysed in cold lysis buffer (20 mM Tris–HCl [pH 7.0], 140 mM NaCl, 50 mM EDTA, 10% glycerol, 1% Nonidet P-40, 1 μM pepstatin, 1 μg/mL aprotinin, 1 μg/mL leupeptin, 1 mM phenylmethyl sulfonyl fluoride, 1 mM sodium orthovanadate). Then, insoluble material was removed by centrifugation. The protein concentration was determined using BCA (Bicinchoninic acid) protein assay kit (Sigma Aldrich).

For Western-blotting, 50 μg protein was resolved by 6%–15% sodium dodecyl sulfate polyacrylamide gel electrophoresis (SDS-PAGE) and transferred to polyvinylidene difluoride membranes (Millipore Corporation). Blots were blocked in 1x Tris-buffered saline (TBS, 100 mM Tris [pH 7.5], 150 mM NaCl, 0.05% Tween 20) containing 0.05% Tween 20 and 1% of bovine serum albumin for 1 hour and then incubated overnight with the following primary human monoclonal/policlonal antibodies: anti-p^S473^-AKT, anti-pH2AX (BD Biosciences), anti-pERK1/2, PARP (Santa Cruz Biotechnology), anti-pS6, anti-pSTAT3, anti-pSTAT1, anti-p^T308^-AKT (Cell Signalling Technology). Protein-bound primary antibodies were detected using respective horseradish peroxidase-coupled secondary antibodies (anti-rabbit for polyclonal and anti-mouse for monoclonal, obtained from Santa Cruz Biotechnology) diluted 1:5,000 in 1x TBS containing 0.05% Tween and incubated for 1 hour at room temperature. Protein bands were detected using ECL Plus Western Blotting Detection System (GE Healthcare, Buckinghamshire, United Kingdom).

### Phospho-kinase antibody array and immunohistochemistry

To evaluate whether EC-70124 treatment causes a change in the kinase phosphorylation profiling of human breast cancer cell, two different commercial arrays, the human phospho-RTK array kit (# ARY001, R&D Systems, Abingdon, UK) and the PathScan RTK Signaling Antibody Array Kit (# 7982, Cell Signaling Technology) were used. The array kits allows for the simultaneous detection of receptor tyrosine kinases and downstream signaling nodes, when phosphorylated at tyrosine or other residues. Target-specific capture antibodies, biotinylated protein (positive control) and non-specific IgG (negative control) have been spotted in duplicate onto the array membrane. Cell lysates were diluted to a total protein concentration and incubated with the RTK arrays according to manufacturer's instructions. After binding the domain of both phosphorylated and unphophorylated tyrosine kinases, unbound material was washed away. A pan anti-phospho-tyrosine antibody conjugated to horseradish peroxidase (HRP) was used to detect phosphorylated tyrosines on activated receptors by chemiluminescence. Quantitation of the different RTKs and cell signaling intermediates in the human phospho-RTK array kit was performed using the Quantity One analysis software (Bio-Rad, CA, USA) and expressed as the pixel density (OD/mm^2^). The pixel density of the background was subtracted from the pixel density of each spot, and the average of duplicate spots was determined. Next, Signal intensity was calculated by the normalization of mean pixel density in each spot against the pixel density of the positive control. Significance was determined using a cut-off point of density signal higher than 0.6.

For the immunohistochemistry evaluation, tumour samples from mice were obtained and fixed with formaldehyde 24 hours at room temperature. After the samples were embedded in paraffin, they were cut into thin slices and mounted on slides for subsequent analysis. The immunohistochemistry for ki67, pS6 were performed as described [33, 34] and TUNEL was performed following the manufacturer's instructions (*In situ* cell Death Detection Kit, AP, Roche).

### Microarray analysis of mRNA

MDA-MB-231 cells were grown in DMEM with 10% of FBS and added to a final concentration of 500,000 cells/plate. Next day cells were treated with 500 nM EC-70124 for 24 h. Total RNA from 3 independent control samples and 3 independent treated cells was extracted and purified using the RNeasy Mini Kit (Qiagen). Double-stranded cDNA and biotinylated cRNA were synthesized using a T7 polyT primer and the BioArray RNA labeling kit (Enzo Life Sciences, Farmingdale, NY), respectively. The labeled RNA was fragmented and hybridized to human oligonucleotide arrays (Human Gene ST Arrays) (Affymetrix, Santa Clara, CA) according to the manufacturer's instructions. Affymetrix CEL files were imported into the dChip software (Dana Farber Cancer Institute, Boston, MA): normalization of all arrays was done using a pair-wise rank invariant probe method and expression level of each probeset was calculated using the model-based expression index (MBEI).

### Gene-set enrichment analysis

The MBEI data for all probesets were further imported into the GSEA software [26] to perform gene-set enrichment analysis using 2000 gene-set permutations, minimum gene-set size of 5, maximum gene-set size of 500 and *t*-test as the scoring metric. The gene-sets included in the GSEA analyses were obtained from KEGG, MsigDB-c2, NCI, Biocarta, IOB, Netpath, HumanCyc, Reactome and the Gene Ontology (GO) databases, updated August 2014 (http://baderlab.org/GeneSets). EnrichmentMap (version 2.0, automatic labelling option, Cytoscape 3.2.1) was used to visualize enriched gene-sets with a nominal *p*-value < 0.025 and a False Discover Rate < 0.25.

### Quantitative reverse-transcription PCR (qRT-PCR)

Untreated and EC-70124-treated cells were collected and mRNA levels of the indicated genes were determined by quantitative reverse- transcription PCR. Briefly, total RNA was obtained from cells using RNeasy Mini Kit (Qiagen, Hilden, Germany) according to manufacturer's instruction. After extraction, concentration and purity were determined using a NanoDrop ND-1000 spectrophotometer (Thermo Fisher Scientific, USA) and, subsequently, 3 μg of total RNA was reversely transcribed using RevertAid H Minus First Strand cDNA syntesis Kit (Thermo Fisher Scientific, USA) in a thermocycler (Bio-Rad) under the following reaction conditions: 65°C for 5 min, 42°C for 60 min and 70°C for 10 min. The cDNAs were then subjected to a real-time PCR analysis using Fast SYBR Green Master Mix in StepOnePlus Real-Time PCR system (Applied Biosystems) according to the manufacturer's instructions. Primer sequences for the genes are shown in Supplementary Table S3. An initial step was performed at 95°C for 10 min, followed by 40 cycles of 95°C for 15 sec and finished by 60°C for 1min. each sample was analyzed in triplicates and cycle threshold (Ct) values of transcripts was determined using StepOne Software v.2.1. The ΔCt values were calculated using GAPDH as reference. Untreated cells were used as control to calculate the ΔΔCt value and to determine the X-fold mRNA expression.

### Xenograft studies

Mice were handled at the animal facility following legal guidelines. Female BALB/cAnNRi-Foxn1nu/Foxn1nu mice, 5 weeks old were obtained from Janvier Labs. After 10 days quarantine, 2–5 × 10^6^ MDA-MB-231 cells in 100 μL of DMEM with 20% Matrigel were injected into the mammary fat pads of mice. Two weeks after the injection, mice were randomly assigned into two groups (with equal average tumor volumes before initiation of treatments) control vehicle (manufacturer recommendations) (*n* = 5) and EC-70124 + vehicle (*n* = 5). After approximately 2 weeks, when tumors reached a volume of 100 mm^3^ treatment was initiated. Animals were inhalatorily anaesthetized and then treated with EC70124 i.v. (18 mg/kg) every three days. For combinatory studies, when tumors reached a volume of 200 mm^3^ treatment was initiated, in this case, we used EC-70124 (14 mg/kg) every three days alone or in combination with carboplatin (15 mg/kg) or docetaxel (15 mg/kg) ip weekly for 3 consecutive weeks (*n* = 5 for all groups). Tumors diameters were measured every three days and tumor volumes were calculated using the following formula: V = (L × W^2^)/2, where V = volume (cubic millimeters), L = length (millimeters) and *W* = width (millimeters). Mice were killed by CO_2_ inhalation.

### Pharmacokinetics

Healthy CD-1 female mice were dosed orally with EC-70124 (18 mg/Kg). Blood was collected at eight time-points between 5 min and 24 h. Each time point represented the average of 3 mice. Serum was diluted with 1 volume of methanol and centrifuged to eliminate any insoluble precipitate. The supernatant was then used to measure EC-70124 by LC-MS.

## SUPPLEMENTARY FIGURES AND TABLES






