# Supplementary material for: Antitumor activity of the novel multi-kinase inhibitor EC-70124 in triple negative breast cancer
Source: Oncotarget. 2015 Aug 12;6(29):27923–37. doi: 10.18632/oncotarget.4736 (PMC4695035; doi:10.18632/oncotarget.4736)
Supplement: Supplementary file 1 [file oncotarget-06-27923-s001.pdf]

SUPPLEMENTARY FIGURES AND TABLES

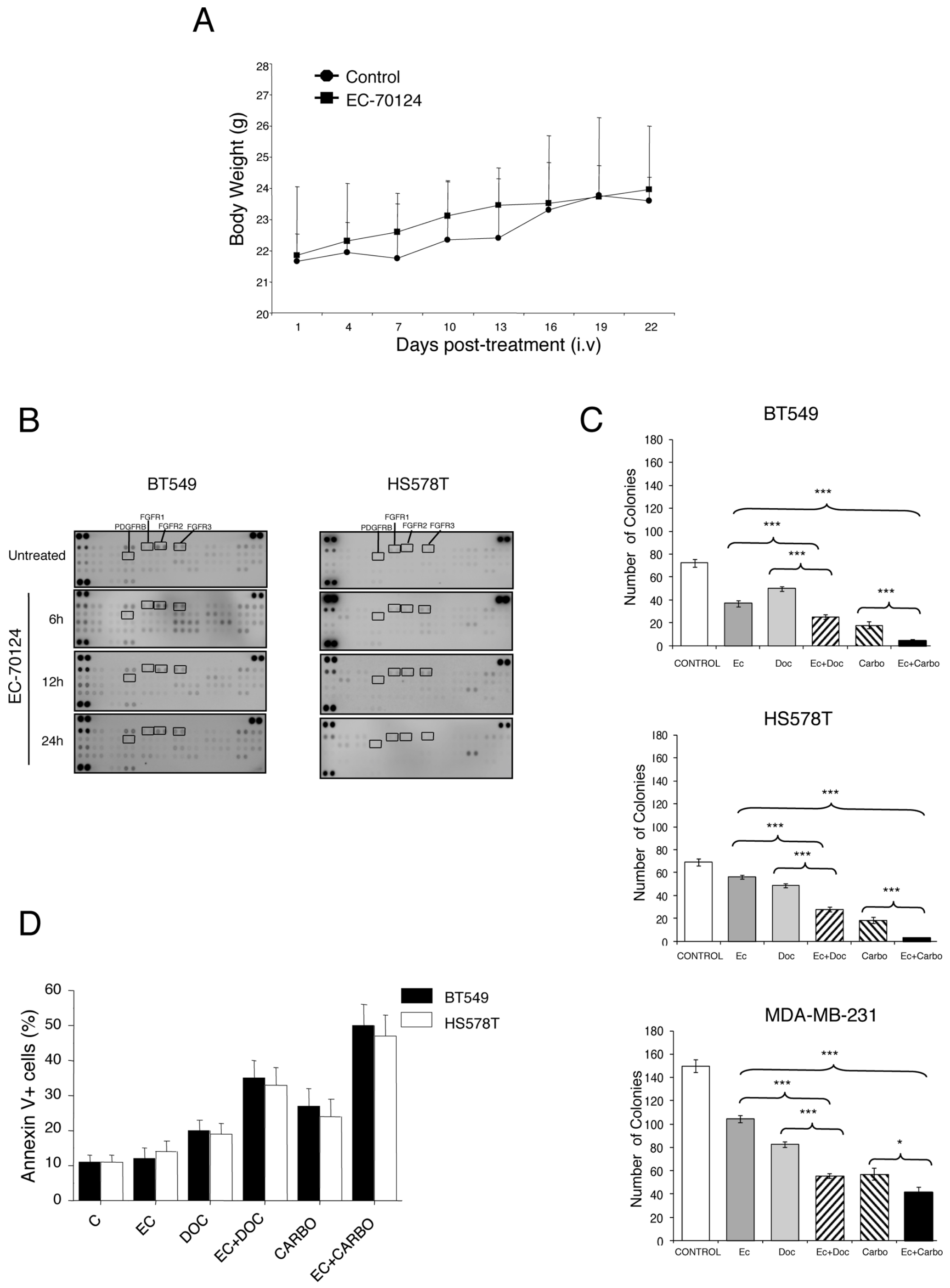

Supplementary Figure S1: A. Effect of EC-70124 administration on body weight of untreated and treated animals. Weight was measured every three days. Animals were inhalatorily anaesthetized and then treated with EC70124 i.v. (18 mg/kg) every three days. **B.** Analysis of Receptor tyrosine kinase activation in BT549 and HS578T; and effect of EC-70124 on their phosphorylated status. Cell lysates were examined for the phosphorylation of a panel 42 extracellular kinases using an antibody array described in “Material and methods”. **C.** Effect on colony formation of EC-70124 alone or in combination with chemotherapies in HS578T, BT549 and MDA-MB-231. Cells were treated with EC-70124 (300 nM), docetaxel (0.5 nM), and carboplatin (20 µM) for 5 hours and number of colonies was determined following 10 days of treatment. Bart charts represent the number of colonies formed in relation to untreated cells. **D.** Evaluation of apoptosis induction by EC-70124, carboplatin or docetaxel therapies alone or in combination in BT549 and HS578T. Cells were treated with EC-70124 (300 nM), docetaxel (1.6 nM), and carboplatin (20 µM), and stained with Annexin V after 48 hours of treatment. The histogram represents the mean percentage of cells positive or negative to Annexin V staining.

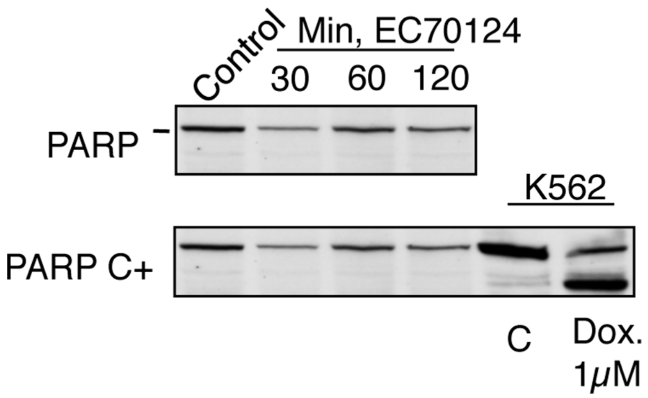

Supplementary Figure S2: Effect of EC-70124 on expression of PARP in mice tumors. Animals were treated for 30, 60 and 120 minutes with EC-70124 (18 mg/kg iv) and expression of PARP degradation was analyzed by Western-blotting. The leukemic cell K562 treated with doxorubicin (1 μM) was used as a positive control.

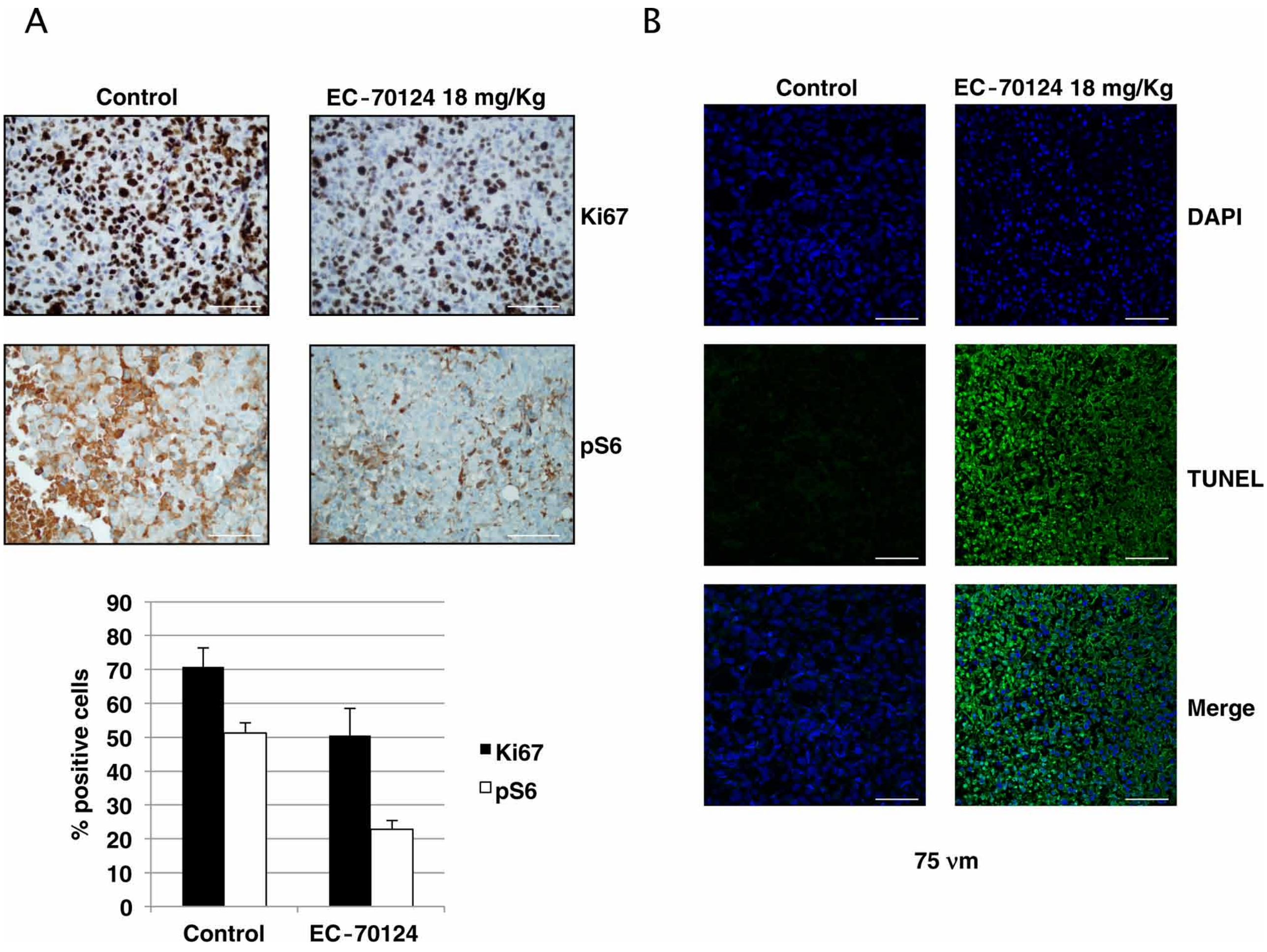

Supplementary Figure S3: Effect of EC-70124 on expression of Ki67, pS6 and TUNEL in tumors from treated mice at 24 hours with EC-70124 (18 mg/kg iv). Antibodies used are described in “Material and methods” Bar, 75 microm.

Supplementary Table S1: GSEA analyses including information regarding gene-set name, NES, *p*-value and FDR *q* values

Supplementary Table S2: List of selected genes modified by EC-70124 in treated samples compared with untreated ones, that belong to the gene-sets represented in figure B, C, and D

Supplementary Table S3: Primer sequences used for quantitative reverse-transcription PCR analyses of indicated genes related to DNA damage in untreated and treated MDA-MB-231 cells (500 nM), at different time points (6, 12, 24 hours)

GENES	PRIMERS	SEQUENCE
**BRCA1**	Forward	5′-ACTCTGGGGCTCTGTCTTCA-3′
	Reverse	5′-GGTGGTACATGCACAGTTGC-3′
**LIG4**	Forward	5′-GTCTGGGCCTGGATTTTGTA-3′
	Reverse	5′-TGCCCCAAAGATGAAGAAAG-3′
**FANCF**	Forward	5′-GGACTCAGTTCCAACCCAAA-3′
	Reverse	5′-GCTAGTCCACTGGCTTCTGG-3′
**RAD52**	Forward	5′-AGTTTTGGGAATGCATTGG-3′
	Reverse	5′-TCGGCAGCTGTTGTATCTTG-3′
**PRMT6**	Forward	5′-CTCTTCATAGCCCCCATCAG-3′
	Reverse	5′-AATCCCTGCACAACGATCTC-3′
**BRCA2**	Forward	5′-CCAATGCCTCGTAACAACCT-3′
	Reverse	5′-AGCTCTTCACCCTGCAAAAA-3′
**DDB2**	Forward	5′-GTGACCACCATTCGGCTACT-3′
	Reverse	5′-TCAAGGACAAACCCACCTTC-3′
**GADD45A**	Forward	5′-GGAGGAAGTGCTCAGCAAAG-3′
	Reverse	5′-TGGATCAGGGTGAAGTGGAT-3′
**XPA**	Forward	5′-GCAGCCCCAAAGATAATTGA-3′
	Reverse	5′- TGGCAAATCAAAGTGGTTCA-3′
**GADPH**	Forward	5′-CAATGACCCCTTCATTGACC-3′
	Reverse	5′- GATCTCGCTCCTGGAAGATG-3′
