# Supplementary material for: Antitumor activity of the novel multi-kinase inhibitor EC-70124 in triple negative breast cancer
Source: Oncotarget. 2015 Aug 12;6(29):27923–37. doi: 10.18632/oncotarget.4736 (PMC4695035; doi:10.18632/oncotarget.4736)
Supplement: Supplementary file 2 [file oncotarget-06-27923-s002.pptx]

## Slide 1
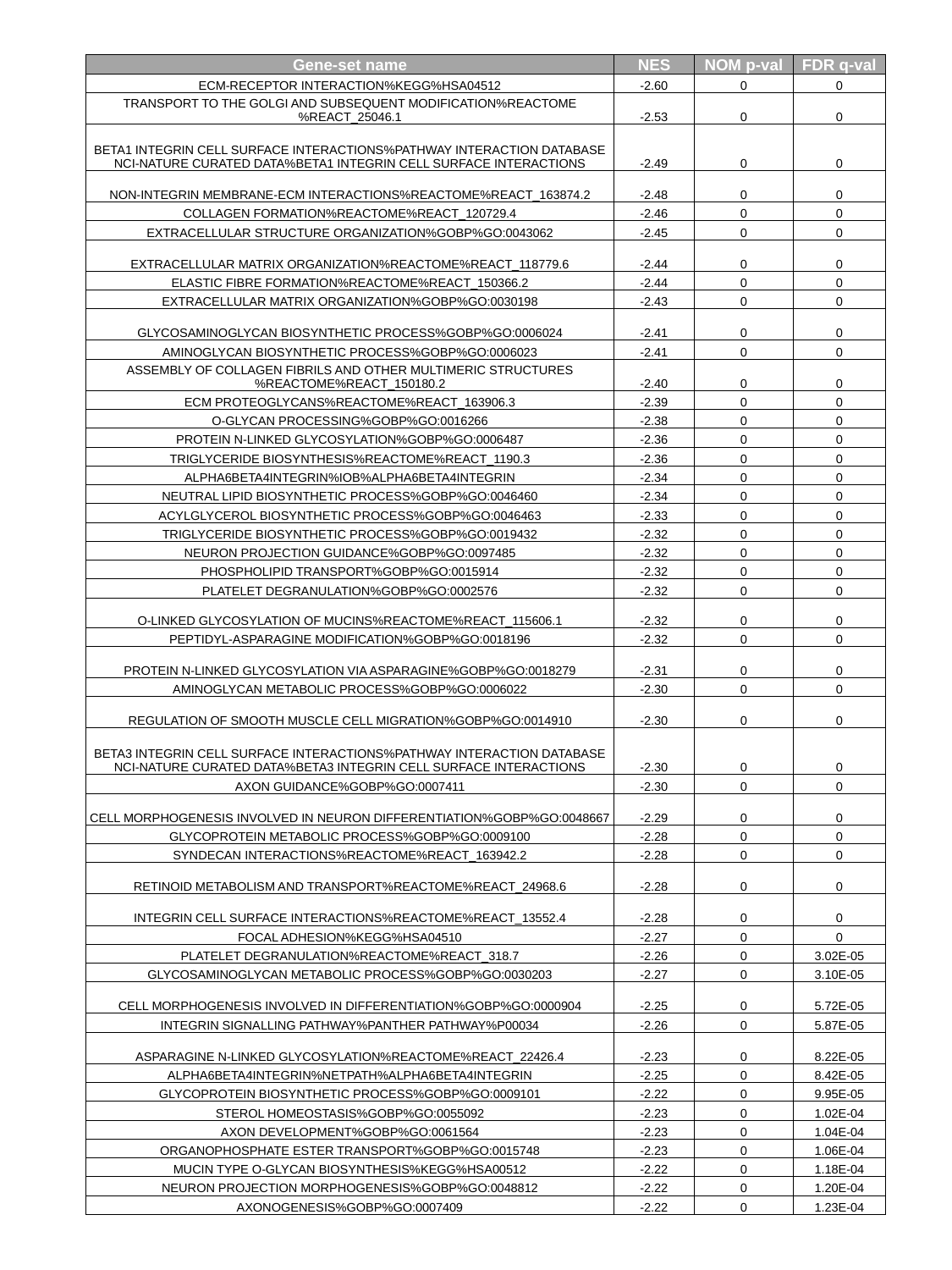

| Gene-set name | NES | NOM p-val | FDR q-val |
| --- | --- | --- | --- |
| ECM-RECEPTOR INTERACTION%KEGG%HSA04512 | -2.60 | 0 | 0 |
| TRANSPORT TO THE GOLGI AND SUBSEQUENT MODIFICATION%REACTOME%REACT\_25046.1 | -2.53 | 0 | 0 |
| BETA1 INTEGRIN CELL SURFACE INTERACTIONS%PATHWAY INTERACTION DATABASE NCI-NATURE CURATED DATA%BETA1 INTEGRIN CELL SURFACE INTERACTIONS | -2.49 | 0 | 0 |
| NON-INTEGRIN MEMBRANE-ECM INTERACTIONS%REACTOME%REACT\_163874.2 | -2.48 | 0 | 0 |
| COLLAGEN FORMATION%REACTOME%REACT\_120729.4 | -2.46 | 0 | 0 |
| EXTRACELLULAR STRUCTURE ORGANIZATION%GOBP%GO:0043062 | -2.45 | 0 | 0 |
| EXTRACELLULAR MATRIX ORGANIZATION%REACTOME%REACT\_118779.6 | -2.44 | 0 | 0 |
| ELASTIC FIBRE FORMATION%REACTOME%REACT\_150366.2 | -2.44 | 0 | 0 |
| EXTRACELLULAR MATRIX ORGANIZATION%GOBP%GO:0030198 | -2.43 | 0 | 0 |
| GLYCOSAMINOGLYCAN BIOSYNTHETIC PROCESS%GOBP%GO:0006024 | -2.41 | 0 | 0 |
| AMINOGLYCAN BIOSYNTHETIC PROCESS%GOBP%GO:0006023 | -2.41 | 0 | 0 |
| ASSEMBLY OF COLLAGEN FIBRILS AND OTHER MULTIMERIC STRUCTURES%REACTOME%REACT\_150180.2 | -2.40 | 0 | 0 |
| ECM PROTEOGLYCANS%REACTOME%REACT\_163906.3 | -2.39 | 0 | 0 |
| O-GLYCAN PROCESSING%GOBP%GO:0016266 | -2.38 | 0 | 0 |
| PROTEIN N-LINKED GLYCOSYLATION%GOBP%GO:0006487 | -2.36 | 0 | 0 |
| TRIGLYCERIDE BIOSYNTHESIS%REACTOME%REACT\_1190.3 | -2.36 | 0 | 0 |
| ALPHA6BETA4INTEGRIN%IOB%ALPHA6BETA4INTEGRIN | -2.34 | 0 | 0 |
| NEUTRAL LIPID BIOSYNTHETIC PROCESS%GOBP%GO:0046460 | -2.34 | 0 | 0 |
| ACYLGLYCEROL BIOSYNTHETIC PROCESS%GOBP%GO:0046463 | -2.33 | 0 | 0 |
| TRIGLYCERIDE BIOSYNTHETIC PROCESS%GOBP%GO:0019432 | -2.32 | 0 | 0 |
| NEURON PROJECTION GUIDANCE%GOBP%GO:0097485 | -2.32 | 0 | 0 |
| PHOSPHOLIPID TRANSPORT%GOBP%GO:0015914 | -2.32 | 0 | 0 |
| PLATELET DEGRANULATION%GOBP%GO:0002576 | -2.32 | 0 | 0 |
| O-LINKED GLYCOSYLATION OF MUCINS%REACTOME%REACT\_115606.1 | -2.32 | 0 | 0 |
| PEPTIDYL-ASPARAGINE MODIFICATION%GOBP%GO:0018196 | -2.32 | 0 | 0 |
| PROTEIN N-LINKED GLYCOSYLATION VIA ASPARAGINE%GOBP%GO:0018279 | -2.31 | 0 | 0 |
| AMINOGLYCAN METABOLIC PROCESS%GOBP%GO:0006022 | -2.30 | 0 | 0 |
| REGULATION OF SMOOTH MUSCLE CELL MIGRATION%GOBP%GO:0014910 | -2.30 | 0 | 0 |
| BETA3 INTEGRIN CELL SURFACE INTERACTIONS%PATHWAY INTERACTION DATABASE NCI-NATURE CURATED DATA%BETA3 INTEGRIN CELL SURFACE INTERACTIONS | -2.30 | 0 | 0 |
| AXON GUIDANCE%GOBP%GO:0007411 | -2.30 | 0 | 0 |
| CELL MORPHOGENESIS INVOLVED IN NEURON DIFFERENTIATION%GOBP%GO:0048667 | -2.29 | 0 | 0 |
| GLYCOPROTEIN METABOLIC PROCESS%GOBP%GO:0009100 | -2.28 | 0 | 0 |
| SYNDECAN INTERACTIONS%REACTOME%REACT\_163942.2 | -2.28 | 0 | 0 |
| RETINOID METABOLISM AND TRANSPORT%REACTOME%REACT\_24968.6 | -2.28 | 0 | 0 |
| INTEGRIN CELL SURFACE INTERACTIONS%REACTOME%REACT\_13552.4 | -2.28 | 0 | 0 |
| FOCAL ADHESION%KEGG%HSA04510 | -2.27 | 0 | 0 |
| PLATELET DEGRANULATION%REACTOME%REACT\_318.7 | -2.26 | 0 | 3.02E-05 |
| GLYCOSAMINOGLYCAN METABOLIC PROCESS%GOBP%GO:0030203 | -2.27 | 0 | 3.10E-05 |
| CELL MORPHOGENESIS INVOLVED IN DIFFERENTIATION%GOBP%GO:0000904 | -2.25 | 0 | 5.72E-05 |
| INTEGRIN SIGNALLING PATHWAY%PANTHER PATHWAY%P00034 | -2.26 | 0 | 5.87E-05 |
| ASPARAGINE N-LINKED GLYCOSYLATION%REACTOME%REACT\_22426.4 | -2.23 | 0 | 8.22E-05 |
| ALPHA6BETA4INTEGRIN%NETPATH%ALPHA6BETA4INTEGRIN | -2.25 | 0 | 8.42E-05 |
| GLYCOPROTEIN BIOSYNTHETIC PROCESS%GOBP%GO:0009101 | -2.22 | 0 | 9.95E-05 |
| STEROL HOMEOSTASIS%GOBP%GO:0055092 | -2.23 | 0 | 1.02E-04 |
| AXON DEVELOPMENT%GOBP%GO:0061564 | -2.23 | 0 | 1.04E-04 |
| ORGANOPHOSPHATE ESTER TRANSPORT%GOBP%GO:0015748 | -2.23 | 0 | 1.06E-04 |
| MUCIN TYPE O-GLYCAN BIOSYNTHESIS%KEGG%HSA00512 | -2.22 | 0 | 1.18E-04 |
| NEURON PROJECTION MORPHOGENESIS%GOBP%GO:0048812 | -2.22 | 0 | 1.20E-04 |
| AXONOGENESIS%GOBP%GO:0007409 | -2.22 | 0 | 1.23E-04 |

## Slide 2
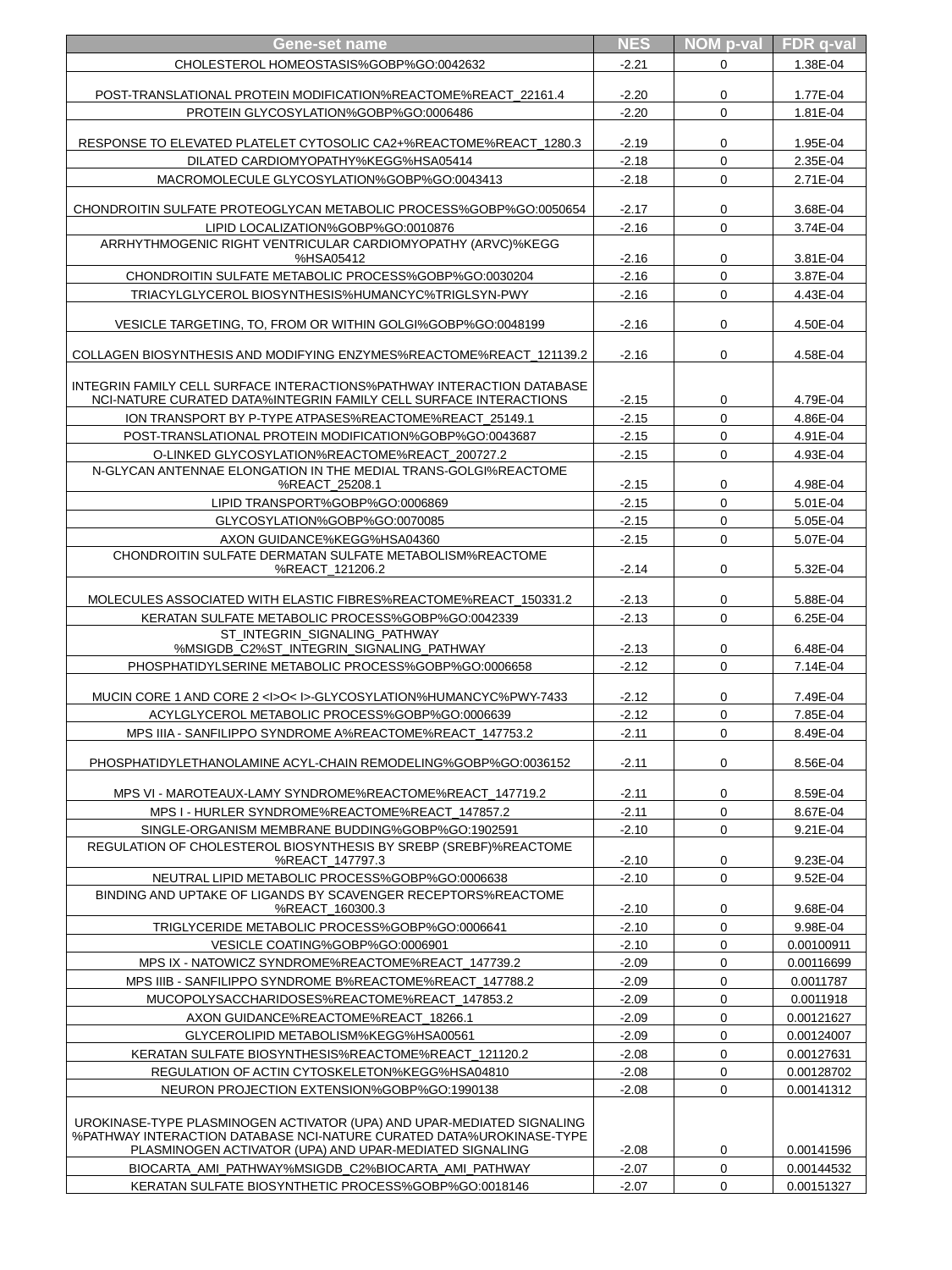

| Gene-set name | NES | NOM p-val | FDR q-val |
| --- | --- | --- | --- |
| CHOLESTEROL HOMEOSTASIS%GOBP%GO:0042632 | -2.21 | 0 | 1.38E-04 |
| POST-TRANSLATIONAL PROTEIN MODIFICATION%REACTOME%REACT\_22161.4 | -2.20 | 0 | 1.77E-04 |
| PROTEIN GLYCOSYLATION%GOBP%GO:0006486 | -2.20 | 0 | 1.81E-04 |
| RESPONSE TO ELEVATED PLATELET CYTOSOLIC CA2+%REACTOME%REACT\_1280.3 | -2.19 | 0 | 1.95E-04 |
| DILATED CARDIOMYOPATHY%KEGG%HSA05414 | -2.18 | 0 | 2.35E-04 |
| MACROMOLECULE GLYCOSYLATION%GOBP%GO:0043413 | -2.18 | 0 | 2.71E-04 |
| CHONDROITIN SULFATE PROTEOGLYCAN METABOLIC PROCESS%GOBP%GO:0050654 | -2.17 | 0 | 3.68E-04 |
| LIPID LOCALIZATION%GOBP%GO:0010876 | -2.16 | 0 | 3.74E-04 |
| ARRHYTHMOGENIC RIGHT VENTRICULAR CARDIOMYOPATHY (ARVC)%KEGG%HSA05412 | -2.16 | 0 | 3.81E-04 |
| CHONDROITIN SULFATE METABOLIC PROCESS%GOBP%GO:0030204 | -2.16 | 0 | 3.87E-04 |
| TRIACYLGLYCEROL BIOSYNTHESIS%HUMANCYC%TRIGLSYN-PWY | -2.16 | 0 | 4.43E-04 |
| VESICLE TARGETING, TO, FROM OR WITHIN GOLGI%GOBP%GO:0048199 | -2.16 | 0 | 4.50E-04 |
| COLLAGEN BIOSYNTHESIS AND MODIFYING ENZYMES%REACTOME%REACT\_121139.2 | -2.16 | 0 | 4.58E-04 |
| INTEGRIN FAMILY CELL SURFACE INTERACTIONS%PATHWAY INTERACTION DATABASE NCI-NATURE CURATED DATA%INTEGRIN FAMILY CELL SURFACE INTERACTIONS | -2.15 | 0 | 4.79E-04 |
| ION TRANSPORT BY P-TYPE ATPASES%REACTOME%REACT\_25149.1 | -2.15 | 0 | 4.86E-04 |
| POST-TRANSLATIONAL PROTEIN MODIFICATION%GOBP%GO:0043687 | -2.15 | 0 | 4.91E-04 |
| O-LINKED GLYCOSYLATION%REACTOME%REACT\_200727.2 | -2.15 | 0 | 4.93E-04 |
| N-GLYCAN ANTENNAE ELONGATION IN THE MEDIAL TRANS-GOLGI%REACTOME%REACT\_25208.1 | -2.15 | 0 | 4.98E-04 |
| LIPID TRANSPORT%GOBP%GO:0006869 | -2.15 | 0 | 5.01E-04 |
| GLYCOSYLATION%GOBP%GO:0070085 | -2.15 | 0 | 5.05E-04 |
| AXON GUIDANCE%KEGG%HSA04360 | -2.15 | 0 | 5.07E-04 |
| CHONDROITIN SULFATE DERMATAN SULFATE METABOLISM%REACTOME%REACT\_121206.2 | -2.14 | 0 | 5.32E-04 |
| MOLECULES ASSOCIATED WITH ELASTIC FIBRES%REACTOME%REACT\_150331.2 | -2.13 | 0 | 5.88E-04 |
| KERATAN SULFATE METABOLIC PROCESS%GOBP%GO:0042339 | -2.13 | 0 | 6.25E-04 |
| ST\_INTEGRIN\_SIGNALING\_PATHWAY%MSIGDB\_C2%ST\_INTEGRIN\_SIGNALING\_PATHWAY | -2.13 | 0 | 6.48E-04 |
| PHOSPHATIDYLSERINE METABOLIC PROCESS%GOBP%GO:0006658 | -2.12 | 0 | 7.14E-04 |
| MUCIN CORE 1 AND CORE 2 <I>O< I>-GLYCOSYLATION%HUMANCYC%PWY-7433 | -2.12 | 0 | 7.49E-04 |
| ACYLGLYCEROL METABOLIC PROCESS%GOBP%GO:0006639 | -2.12 | 0 | 7.85E-04 |
| MPS IIIA - SANFILIPPO SYNDROME A%REACTOME%REACT\_147753.2 | -2.11 | 0 | 8.49E-04 |
| PHOSPHATIDYLETHANOLAMINE ACYL-CHAIN REMODELING%GOBP%GO:0036152 | -2.11 | 0 | 8.56E-04 |
| MPS VI - MAROTEAUX-LAMY SYNDROME%REACTOME%REACT\_147719.2 | -2.11 | 0 | 8.59E-04 |
| MPS I - HURLER SYNDROME%REACTOME%REACT\_147857.2 | -2.11 | 0 | 8.67E-04 |
| SINGLE-ORGANISM MEMBRANE BUDDING%GOBP%GO:1902591 | -2.10 | 0 | 9.21E-04 |
| REGULATION OF CHOLESTEROL BIOSYNTHESIS BY SREBP (SREBF)%REACTOME%REACT\_147797.3 | -2.10 | 0 | 9.23E-04 |
| NEUTRAL LIPID METABOLIC PROCESS%GOBP%GO:0006638 | -2.10 | 0 | 9.52E-04 |
| BINDING AND UPTAKE OF LIGANDS BY SCAVENGER RECEPTORS%REACTOME%REACT\_160300.3 | -2.10 | 0 | 9.68E-04 |
| TRIGLYCERIDE METABOLIC PROCESS%GOBP%GO:0006641 | -2.10 | 0 | 9.98E-04 |
| VESICLE COATING%GOBP%GO:0006901 | -2.10 | 0 | 0.00100911 |
| MPS IX - NATOWICZ SYNDROME%REACTOME%REACT\_147739.2 | -2.09 | 0 | 0.00116699 |
| MPS IIIB - SANFILIPPO SYNDROME B%REACTOME%REACT\_147788.2 | -2.09 | 0 | 0.0011787 |
| MUCOPOLYSACCHARIDOSES%REACTOME%REACT\_147853.2 | -2.09 | 0 | 0.0011918 |
| AXON GUIDANCE%REACTOME%REACT\_18266.1 | -2.09 | 0 | 0.00121627 |
| GLYCEROLIPID METABOLISM%KEGG%HSA00561 | -2.09 | 0 | 0.00124007 |
| KERATAN SULFATE BIOSYNTHESIS%REACTOME%REACT\_121120.2 | -2.08 | 0 | 0.00127631 |
| REGULATION OF ACTIN CYTOSKELETON%KEGG%HSA04810 | -2.08 | 0 | 0.00128702 |
| NEURON PROJECTION EXTENSION%GOBP%GO:1990138 | -2.08 | 0 | 0.00141312 |
| UROKINASE-TYPE PLASMINOGEN ACTIVATOR (UPA) AND UPAR-MEDIATED SIGNALING%PATHWAY INTERACTION DATABASE NCI-NATURE CURATED DATA%UROKINASE-TYPE PLASMINOGEN ACTIVATOR (UPA) AND UPAR-MEDIATED SIGNALING | -2.08 | 0 | 0.00141596 |
| BIOCARTA\_AMI\_PATHWAY%MSIGDB\_C2%BIOCARTA\_AMI\_PATHWAY | -2.07 | 0 | 0.00144532 |
| KERATAN SULFATE BIOSYNTHETIC PROCESS%GOBP%GO:0018146 | -2.07 | 0 | 0.00151327 |

## Slide 3
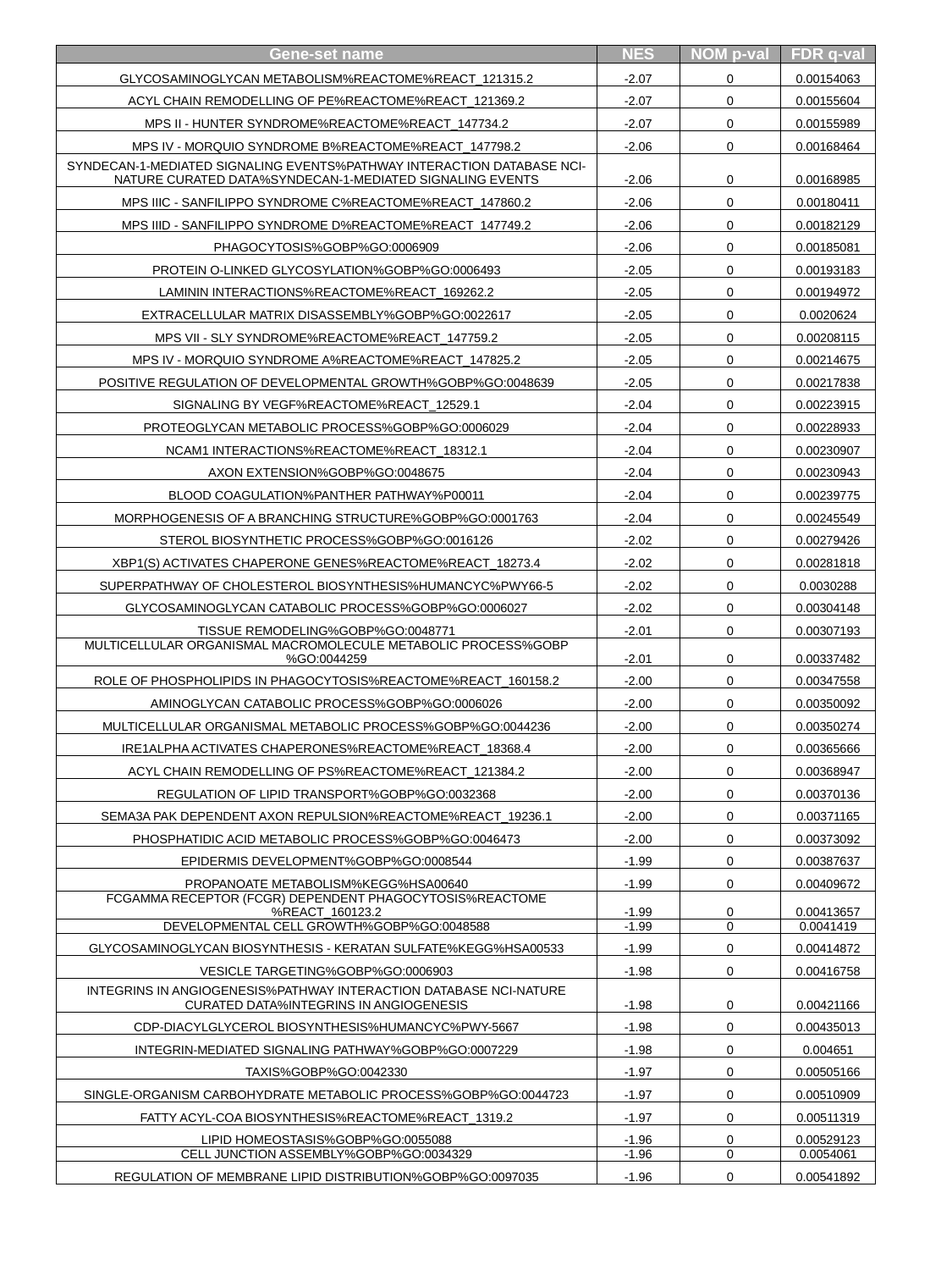

| Gene-set name | NES | NOM p-val | FDR q-val |
| --- | --- | --- | --- |
| GLYCOSAMINOGLYCAN METABOLISM%REACTOME%REACT\_121315.2 | -2.07 | 0 | 0.00154063 |
| ACYL CHAIN REMODELLING OF PE%REACTOME%REACT\_121369.2 | -2.07 | 0 | 0.00155604 |
| MPS II - HUNTER SYNDROME%REACTOME%REACT\_147734.2 | -2.07 | 0 | 0.00155989 |
| MPS IV - MORQUIO SYNDROME B%REACTOME%REACT\_147798.2 | -2.06 | 0 | 0.00168464 |
| SYNDECAN-1-MEDIATED SIGNALING EVENTS%PATHWAY INTERACTION DATABASE NCI-NATURE CURATED DATA%SYNDECAN-1-MEDIATED SIGNALING EVENTS | -2.06 | 0 | 0.00168985 |
| MPS IIIC - SANFILIPPO SYNDROME C%REACTOME%REACT\_147860.2 | -2.06 | 0 | 0.00180411 |
| MPS IIID - SANFILIPPO SYNDROME D%REACTOME%REACT\_147749.2 | -2.06 | 0 | 0.00182129 |
| PHAGOCYTOSIS%GOBP%GO:0006909 | -2.06 | 0 | 0.00185081 |
| PROTEIN O-LINKED GLYCOSYLATION%GOBP%GO:0006493 | -2.05 | 0 | 0.00193183 |
| LAMININ INTERACTIONS%REACTOME%REACT\_169262.2 | -2.05 | 0 | 0.00194972 |
| EXTRACELLULAR MATRIX DISASSEMBLY%GOBP%GO:0022617 | -2.05 | 0 | 0.0020624 |
| MPS VII - SLY SYNDROME%REACTOME%REACT\_147759.2 | -2.05 | 0 | 0.00208115 |
| MPS IV - MORQUIO SYNDROME A%REACTOME%REACT\_147825.2 | -2.05 | 0 | 0.00214675 |
| POSITIVE REGULATION OF DEVELOPMENTAL GROWTH%GOBP%GO:0048639 | -2.05 | 0 | 0.00217838 |
| SIGNALING BY VEGF%REACTOME%REACT\_12529.1 | -2.04 | 0 | 0.00223915 |
| PROTEOGLYCAN METABOLIC PROCESS%GOBP%GO:0006029 | -2.04 | 0 | 0.00228933 |
| NCAM1 INTERACTIONS%REACTOME%REACT\_18312.1 | -2.04 | 0 | 0.00230907 |
| AXON EXTENSION%GOBP%GO:0048675 | -2.04 | 0 | 0.00230943 |
| BLOOD COAGULATION%PANTHER PATHWAY%P00011 | -2.04 | 0 | 0.00239775 |
| MORPHOGENESIS OF A BRANCHING STRUCTURE%GOBP%GO:0001763 | -2.04 | 0 | 0.00245549 |
| STEROL BIOSYNTHETIC PROCESS%GOBP%GO:0016126 | -2.02 | 0 | 0.00279426 |
| XBP1(S) ACTIVATES CHAPERONE GENES%REACTOME%REACT\_18273.4 | -2.02 | 0 | 0.00281818 |
| SUPERPATHWAY OF CHOLESTEROL BIOSYNTHESIS%HUMANCYC%PWY66-5 | -2.02 | 0 | 0.0030288 |
| GLYCOSAMINOGLYCAN CATABOLIC PROCESS%GOBP%GO:0006027 | -2.02 | 0 | 0.00304148 |
| TISSUE REMODELING%GOBP%GO:0048771 | -2.01 | 0 | 0.00307193 |
| MULTICELLULAR ORGANISMAL MACROMOLECULE METABOLIC PROCESS%GOBP%GO:0044259 | -2.01 | 0 | 0.00337482 |
| ROLE OF PHOSPHOLIPIDS IN PHAGOCYTOSIS%REACTOME%REACT\_160158.2 | -2.00 | 0 | 0.00347558 |
| AMINOGLYCAN CATABOLIC PROCESS%GOBP%GO:0006026 | -2.00 | 0 | 0.00350092 |
| MULTICELLULAR ORGANISMAL METABOLIC PROCESS%GOBP%GO:0044236 | -2.00 | 0 | 0.00350274 |
| IRE1ALPHA ACTIVATES CHAPERONES%REACTOME%REACT\_18368.4 | -2.00 | 0 | 0.00365666 |
| ACYL CHAIN REMODELLING OF PS%REACTOME%REACT\_121384.2 | -2.00 | 0 | 0.00368947 |
| REGULATION OF LIPID TRANSPORT%GOBP%GO:0032368 | -2.00 | 0 | 0.00370136 |
| SEMA3A PAK DEPENDENT AXON REPULSION%REACTOME%REACT\_19236.1 | -2.00 | 0 | 0.00371165 |
| PHOSPHATIDIC ACID METABOLIC PROCESS%GOBP%GO:0046473 | -2.00 | 0 | 0.00373092 |
| EPIDERMIS DEVELOPMENT%GOBP%GO:0008544 | -1.99 | 0 | 0.00387637 |
| PROPANOATE METABOLISM%KEGG%HSA00640 | -1.99 | 0 | 0.00409672 |
| FCGAMMA RECEPTOR (FCGR) DEPENDENT PHAGOCYTOSIS%REACTOME%REACT\_160123.2 | -1.99 | 0 | 0.00413657 |
| DEVELOPMENTAL CELL GROWTH%GOBP%GO:0048588 | -1.99 | 0 | 0.0041419 |
| GLYCOSAMINOGLYCAN BIOSYNTHESIS - KERATAN SULFATE%KEGG%HSA00533 | -1.99 | 0 | 0.00414872 |
| VESICLE TARGETING%GOBP%GO:0006903 | -1.98 | 0 | 0.00416758 |
| INTEGRINS IN ANGIOGENESIS%PATHWAY INTERACTION DATABASE NCI-NATURE CURATED DATA%INTEGRINS IN ANGIOGENESIS | -1.98 | 0 | 0.00421166 |
| CDP-DIACYLGLYCEROL BIOSYNTHESIS%HUMANCYC%PWY-5667 | -1.98 | 0 | 0.00435013 |
| INTEGRIN-MEDIATED SIGNALING PATHWAY%GOBP%GO:0007229 | -1.98 | 0 | 0.004651 |
| TAXIS%GOBP%GO:0042330 | -1.97 | 0 | 0.00505166 |
| SINGLE-ORGANISM CARBOHYDRATE METABOLIC PROCESS%GOBP%GO:0044723 | -1.97 | 0 | 0.00510909 |
| FATTY ACYL-COA BIOSYNTHESIS%REACTOME%REACT\_1319.2 | -1.97 | 0 | 0.00511319 |
| LIPID HOMEOSTASIS%GOBP%GO:0055088 | -1.96 | 0 | 0.00529123 |
| CELL JUNCTION ASSEMBLY%GOBP%GO:0034329 | -1.96 | 0 | 0.0054061 |
| REGULATION OF MEMBRANE LIPID DISTRIBUTION%GOBP%GO:0097035 | -1.96 | 0 | 0.00541892 |

## Slide 4
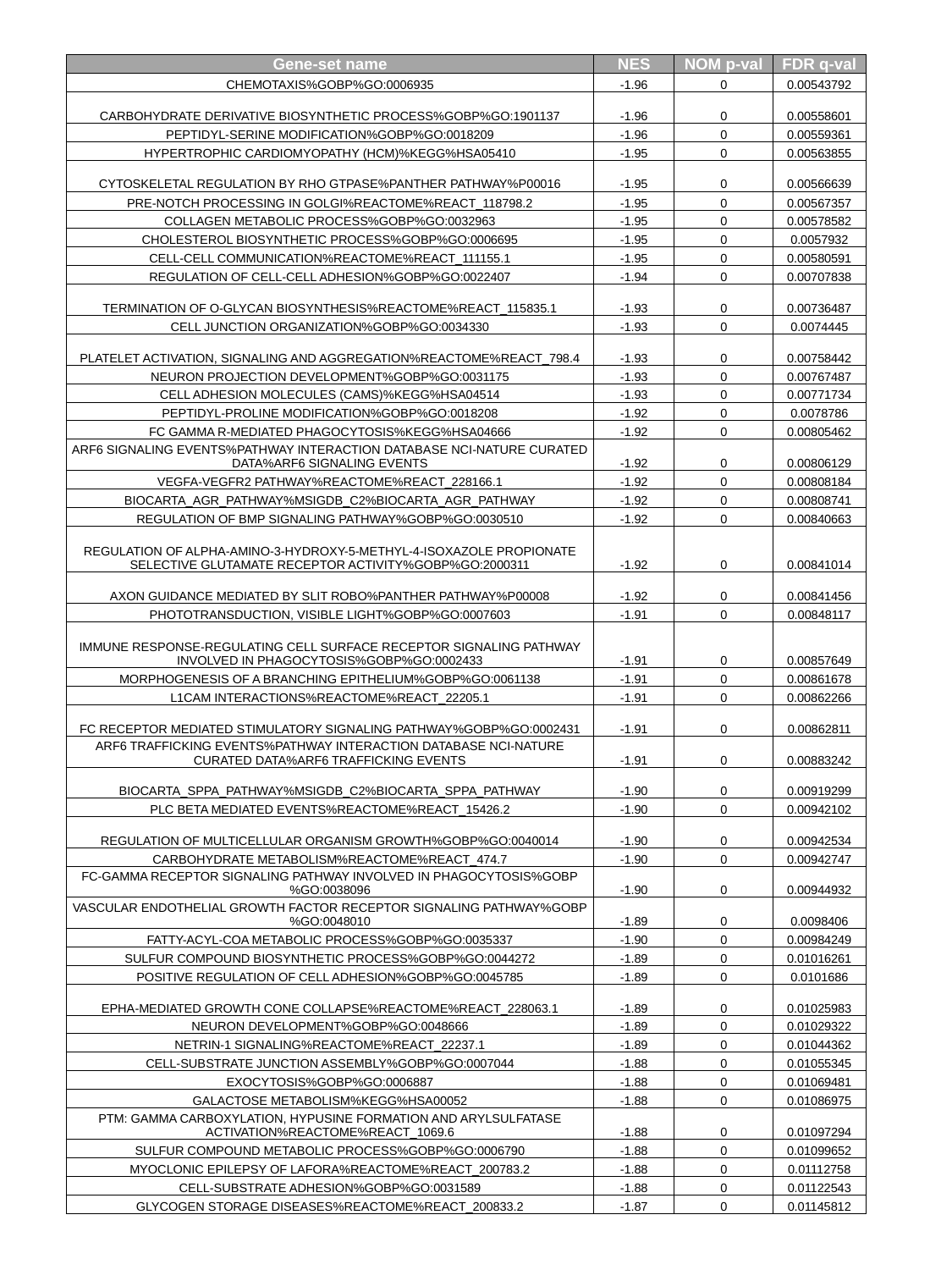

| Gene-set name | NES | NOM p-val | FDR q-val |
| --- | --- | --- | --- |
| CHEMOTAXIS%GOBP%GO:0006935 | -1.96 | 0 | 0.00543792 |
| CARBOHYDRATE DERIVATIVE BIOSYNTHETIC PROCESS%GOBP%GO:1901137 | -1.96 | 0 | 0.00558601 |
| PEPTIDYL-SERINE MODIFICATION%GOBP%GO:0018209 | -1.96 | 0 | 0.00559361 |
| HYPERTROPHIC CARDIOMYOPATHY (HCM)%KEGG%HSA05410 | -1.95 | 0 | 0.00563855 |
| CYTOSKELETAL REGULATION BY RHO GTPASE%PANTHER PATHWAY%P00016 | -1.95 | 0 | 0.00566639 |
| PRE-NOTCH PROCESSING IN GOLGI%REACTOME%REACT\_118798.2 | -1.95 | 0 | 0.00567357 |
| COLLAGEN METABOLIC PROCESS%GOBP%GO:0032963 | -1.95 | 0 | 0.00578582 |
| CHOLESTEROL BIOSYNTHETIC PROCESS%GOBP%GO:0006695 | -1.95 | 0 | 0.0057932 |
| CELL-CELL COMMUNICATION%REACTOME%REACT\_111155.1 | -1.95 | 0 | 0.00580591 |
| REGULATION OF CELL-CELL ADHESION%GOBP%GO:0022407 | -1.94 | 0 | 0.00707838 |
| TERMINATION OF O-GLYCAN BIOSYNTHESIS%REACTOME%REACT\_115835.1 | -1.93 | 0 | 0.00736487 |
| CELL JUNCTION ORGANIZATION%GOBP%GO:0034330 | -1.93 | 0 | 0.0074445 |
| PLATELET ACTIVATION, SIGNALING AND AGGREGATION%REACTOME%REACT\_798.4 | -1.93 | 0 | 0.00758442 |
| NEURON PROJECTION DEVELOPMENT%GOBP%GO:0031175 | -1.93 | 0 | 0.00767487 |
| CELL ADHESION MOLECULES (CAMS)%KEGG%HSA04514 | -1.93 | 0 | 0.00771734 |
| PEPTIDYL-PROLINE MODIFICATION%GOBP%GO:0018208 | -1.92 | 0 | 0.0078786 |
| FC GAMMA R-MEDIATED PHAGOCYTOSIS%KEGG%HSA04666 | -1.92 | 0 | 0.00805462 |
| ARF6 SIGNALING EVENTS%PATHWAY INTERACTION DATABASE NCI-NATURE CURATED DATA%ARF6 SIGNALING EVENTS | -1.92 | 0 | 0.00806129 |
| VEGFA-VEGFR2 PATHWAY%REACTOME%REACT\_228166.1 | -1.92 | 0 | 0.00808184 |
| BIOCARTA\_AGR\_PATHWAY%MSIGDB\_C2%BIOCARTA\_AGR\_PATHWAY | -1.92 | 0 | 0.00808741 |
| REGULATION OF BMP SIGNALING PATHWAY%GOBP%GO:0030510 | -1.92 | 0 | 0.00840663 |
| REGULATION OF ALPHA-AMINO-3-HYDROXY-5-METHYL-4-ISOXAZOLE PROPIONATE SELECTIVE GLUTAMATE RECEPTOR ACTIVITY%GOBP%GO:2000311 | -1.92 | 0 | 0.00841014 |
| AXON GUIDANCE MEDIATED BY SLIT ROBO%PANTHER PATHWAY%P00008 | -1.92 | 0 | 0.00841456 |
| PHOTOTRANSDUCTION, VISIBLE LIGHT%GOBP%GO:0007603 | -1.91 | 0 | 0.00848117 |
| IMMUNE RESPONSE-REGULATING CELL SURFACE RECEPTOR SIGNALING PATHWAY INVOLVED IN PHAGOCYTOSIS%GOBP%GO:0002433 | -1.91 | 0 | 0.00857649 |
| MORPHOGENESIS OF A BRANCHING EPITHELIUM%GOBP%GO:0061138 | -1.91 | 0 | 0.00861678 |
| L1CAM INTERACTIONS%REACTOME%REACT\_22205.1 | -1.91 | 0 | 0.00862266 |
| FC RECEPTOR MEDIATED STIMULATORY SIGNALING PATHWAY%GOBP%GO:0002431 | -1.91 | 0 | 0.00862811 |
| ARF6 TRAFFICKING EVENTS%PATHWAY INTERACTION DATABASE NCI-NATURE CURATED DATA%ARF6 TRAFFICKING EVENTS | -1.91 | 0 | 0.00883242 |
| BIOCARTA\_SPPA\_PATHWAY%MSIGDB\_C2%BIOCARTA\_SPPA\_PATHWAY | -1.90 | 0 | 0.00919299 |
| PLC BETA MEDIATED EVENTS%REACTOME%REACT\_15426.2 | -1.90 | 0 | 0.00942102 |
| REGULATION OF MULTICELLULAR ORGANISM GROWTH%GOBP%GO:0040014 | -1.90 | 0 | 0.00942534 |
| CARBOHYDRATE METABOLISM%REACTOME%REACT\_474.7 | -1.90 | 0 | 0.00942747 |
| FC-GAMMA RECEPTOR SIGNALING PATHWAY INVOLVED IN PHAGOCYTOSIS%GOBP%GO:0038096 | -1.90 | 0 | 0.00944932 |
| VASCULAR ENDOTHELIAL GROWTH FACTOR RECEPTOR SIGNALING PATHWAY%GOBP%GO:0048010 | -1.89 | 0 | 0.0098406 |
| FATTY-ACYL-COA METABOLIC PROCESS%GOBP%GO:0035337 | -1.90 | 0 | 0.00984249 |
| SULFUR COMPOUND BIOSYNTHETIC PROCESS%GOBP%GO:0044272 | -1.89 | 0 | 0.01016261 |
| POSITIVE REGULATION OF CELL ADHESION%GOBP%GO:0045785 | -1.89 | 0 | 0.0101686 |
| EPHA-MEDIATED GROWTH CONE COLLAPSE%REACTOME%REACT\_228063.1 | -1.89 | 0 | 0.01025983 |
| NEURON DEVELOPMENT%GOBP%GO:0048666 | -1.89 | 0 | 0.01029322 |
| NETRIN-1 SIGNALING%REACTOME%REACT\_22237.1 | -1.89 | 0 | 0.01044362 |
| CELL-SUBSTRATE JUNCTION ASSEMBLY%GOBP%GO:0007044 | -1.88 | 0 | 0.01055345 |
| EXOCYTOSIS%GOBP%GO:0006887 | -1.88 | 0 | 0.01069481 |
| GALACTOSE METABOLISM%KEGG%HSA00052 | -1.88 | 0 | 0.01086975 |
| PTM: GAMMA CARBOXYLATION, HYPUSINE FORMATION AND ARYLSULFATASE ACTIVATION%REACTOME%REACT\_1069.6 | -1.88 | 0 | 0.01097294 |
| SULFUR COMPOUND METABOLIC PROCESS%GOBP%GO:0006790 | -1.88 | 0 | 0.01099652 |
| MYOCLONIC EPILEPSY OF LAFORA%REACTOME%REACT\_200783.2 | -1.88 | 0 | 0.01112758 |
| CELL-SUBSTRATE ADHESION%GOBP%GO:0031589 | -1.88 | 0 | 0.01122543 |
| GLYCOGEN STORAGE DISEASES%REACTOME%REACT\_200833.2 | -1.87 | 0 | 0.01145812 |

## Slide 5
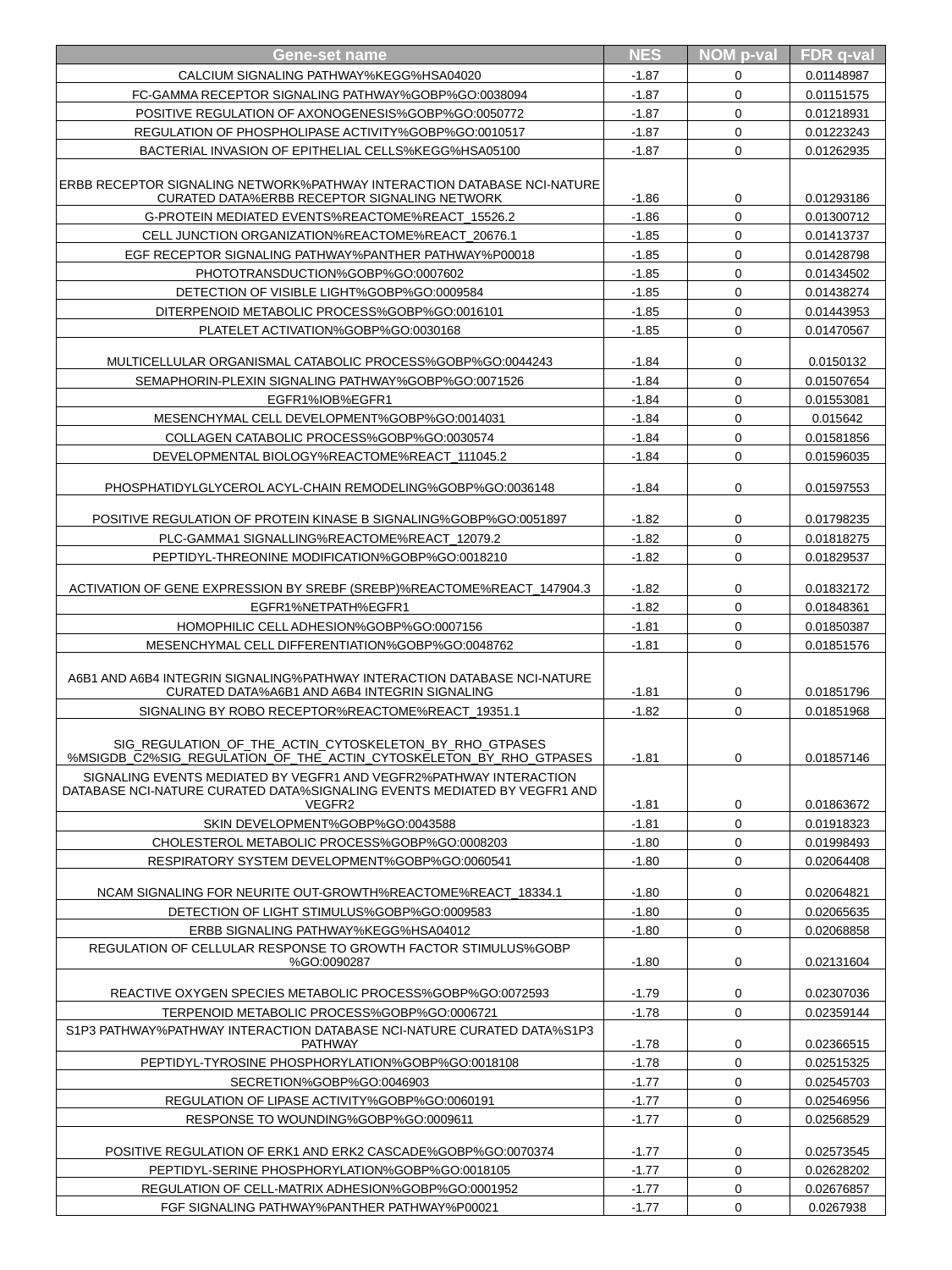

| Gene-set name | NES | NOM p-val | FDR q-val |
| --- | --- | --- | --- |
| CALCIUM SIGNALING PATHWAY%KEGG%HSA04020 | -1.87 | 0 | 0.01148987 |
| FC-GAMMA RECEPTOR SIGNALING PATHWAY%GOBP%GO:0038094 | -1.87 | 0 | 0.01151575 |
| POSITIVE REGULATION OF AXONOGENESIS%GOBP%GO:0050772 | -1.87 | 0 | 0.01218931 |
| REGULATION OF PHOSPHOLIPASE ACTIVITY%GOBP%GO:0010517 | -1.87 | 0 | 0.01223243 |
| BACTERIAL INVASION OF EPITHELIAL CELLS%KEGG%HSA05100 | -1.87 | 0 | 0.01262935 |
| ERBB RECEPTOR SIGNALING NETWORK%PATHWAY INTERACTION DATABASE NCI-NATURE CURATED DATA%ERBB RECEPTOR SIGNALING NETWORK | -1.86 | 0 | 0.01293186 |
| G-PROTEIN MEDIATED EVENTS%REACTOME%REACT\_15526.2 | -1.86 | 0 | 0.01300712 |
| CELL JUNCTION ORGANIZATION%REACTOME%REACT\_20676.1 | -1.85 | 0 | 0.01413737 |
| EGF RECEPTOR SIGNALING PATHWAY%PANTHER PATHWAY%P00018 | -1.85 | 0 | 0.01428798 |
| PHOTOTRANSDUCTION%GOBP%GO:0007602 | -1.85 | 0 | 0.01434502 |
| DETECTION OF VISIBLE LIGHT%GOBP%GO:0009584 | -1.85 | 0 | 0.01438274 |
| DITERPENOID METABOLIC PROCESS%GOBP%GO:0016101 | -1.85 | 0 | 0.01443953 |
| PLATELET ACTIVATION%GOBP%GO:0030168 | -1.85 | 0 | 0.01470567 |
| MULTICELLULAR ORGANISMAL CATABOLIC PROCESS%GOBP%GO:0044243 | -1.84 | 0 | 0.0150132 |
| SEMAPHORIN-PLEXIN SIGNALING PATHWAY%GOBP%GO:0071526 | -1.84 | 0 | 0.01507654 |
| EGFR1%IOB%EGFR1 | -1.84 | 0 | 0.01553081 |
| MESENCHYMAL CELL DEVELOPMENT%GOBP%GO:0014031 | -1.84 | 0 | 0.015642 |
| COLLAGEN CATABOLIC PROCESS%GOBP%GO:0030574 | -1.84 | 0 | 0.01581856 |
| DEVELOPMENTAL BIOLOGY%REACTOME%REACT\_111045.2 | -1.84 | 0 | 0.01596035 |
| PHOSPHATIDYLGLYCEROL ACYL-CHAIN REMODELING%GOBP%GO:0036148 | -1.84 | 0 | 0.01597553 |
| POSITIVE REGULATION OF PROTEIN KINASE B SIGNALING%GOBP%GO:0051897 | -1.82 | 0 | 0.01798235 |
| PLC-GAMMA1 SIGNALLING%REACTOME%REACT\_12079.2 | -1.82 | 0 | 0.01818275 |
| PEPTIDYL-THREONINE MODIFICATION%GOBP%GO:0018210 | -1.82 | 0 | 0.01829537 |
| ACTIVATION OF GENE EXPRESSION BY SREBF (SREBP)%REACTOME%REACT\_147904.3 | -1.82 | 0 | 0.01832172 |
| EGFR1%NETPATH%EGFR1 | -1.82 | 0 | 0.01848361 |
| HOMOPHILIC CELL ADHESION%GOBP%GO:0007156 | -1.81 | 0 | 0.01850387 |
| MESENCHYMAL CELL DIFFERENTIATION%GOBP%GO:0048762 | -1.81 | 0 | 0.01851576 |
| A6B1 AND A6B4 INTEGRIN SIGNALING%PATHWAY INTERACTION DATABASE NCI-NATURE CURATED DATA%A6B1 AND A6B4 INTEGRIN SIGNALING | -1.81 | 0 | 0.01851796 |
| SIGNALING BY ROBO RECEPTOR%REACTOME%REACT\_19351.1 | -1.82 | 0 | 0.01851968 |
| SIG\_REGULATION\_OF\_THE\_ACTIN\_CYTOSKELETON\_BY\_RHO\_GTPASES%MSIGDB\_C2%SIG\_REGULATION\_OF\_THE\_ACTIN\_CYTOSKELETON\_BY\_RHO\_GTPASES | -1.81 | 0 | 0.01857146 |
| SIGNALING EVENTS MEDIATED BY VEGFR1 AND VEGFR2%PATHWAY INTERACTION DATABASE NCI-NATURE CURATED DATA%SIGNALING EVENTS MEDIATED BY VEGFR1 AND VEGFR2 | -1.81 | 0 | 0.01863672 |
| SKIN DEVELOPMENT%GOBP%GO:0043588 | -1.81 | 0 | 0.01918323 |
| CHOLESTEROL METABOLIC PROCESS%GOBP%GO:0008203 | -1.80 | 0 | 0.01998493 |
| RESPIRATORY SYSTEM DEVELOPMENT%GOBP%GO:0060541 | -1.80 | 0 | 0.02064408 |
| NCAM SIGNALING FOR NEURITE OUT-GROWTH%REACTOME%REACT\_18334.1 | -1.80 | 0 | 0.02064821 |
| DETECTION OF LIGHT STIMULUS%GOBP%GO:0009583 | -1.80 | 0 | 0.02065635 |
| ERBB SIGNALING PATHWAY%KEGG%HSA04012 | -1.80 | 0 | 0.02068858 |
| REGULATION OF CELLULAR RESPONSE TO GROWTH FACTOR STIMULUS%GOBP%GO:0090287 | -1.80 | 0 | 0.02131604 |
| REACTIVE OXYGEN SPECIES METABOLIC PROCESS%GOBP%GO:0072593 | -1.79 | 0 | 0.02307036 |
| TERPENOID METABOLIC PROCESS%GOBP%GO:0006721 | -1.78 | 0 | 0.02359144 |
| S1P3 PATHWAY%PATHWAY INTERACTION DATABASE NCI-NATURE CURATED DATA%S1P3 PATHWAY | -1.78 | 0 | 0.02366515 |
| PEPTIDYL-TYROSINE PHOSPHORYLATION%GOBP%GO:0018108 | -1.78 | 0 | 0.02515325 |
| SECRETION%GOBP%GO:0046903 | -1.77 | 0 | 0.02545703 |
| REGULATION OF LIPASE ACTIVITY%GOBP%GO:0060191 | -1.77 | 0 | 0.02546956 |
| RESPONSE TO WOUNDING%GOBP%GO:0009611 | -1.77 | 0 | 0.02568529 |
| POSITIVE REGULATION OF ERK1 AND ERK2 CASCADE%GOBP%GO:0070374 | -1.77 | 0 | 0.02573545 |
| PEPTIDYL-SERINE PHOSPHORYLATION%GOBP%GO:0018105 | -1.77 | 0 | 0.02628202 |
| REGULATION OF CELL-MATRIX ADHESION%GOBP%GO:0001952 | -1.77 | 0 | 0.02676857 |
| FGF SIGNALING PATHWAY%PANTHER PATHWAY%P00021 | -1.77 | 0 | 0.0267938 |

## Slide 6
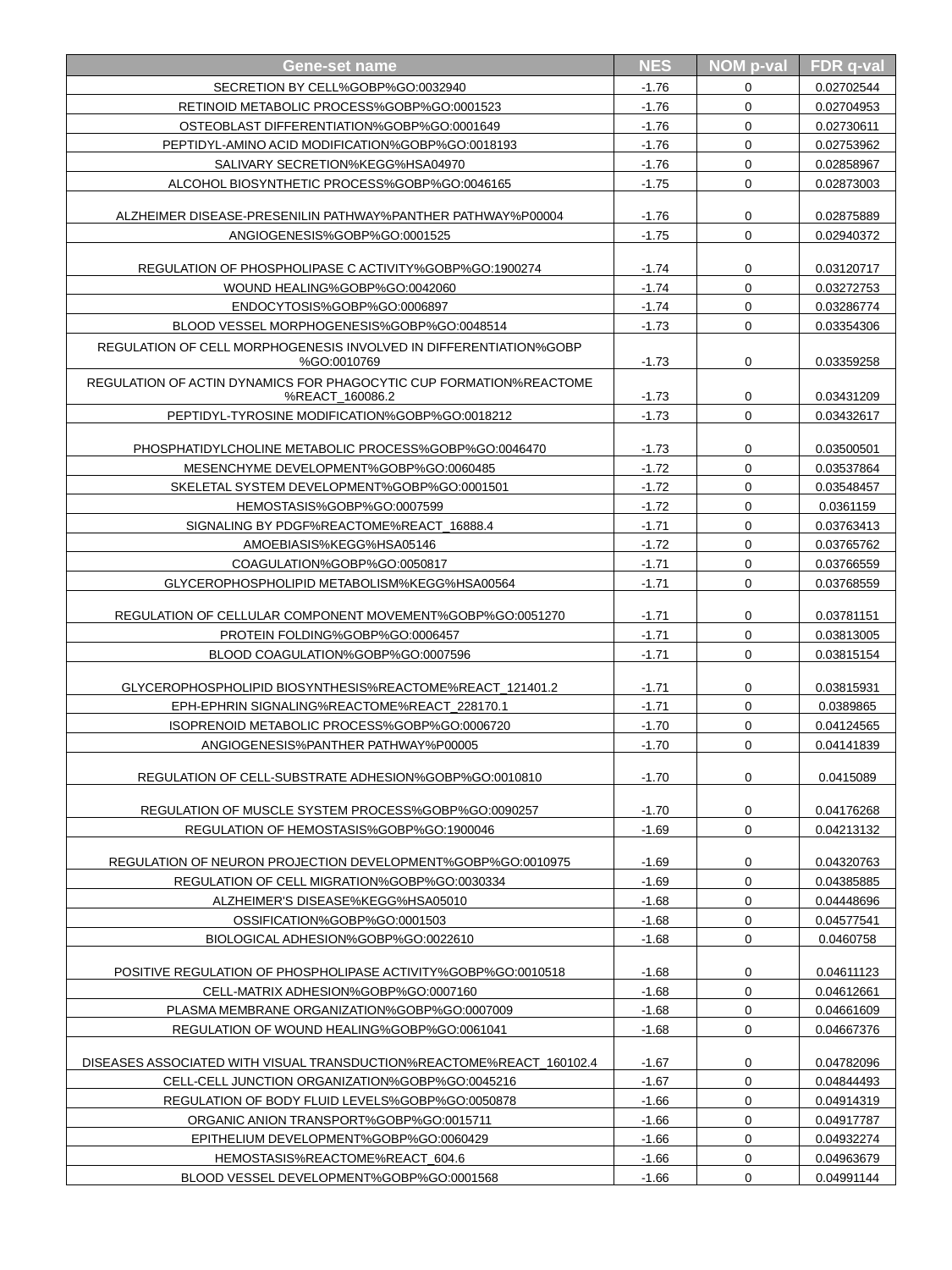

| Gene-set name | NES | NOM p-val | FDR q-val |
| --- | --- | --- | --- |
| SECRETION BY CELL%GOBP%GO:0032940 | -1.76 | 0 | 0.02702544 |
| RETINOID METABOLIC PROCESS%GOBP%GO:0001523 | -1.76 | 0 | 0.02704953 |
| OSTEOBLAST DIFFERENTIATION%GOBP%GO:0001649 | -1.76 | 0 | 0.02730611 |
| PEPTIDYL-AMINO ACID MODIFICATION%GOBP%GO:0018193 | -1.76 | 0 | 0.02753962 |
| SALIVARY SECRETION%KEGG%HSA04970 | -1.76 | 0 | 0.02858967 |
| ALCOHOL BIOSYNTHETIC PROCESS%GOBP%GO:0046165 | -1.75 | 0 | 0.02873003 |
| ALZHEIMER DISEASE-PRESENILIN PATHWAY%PANTHER PATHWAY%P00004 | -1.76 | 0 | 0.02875889 |
| ANGIOGENESIS%GOBP%GO:0001525 | -1.75 | 0 | 0.02940372 |
| REGULATION OF PHOSPHOLIPASE C ACTIVITY%GOBP%GO:1900274 | -1.74 | 0 | 0.03120717 |
| WOUND HEALING%GOBP%GO:0042060 | -1.74 | 0 | 0.03272753 |
| ENDOCYTOSIS%GOBP%GO:0006897 | -1.74 | 0 | 0.03286774 |
| BLOOD VESSEL MORPHOGENESIS%GOBP%GO:0048514 | -1.73 | 0 | 0.03354306 |
| REGULATION OF CELL MORPHOGENESIS INVOLVED IN DIFFERENTIATION%GOBP%GO:0010769 | -1.73 | 0 | 0.03359258 |
| REGULATION OF ACTIN DYNAMICS FOR PHAGOCYTIC CUP FORMATION%REACTOME%REACT\_160086.2 | -1.73 | 0 | 0.03431209 |
| PEPTIDYL-TYROSINE MODIFICATION%GOBP%GO:0018212 | -1.73 | 0 | 0.03432617 |
| PHOSPHATIDYLCHOLINE METABOLIC PROCESS%GOBP%GO:0046470 | -1.73 | 0 | 0.03500501 |
| MESENCHYME DEVELOPMENT%GOBP%GO:0060485 | -1.72 | 0 | 0.03537864 |
| SKELETAL SYSTEM DEVELOPMENT%GOBP%GO:0001501 | -1.72 | 0 | 0.03548457 |
| HEMOSTASIS%GOBP%GO:0007599 | -1.72 | 0 | 0.0361159 |
| SIGNALING BY PDGF%REACTOME%REACT\_16888.4 | -1.71 | 0 | 0.03763413 |
| AMOEBIASIS%KEGG%HSA05146 | -1.72 | 0 | 0.03765762 |
| COAGULATION%GOBP%GO:0050817 | -1.71 | 0 | 0.03766559 |
| GLYCEROPHOSPHOLIPID METABOLISM%KEGG%HSA00564 | -1.71 | 0 | 0.03768559 |
| REGULATION OF CELLULAR COMPONENT MOVEMENT%GOBP%GO:0051270 | -1.71 | 0 | 0.03781151 |
| PROTEIN FOLDING%GOBP%GO:0006457 | -1.71 | 0 | 0.03813005 |
| BLOOD COAGULATION%GOBP%GO:0007596 | -1.71 | 0 | 0.03815154 |
| GLYCEROPHOSPHOLIPID BIOSYNTHESIS%REACTOME%REACT\_121401.2 | -1.71 | 0 | 0.03815931 |
| EPH-EPHRIN SIGNALING%REACTOME%REACT\_228170.1 | -1.71 | 0 | 0.0389865 |
| ISOPRENOID METABOLIC PROCESS%GOBP%GO:0006720 | -1.70 | 0 | 0.04124565 |
| ANGIOGENESIS%PANTHER PATHWAY%P00005 | -1.70 | 0 | 0.04141839 |
| REGULATION OF CELL-SUBSTRATE ADHESION%GOBP%GO:0010810 | -1.70 | 0 | 0.0415089 |
| REGULATION OF MUSCLE SYSTEM PROCESS%GOBP%GO:0090257 | -1.70 | 0 | 0.04176268 |
| REGULATION OF HEMOSTASIS%GOBP%GO:1900046 | -1.69 | 0 | 0.04213132 |
| REGULATION OF NEURON PROJECTION DEVELOPMENT%GOBP%GO:0010975 | -1.69 | 0 | 0.04320763 |
| REGULATION OF CELL MIGRATION%GOBP%GO:0030334 | -1.69 | 0 | 0.04385885 |
| ALZHEIMER'S DISEASE%KEGG%HSA05010 | -1.68 | 0 | 0.04448696 |
| OSSIFICATION%GOBP%GO:0001503 | -1.68 | 0 | 0.04577541 |
| BIOLOGICAL ADHESION%GOBP%GO:0022610 | -1.68 | 0 | 0.0460758 |
| POSITIVE REGULATION OF PHOSPHOLIPASE ACTIVITY%GOBP%GO:0010518 | -1.68 | 0 | 0.04611123 |
| CELL-MATRIX ADHESION%GOBP%GO:0007160 | -1.68 | 0 | 0.04612661 |
| PLASMA MEMBRANE ORGANIZATION%GOBP%GO:0007009 | -1.68 | 0 | 0.04661609 |
| REGULATION OF WOUND HEALING%GOBP%GO:0061041 | -1.68 | 0 | 0.04667376 |
| DISEASES ASSOCIATED WITH VISUAL TRANSDUCTION%REACTOME%REACT\_160102.4 | -1.67 | 0 | 0.04782096 |
| CELL-CELL JUNCTION ORGANIZATION%GOBP%GO:0045216 | -1.67 | 0 | 0.04844493 |
| REGULATION OF BODY FLUID LEVELS%GOBP%GO:0050878 | -1.66 | 0 | 0.04914319 |
| ORGANIC ANION TRANSPORT%GOBP%GO:0015711 | -1.66 | 0 | 0.04917787 |
| EPITHELIUM DEVELOPMENT%GOBP%GO:0060429 | -1.66 | 0 | 0.04932274 |
| HEMOSTASIS%REACTOME%REACT\_604.6 | -1.66 | 0 | 0.04963679 |
| BLOOD VESSEL DEVELOPMENT%GOBP%GO:0001568 | -1.66 | 0 | 0.04991144 |

## Slide 7
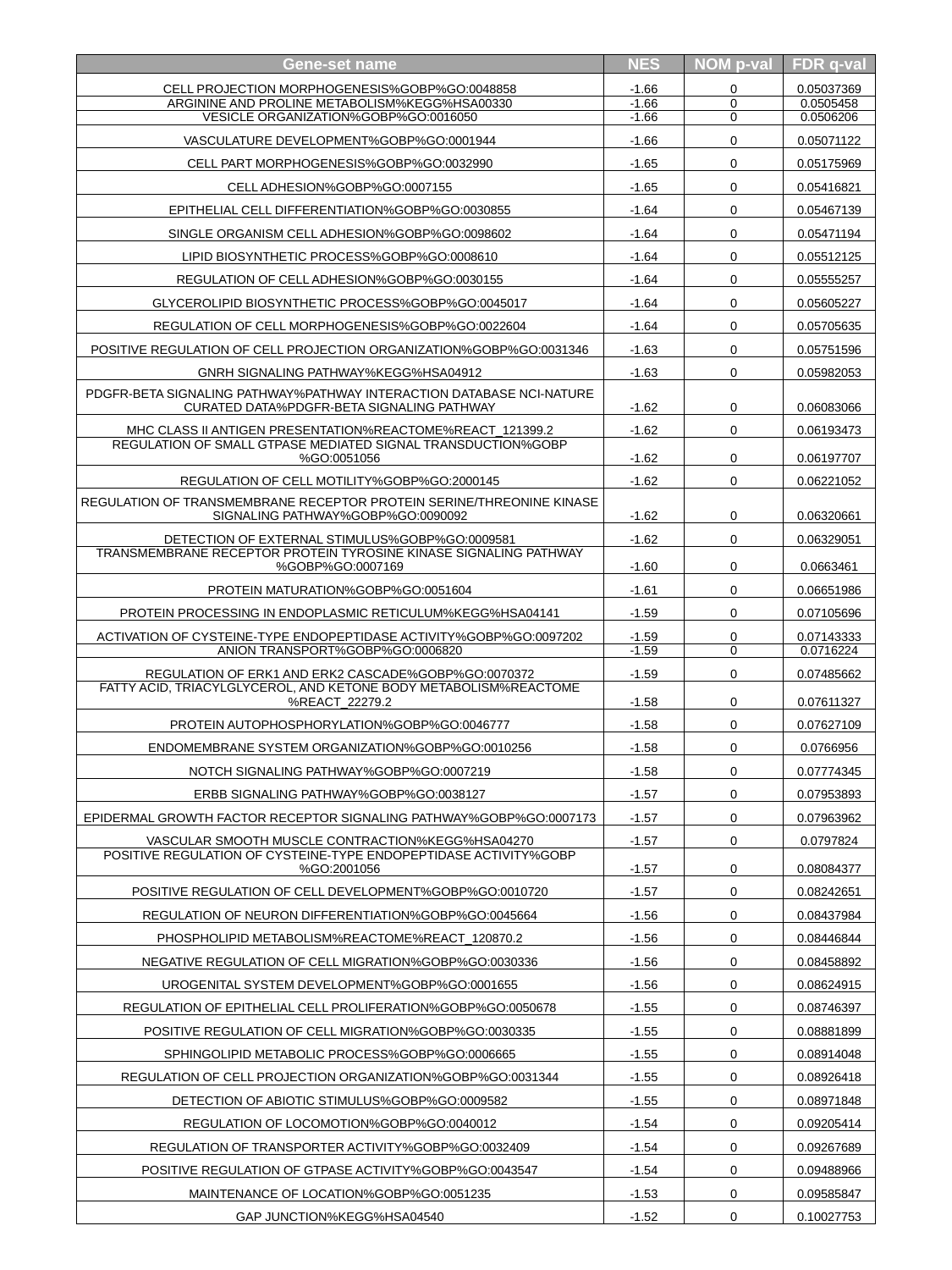

| Gene-set name | NES | NOM p-val | FDR q-val |
| --- | --- | --- | --- |
| CELL PROJECTION MORPHOGENESIS%GOBP%GO:0048858 | -1.66 | 0 | 0.05037369 |
| ARGININE AND PROLINE METABOLISM%KEGG%HSA00330 | -1.66 | 0 | 0.0505458 |
| VESICLE ORGANIZATION%GOBP%GO:0016050 | -1.66 | 0 | 0.0506206 |
| VASCULATURE DEVELOPMENT%GOBP%GO:0001944 | -1.66 | 0 | 0.05071122 |
| CELL PART MORPHOGENESIS%GOBP%GO:0032990 | -1.65 | 0 | 0.05175969 |
| CELL ADHESION%GOBP%GO:0007155 | -1.65 | 0 | 0.05416821 |
| EPITHELIAL CELL DIFFERENTIATION%GOBP%GO:0030855 | -1.64 | 0 | 0.05467139 |
| SINGLE ORGANISM CELL ADHESION%GOBP%GO:0098602 | -1.64 | 0 | 0.05471194 |
| LIPID BIOSYNTHETIC PROCESS%GOBP%GO:0008610 | -1.64 | 0 | 0.05512125 |
| REGULATION OF CELL ADHESION%GOBP%GO:0030155 | -1.64 | 0 | 0.05555257 |
| GLYCEROLIPID BIOSYNTHETIC PROCESS%GOBP%GO:0045017 | -1.64 | 0 | 0.05605227 |
| REGULATION OF CELL MORPHOGENESIS%GOBP%GO:0022604 | -1.64 | 0 | 0.05705635 |
| POSITIVE REGULATION OF CELL PROJECTION ORGANIZATION%GOBP%GO:0031346 | -1.63 | 0 | 0.05751596 |
| GNRH SIGNALING PATHWAY%KEGG%HSA04912 | -1.63 | 0 | 0.05982053 |
| PDGFR-BETA SIGNALING PATHWAY%PATHWAY INTERACTION DATABASE NCI-NATURE CURATED DATA%PDGFR-BETA SIGNALING PATHWAY | -1.62 | 0 | 0.06083066 |
| MHC CLASS II ANTIGEN PRESENTATION%REACTOME%REACT\_121399.2 | -1.62 | 0 | 0.06193473 |
| REGULATION OF SMALL GTPASE MEDIATED SIGNAL TRANSDUCTION%GOBP%GO:0051056 | -1.62 | 0 | 0.06197707 |
| REGULATION OF CELL MOTILITY%GOBP%GO:2000145 | -1.62 | 0 | 0.06221052 |
| REGULATION OF TRANSMEMBRANE RECEPTOR PROTEIN SERINE/THREONINE KINASE SIGNALING PATHWAY%GOBP%GO:0090092 | -1.62 | 0 | 0.06320661 |
| DETECTION OF EXTERNAL STIMULUS%GOBP%GO:0009581 | -1.62 | 0 | 0.06329051 |
| TRANSMEMBRANE RECEPTOR PROTEIN TYROSINE KINASE SIGNALING PATHWAY%GOBP%GO:0007169 | -1.60 | 0 | 0.0663461 |
| PROTEIN MATURATION%GOBP%GO:0051604 | -1.61 | 0 | 0.06651986 |
| PROTEIN PROCESSING IN ENDOPLASMIC RETICULUM%KEGG%HSA04141 | -1.59 | 0 | 0.07105696 |
| ACTIVATION OF CYSTEINE-TYPE ENDOPEPTIDASE ACTIVITY%GOBP%GO:0097202 | -1.59 | 0 | 0.07143333 |
| ANION TRANSPORT%GOBP%GO:0006820 | -1.59 | 0 | 0.0716224 |
| REGULATION OF ERK1 AND ERK2 CASCADE%GOBP%GO:0070372 | -1.59 | 0 | 0.07485662 |
| FATTY ACID, TRIACYLGLYCEROL, AND KETONE BODY METABOLISM%REACTOME%REACT\_22279.2 | -1.58 | 0 | 0.07611327 |
| PROTEIN AUTOPHOSPHORYLATION%GOBP%GO:0046777 | -1.58 | 0 | 0.07627109 |
| ENDOMEMBRANE SYSTEM ORGANIZATION%GOBP%GO:0010256 | -1.58 | 0 | 0.0766956 |
| NOTCH SIGNALING PATHWAY%GOBP%GO:0007219 | -1.58 | 0 | 0.07774345 |
| ERBB SIGNALING PATHWAY%GOBP%GO:0038127 | -1.57 | 0 | 0.07953893 |
| EPIDERMAL GROWTH FACTOR RECEPTOR SIGNALING PATHWAY%GOBP%GO:0007173 | -1.57 | 0 | 0.07963962 |
| VASCULAR SMOOTH MUSCLE CONTRACTION%KEGG%HSA04270 | -1.57 | 0 | 0.0797824 |
| POSITIVE REGULATION OF CYSTEINE-TYPE ENDOPEPTIDASE ACTIVITY%GOBP%GO:2001056 | -1.57 | 0 | 0.08084377 |
| POSITIVE REGULATION OF CELL DEVELOPMENT%GOBP%GO:0010720 | -1.57 | 0 | 0.08242651 |
| REGULATION OF NEURON DIFFERENTIATION%GOBP%GO:0045664 | -1.56 | 0 | 0.08437984 |
| PHOSPHOLIPID METABOLISM%REACTOME%REACT\_120870.2 | -1.56 | 0 | 0.08446844 |
| NEGATIVE REGULATION OF CELL MIGRATION%GOBP%GO:0030336 | -1.56 | 0 | 0.08458892 |
| UROGENITAL SYSTEM DEVELOPMENT%GOBP%GO:0001655 | -1.56 | 0 | 0.08624915 |
| REGULATION OF EPITHELIAL CELL PROLIFERATION%GOBP%GO:0050678 | -1.55 | 0 | 0.08746397 |
| POSITIVE REGULATION OF CELL MIGRATION%GOBP%GO:0030335 | -1.55 | 0 | 0.08881899 |
| SPHINGOLIPID METABOLIC PROCESS%GOBP%GO:0006665 | -1.55 | 0 | 0.08914048 |
| REGULATION OF CELL PROJECTION ORGANIZATION%GOBP%GO:0031344 | -1.55 | 0 | 0.08926418 |
| DETECTION OF ABIOTIC STIMULUS%GOBP%GO:0009582 | -1.55 | 0 | 0.08971848 |
| REGULATION OF LOCOMOTION%GOBP%GO:0040012 | -1.54 | 0 | 0.09205414 |
| REGULATION OF TRANSPORTER ACTIVITY%GOBP%GO:0032409 | -1.54 | 0 | 0.09267689 |
| POSITIVE REGULATION OF GTPASE ACTIVITY%GOBP%GO:0043547 | -1.54 | 0 | 0.09488966 |
| MAINTENANCE OF LOCATION%GOBP%GO:0051235 | -1.53 | 0 | 0.09585847 |
| GAP JUNCTION%KEGG%HSA04540 | -1.52 | 0 | 0.10027753 |

## Slide 8
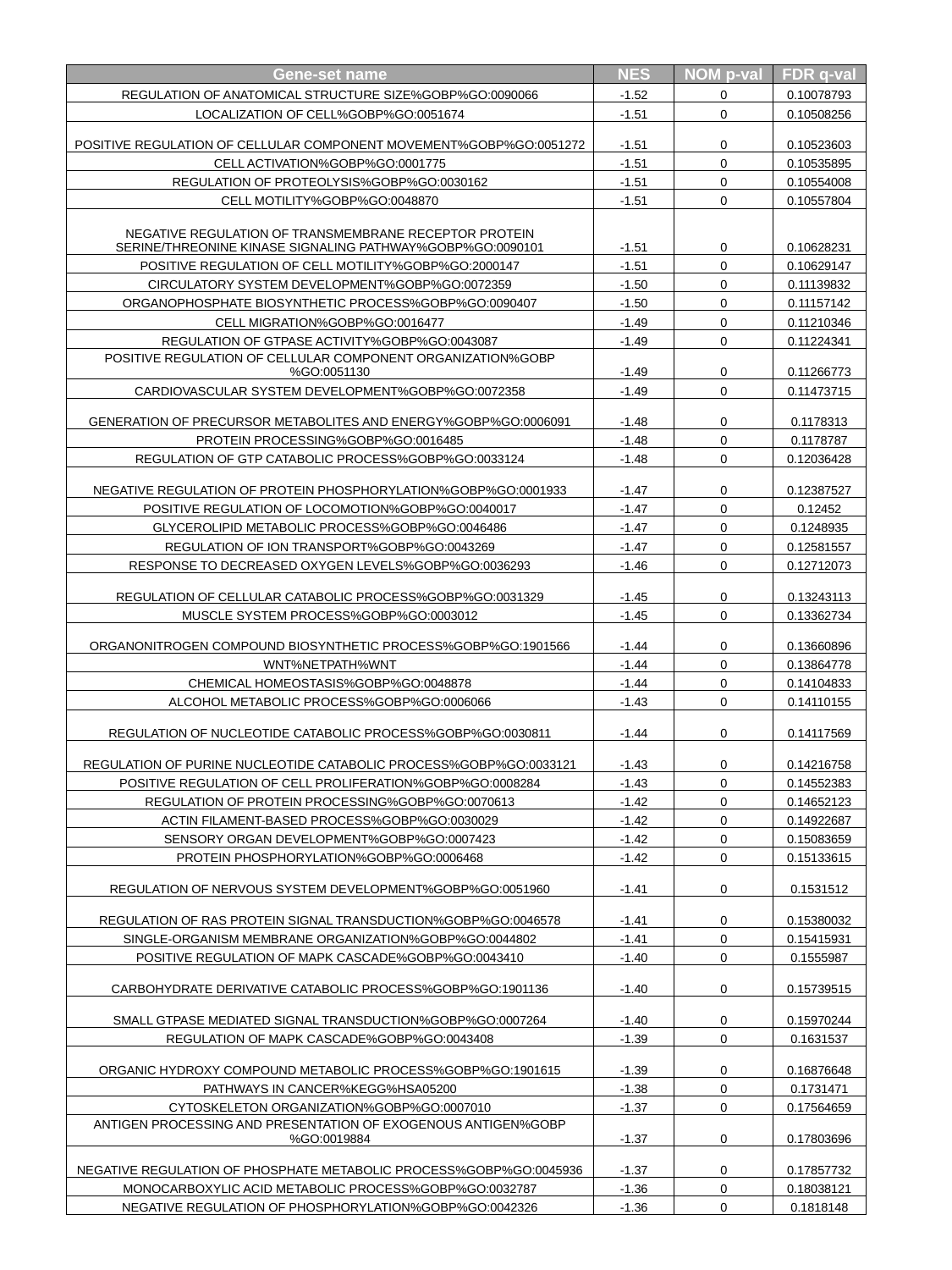

| Gene-set name | NES | NOM p-val | FDR q-val |
| --- | --- | --- | --- |
| REGULATION OF ANATOMICAL STRUCTURE SIZE%GOBP%GO:0090066 | -1.52 | 0 | 0.10078793 |
| LOCALIZATION OF CELL%GOBP%GO:0051674 | -1.51 | 0 | 0.10508256 |
| POSITIVE REGULATION OF CELLULAR COMPONENT MOVEMENT%GOBP%GO:0051272 | -1.51 | 0 | 0.10523603 |
| CELL ACTIVATION%GOBP%GO:0001775 | -1.51 | 0 | 0.10535895 |
| REGULATION OF PROTEOLYSIS%GOBP%GO:0030162 | -1.51 | 0 | 0.10554008 |
| CELL MOTILITY%GOBP%GO:0048870 | -1.51 | 0 | 0.10557804 |
| NEGATIVE REGULATION OF TRANSMEMBRANE RECEPTOR PROTEIN SERINE/THREONINE KINASE SIGNALING PATHWAY%GOBP%GO:0090101 | -1.51 | 0 | 0.10628231 |
| POSITIVE REGULATION OF CELL MOTILITY%GOBP%GO:2000147 | -1.51 | 0 | 0.10629147 |
| CIRCULATORY SYSTEM DEVELOPMENT%GOBP%GO:0072359 | -1.50 | 0 | 0.11139832 |
| ORGANOPHOSPHATE BIOSYNTHETIC PROCESS%GOBP%GO:0090407 | -1.50 | 0 | 0.11157142 |
| CELL MIGRATION%GOBP%GO:0016477 | -1.49 | 0 | 0.11210346 |
| REGULATION OF GTPASE ACTIVITY%GOBP%GO:0043087 | -1.49 | 0 | 0.11224341 |
| POSITIVE REGULATION OF CELLULAR COMPONENT ORGANIZATION%GOBP%GO:0051130 | -1.49 | 0 | 0.11266773 |
| CARDIOVASCULAR SYSTEM DEVELOPMENT%GOBP%GO:0072358 | -1.49 | 0 | 0.11473715 |
| GENERATION OF PRECURSOR METABOLITES AND ENERGY%GOBP%GO:0006091 | -1.48 | 0 | 0.1178313 |
| PROTEIN PROCESSING%GOBP%GO:0016485 | -1.48 | 0 | 0.1178787 |
| REGULATION OF GTP CATABOLIC PROCESS%GOBP%GO:0033124 | -1.48 | 0 | 0.12036428 |
| NEGATIVE REGULATION OF PROTEIN PHOSPHORYLATION%GOBP%GO:0001933 | -1.47 | 0 | 0.12387527 |
| POSITIVE REGULATION OF LOCOMOTION%GOBP%GO:0040017 | -1.47 | 0 | 0.12452 |
| GLYCEROLIPID METABOLIC PROCESS%GOBP%GO:0046486 | -1.47 | 0 | 0.1248935 |
| REGULATION OF ION TRANSPORT%GOBP%GO:0043269 | -1.47 | 0 | 0.12581557 |
| RESPONSE TO DECREASED OXYGEN LEVELS%GOBP%GO:0036293 | -1.46 | 0 | 0.12712073 |
| REGULATION OF CELLULAR CATABOLIC PROCESS%GOBP%GO:0031329 | -1.45 | 0 | 0.13243113 |
| MUSCLE SYSTEM PROCESS%GOBP%GO:0003012 | -1.45 | 0 | 0.13362734 |
| ORGANONITROGEN COMPOUND BIOSYNTHETIC PROCESS%GOBP%GO:1901566 | -1.44 | 0 | 0.13660896 |
| WNT%NETPATH%WNT | -1.44 | 0 | 0.13864778 |
| CHEMICAL HOMEOSTASIS%GOBP%GO:0048878 | -1.44 | 0 | 0.14104833 |
| ALCOHOL METABOLIC PROCESS%GOBP%GO:0006066 | -1.43 | 0 | 0.14110155 |
| REGULATION OF NUCLEOTIDE CATABOLIC PROCESS%GOBP%GO:0030811 | -1.44 | 0 | 0.14117569 |
| REGULATION OF PURINE NUCLEOTIDE CATABOLIC PROCESS%GOBP%GO:0033121 | -1.43 | 0 | 0.14216758 |
| POSITIVE REGULATION OF CELL PROLIFERATION%GOBP%GO:0008284 | -1.43 | 0 | 0.14552383 |
| REGULATION OF PROTEIN PROCESSING%GOBP%GO:0070613 | -1.42 | 0 | 0.14652123 |
| ACTIN FILAMENT-BASED PROCESS%GOBP%GO:0030029 | -1.42 | 0 | 0.14922687 |
| SENSORY ORGAN DEVELOPMENT%GOBP%GO:0007423 | -1.42 | 0 | 0.15083659 |
| PROTEIN PHOSPHORYLATION%GOBP%GO:0006468 | -1.42 | 0 | 0.15133615 |
| REGULATION OF NERVOUS SYSTEM DEVELOPMENT%GOBP%GO:0051960 | -1.41 | 0 | 0.1531512 |
| REGULATION OF RAS PROTEIN SIGNAL TRANSDUCTION%GOBP%GO:0046578 | -1.41 | 0 | 0.15380032 |
| SINGLE-ORGANISM MEMBRANE ORGANIZATION%GOBP%GO:0044802 | -1.41 | 0 | 0.15415931 |
| POSITIVE REGULATION OF MAPK CASCADE%GOBP%GO:0043410 | -1.40 | 0 | 0.1555987 |
| CARBOHYDRATE DERIVATIVE CATABOLIC PROCESS%GOBP%GO:1901136 | -1.40 | 0 | 0.15739515 |
| SMALL GTPASE MEDIATED SIGNAL TRANSDUCTION%GOBP%GO:0007264 | -1.40 | 0 | 0.15970244 |
| REGULATION OF MAPK CASCADE%GOBP%GO:0043408 | -1.39 | 0 | 0.1631537 |
| ORGANIC HYDROXY COMPOUND METABOLIC PROCESS%GOBP%GO:1901615 | -1.39 | 0 | 0.16876648 |
| PATHWAYS IN CANCER%KEGG%HSA05200 | -1.38 | 0 | 0.1731471 |
| CYTOSKELETON ORGANIZATION%GOBP%GO:0007010 | -1.37 | 0 | 0.17564659 |
| ANTIGEN PROCESSING AND PRESENTATION OF EXOGENOUS ANTIGEN%GOBP%GO:0019884 | -1.37 | 0 | 0.17803696 |
| NEGATIVE REGULATION OF PHOSPHATE METABOLIC PROCESS%GOBP%GO:0045936 | -1.37 | 0 | 0.17857732 |
| MONOCARBOXYLIC ACID METABOLIC PROCESS%GOBP%GO:0032787 | -1.36 | 0 | 0.18038121 |
| NEGATIVE REGULATION OF PHOSPHORYLATION%GOBP%GO:0042326 | -1.36 | 0 | 0.1818148 |

## Slide 9
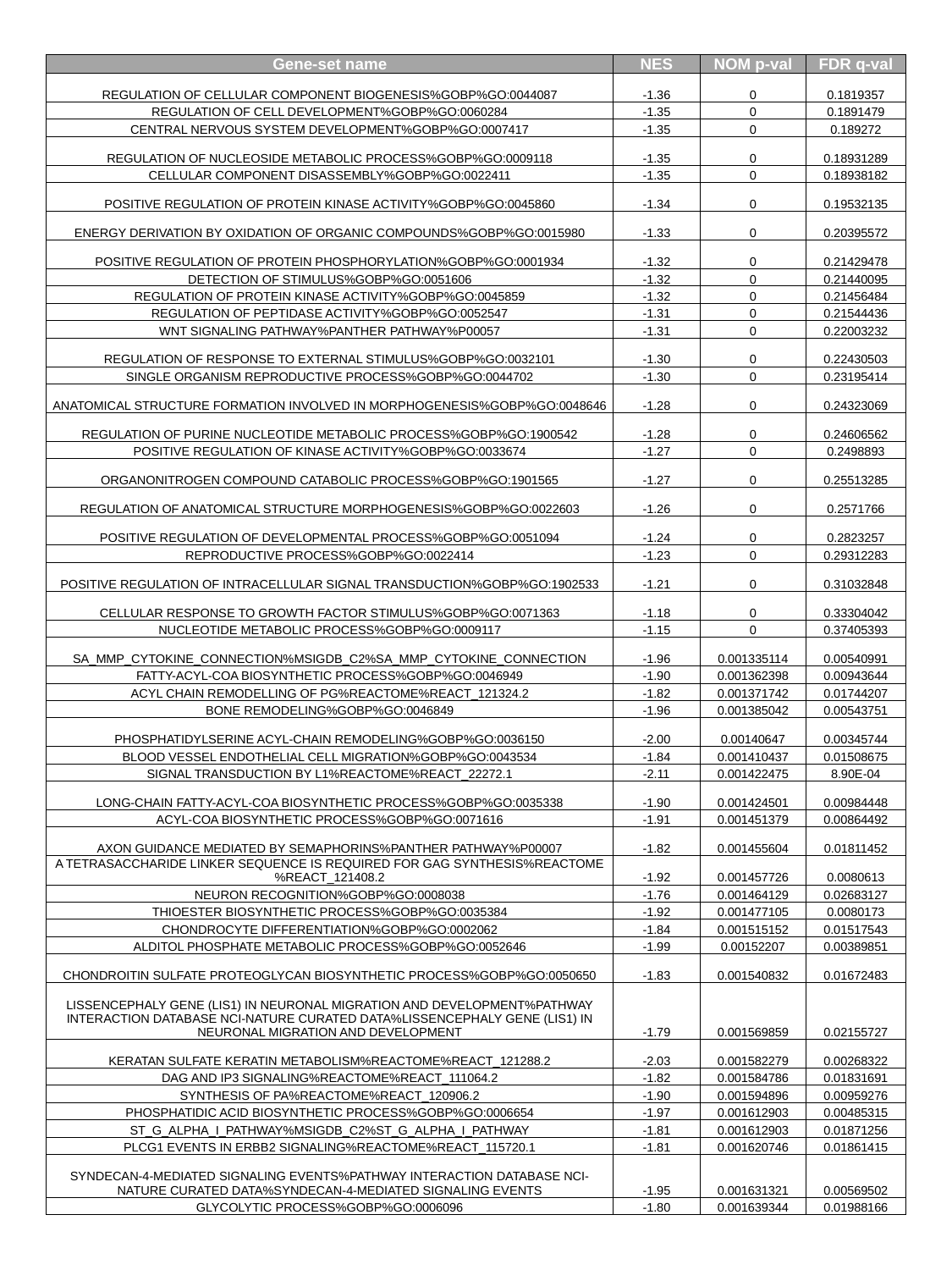

| Gene-set name | NES | NOM p-val | FDR q-val |
| --- | --- | --- | --- |
| REGULATION OF CELLULAR COMPONENT BIOGENESIS%GOBP%GO:0044087 | -1.36 | 0 | 0.1819357 |
| REGULATION OF CELL DEVELOPMENT%GOBP%GO:0060284 | -1.35 | 0 | 0.1891479 |
| CENTRAL NERVOUS SYSTEM DEVELOPMENT%GOBP%GO:0007417 | -1.35 | 0 | 0.189272 |
| REGULATION OF NUCLEOSIDE METABOLIC PROCESS%GOBP%GO:0009118 | -1.35 | 0 | 0.18931289 |
| CELLULAR COMPONENT DISASSEMBLY%GOBP%GO:0022411 | -1.35 | 0 | 0.18938182 |
| POSITIVE REGULATION OF PROTEIN KINASE ACTIVITY%GOBP%GO:0045860 | -1.34 | 0 | 0.19532135 |
| ENERGY DERIVATION BY OXIDATION OF ORGANIC COMPOUNDS%GOBP%GO:0015980 | -1.33 | 0 | 0.20395572 |
| POSITIVE REGULATION OF PROTEIN PHOSPHORYLATION%GOBP%GO:0001934 | -1.32 | 0 | 0.21429478 |
| DETECTION OF STIMULUS%GOBP%GO:0051606 | -1.32 | 0 | 0.21440095 |
| REGULATION OF PROTEIN KINASE ACTIVITY%GOBP%GO:0045859 | -1.32 | 0 | 0.21456484 |
| REGULATION OF PEPTIDASE ACTIVITY%GOBP%GO:0052547 | -1.31 | 0 | 0.21544436 |
| WNT SIGNALING PATHWAY%PANTHER PATHWAY%P00057 | -1.31 | 0 | 0.22003232 |
| REGULATION OF RESPONSE TO EXTERNAL STIMULUS%GOBP%GO:0032101 | -1.30 | 0 | 0.22430503 |
| SINGLE ORGANISM REPRODUCTIVE PROCESS%GOBP%GO:0044702 | -1.30 | 0 | 0.23195414 |
| ANATOMICAL STRUCTURE FORMATION INVOLVED IN MORPHOGENESIS%GOBP%GO:0048646 | -1.28 | 0 | 0.24323069 |
| REGULATION OF PURINE NUCLEOTIDE METABOLIC PROCESS%GOBP%GO:1900542 | -1.28 | 0 | 0.24606562 |
| POSITIVE REGULATION OF KINASE ACTIVITY%GOBP%GO:0033674 | -1.27 | 0 | 0.2498893 |
| ORGANONITROGEN COMPOUND CATABOLIC PROCESS%GOBP%GO:1901565 | -1.27 | 0 | 0.25513285 |
| REGULATION OF ANATOMICAL STRUCTURE MORPHOGENESIS%GOBP%GO:0022603 | -1.26 | 0 | 0.2571766 |
| POSITIVE REGULATION OF DEVELOPMENTAL PROCESS%GOBP%GO:0051094 | -1.24 | 0 | 0.2823257 |
| REPRODUCTIVE PROCESS%GOBP%GO:0022414 | -1.23 | 0 | 0.29312283 |
| POSITIVE REGULATION OF INTRACELLULAR SIGNAL TRANSDUCTION%GOBP%GO:1902533 | -1.21 | 0 | 0.31032848 |
| CELLULAR RESPONSE TO GROWTH FACTOR STIMULUS%GOBP%GO:0071363 | -1.18 | 0 | 0.33304042 |
| NUCLEOTIDE METABOLIC PROCESS%GOBP%GO:0009117 | -1.15 | 0 | 0.37405393 |
| SA\_MMP\_CYTOKINE\_CONNECTION%MSIGDB\_C2%SA\_MMP\_CYTOKINE\_CONNECTION | -1.96 | 0.001335114 | 0.00540991 |
| FATTY-ACYL-COA BIOSYNTHETIC PROCESS%GOBP%GO:0046949 | -1.90 | 0.001362398 | 0.00943644 |
| ACYL CHAIN REMODELLING OF PG%REACTOME%REACT\_121324.2 | -1.82 | 0.001371742 | 0.01744207 |
| BONE REMODELING%GOBP%GO:0046849 | -1.96 | 0.001385042 | 0.00543751 |
| PHOSPHATIDYLSERINE ACYL-CHAIN REMODELING%GOBP%GO:0036150 | -2.00 | 0.00140647 | 0.00345744 |
| BLOOD VESSEL ENDOTHELIAL CELL MIGRATION%GOBP%GO:0043534 | -1.84 | 0.001410437 | 0.01508675 |
| SIGNAL TRANSDUCTION BY L1%REACTOME%REACT\_22272.1 | -2.11 | 0.001422475 | 8.90E-04 |
| LONG-CHAIN FATTY-ACYL-COA BIOSYNTHETIC PROCESS%GOBP%GO:0035338 | -1.90 | 0.001424501 | 0.00984448 |
| ACYL-COA BIOSYNTHETIC PROCESS%GOBP%GO:0071616 | -1.91 | 0.001451379 | 0.00864492 |
| AXON GUIDANCE MEDIATED BY SEMAPHORINS%PANTHER PATHWAY%P00007 | -1.82 | 0.001455604 | 0.01811452 |
| A TETRASACCHARIDE LINKER SEQUENCE IS REQUIRED FOR GAG SYNTHESIS%REACTOME%REACT\_121408.2 | -1.92 | 0.001457726 | 0.0080613 |
| NEURON RECOGNITION%GOBP%GO:0008038 | -1.76 | 0.001464129 | 0.02683127 |
| THIOESTER BIOSYNTHETIC PROCESS%GOBP%GO:0035384 | -1.92 | 0.001477105 | 0.0080173 |
| CHONDROCYTE DIFFERENTIATION%GOBP%GO:0002062 | -1.84 | 0.001515152 | 0.01517543 |
| ALDITOL PHOSPHATE METABOLIC PROCESS%GOBP%GO:0052646 | -1.99 | 0.00152207 | 0.00389851 |
| CHONDROITIN SULFATE PROTEOGLYCAN BIOSYNTHETIC PROCESS%GOBP%GO:0050650 | -1.83 | 0.001540832 | 0.01672483 |
| LISSENCEPHALY GENE (LIS1) IN NEURONAL MIGRATION AND DEVELOPMENT%PATHWAY INTERACTION DATABASE NCI-NATURE CURATED DATA%LISSENCEPHALY GENE (LIS1) IN NEURONAL MIGRATION AND DEVELOPMENT | -1.79 | 0.001569859 | 0.02155727 |
| KERATAN SULFATE KERATIN METABOLISM%REACTOME%REACT\_121288.2 | -2.03 | 0.001582279 | 0.00268322 |
| DAG AND IP3 SIGNALING%REACTOME%REACT\_111064.2 | -1.82 | 0.001584786 | 0.01831691 |
| SYNTHESIS OF PA%REACTOME%REACT\_120906.2 | -1.90 | 0.001594896 | 0.00959276 |
| PHOSPHATIDIC ACID BIOSYNTHETIC PROCESS%GOBP%GO:0006654 | -1.97 | 0.001612903 | 0.00485315 |
| ST\_G\_ALPHA\_I\_PATHWAY%MSIGDB\_C2%ST\_G\_ALPHA\_I\_PATHWAY | -1.81 | 0.001612903 | 0.01871256 |
| PLCG1 EVENTS IN ERBB2 SIGNALING%REACTOME%REACT\_115720.1 | -1.81 | 0.001620746 | 0.01861415 |
| SYNDECAN-4-MEDIATED SIGNALING EVENTS%PATHWAY INTERACTION DATABASE NCI-NATURE CURATED DATA%SYNDECAN-4-MEDIATED SIGNALING EVENTS | -1.95 | 0.001631321 | 0.00569502 |
| GLYCOLYTIC PROCESS%GOBP%GO:0006096 | -1.80 | 0.001639344 | 0.01988166 |

## Slide 10
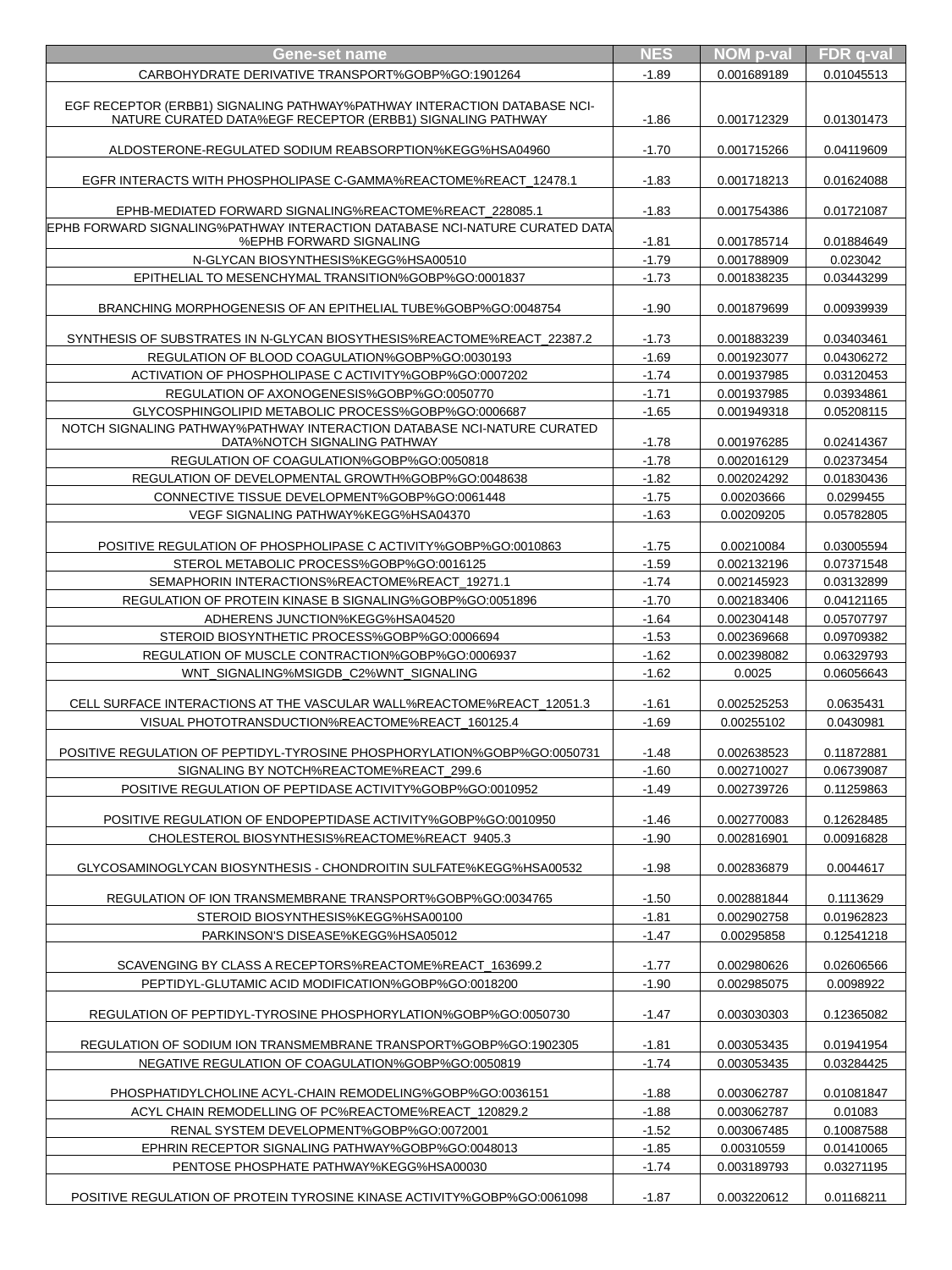

| Gene-set name | NES | NOM p-val | FDR q-val |
| --- | --- | --- | --- |
| CARBOHYDRATE DERIVATIVE TRANSPORT%GOBP%GO:1901264 | -1.89 | 0.001689189 | 0.01045513 |
| EGF RECEPTOR (ERBB1) SIGNALING PATHWAY%PATHWAY INTERACTION DATABASE NCI-NATURE CURATED DATA%EGF RECEPTOR (ERBB1) SIGNALING PATHWAY | -1.86 | 0.001712329 | 0.01301473 |
| ALDOSTERONE-REGULATED SODIUM REABSORPTION%KEGG%HSA04960 | -1.70 | 0.001715266 | 0.04119609 |
| EGFR INTERACTS WITH PHOSPHOLIPASE C-GAMMA%REACTOME%REACT\_12478.1 | -1.83 | 0.001718213 | 0.01624088 |
| EPHB-MEDIATED FORWARD SIGNALING%REACTOME%REACT\_228085.1 | -1.83 | 0.001754386 | 0.01721087 |
| EPHB FORWARD SIGNALING%PATHWAY INTERACTION DATABASE NCI-NATURE CURATED DATA%EPHB FORWARD SIGNALING | -1.81 | 0.001785714 | 0.01884649 |
| N-GLYCAN BIOSYNTHESIS%KEGG%HSA00510 | -1.79 | 0.001788909 | 0.023042 |
| EPITHELIAL TO MESENCHYMAL TRANSITION%GOBP%GO:0001837 | -1.73 | 0.001838235 | 0.03443299 |
| BRANCHING MORPHOGENESIS OF AN EPITHELIAL TUBE%GOBP%GO:0048754 | -1.90 | 0.001879699 | 0.00939939 |
| SYNTHESIS OF SUBSTRATES IN N-GLYCAN BIOSYTHESIS%REACTOME%REACT\_22387.2 | -1.73 | 0.001883239 | 0.03403461 |
| REGULATION OF BLOOD COAGULATION%GOBP%GO:0030193 | -1.69 | 0.001923077 | 0.04306272 |
| ACTIVATION OF PHOSPHOLIPASE C ACTIVITY%GOBP%GO:0007202 | -1.74 | 0.001937985 | 0.03120453 |
| REGULATION OF AXONOGENESIS%GOBP%GO:0050770 | -1.71 | 0.001937985 | 0.03934861 |
| GLYCOSPHINGOLIPID METABOLIC PROCESS%GOBP%GO:0006687 | -1.65 | 0.001949318 | 0.05208115 |
| NOTCH SIGNALING PATHWAY%PATHWAY INTERACTION DATABASE NCI-NATURE CURATED DATA%NOTCH SIGNALING PATHWAY | -1.78 | 0.001976285 | 0.02414367 |
| REGULATION OF COAGULATION%GOBP%GO:0050818 | -1.78 | 0.002016129 | 0.02373454 |
| REGULATION OF DEVELOPMENTAL GROWTH%GOBP%GO:0048638 | -1.82 | 0.002024292 | 0.01830436 |
| CONNECTIVE TISSUE DEVELOPMENT%GOBP%GO:0061448 | -1.75 | 0.00203666 | 0.0299455 |
| VEGF SIGNALING PATHWAY%KEGG%HSA04370 | -1.63 | 0.00209205 | 0.05782805 |
| POSITIVE REGULATION OF PHOSPHOLIPASE C ACTIVITY%GOBP%GO:0010863 | -1.75 | 0.00210084 | 0.03005594 |
| STEROL METABOLIC PROCESS%GOBP%GO:0016125 | -1.59 | 0.002132196 | 0.07371548 |
| SEMAPHORIN INTERACTIONS%REACTOME%REACT\_19271.1 | -1.74 | 0.002145923 | 0.03132899 |
| REGULATION OF PROTEIN KINASE B SIGNALING%GOBP%GO:0051896 | -1.70 | 0.002183406 | 0.04121165 |
| ADHERENS JUNCTION%KEGG%HSA04520 | -1.64 | 0.002304148 | 0.05707797 |
| STEROID BIOSYNTHETIC PROCESS%GOBP%GO:0006694 | -1.53 | 0.002369668 | 0.09709382 |
| REGULATION OF MUSCLE CONTRACTION%GOBP%GO:0006937 | -1.62 | 0.002398082 | 0.06329793 |
| WNT\_SIGNALING%MSIGDB\_C2%WNT\_SIGNALING | -1.62 | 0.0025 | 0.06056643 |
| CELL SURFACE INTERACTIONS AT THE VASCULAR WALL%REACTOME%REACT\_12051.3 | -1.61 | 0.002525253 | 0.0635431 |
| VISUAL PHOTOTRANSDUCTION%REACTOME%REACT\_160125.4 | -1.69 | 0.00255102 | 0.0430981 |
| POSITIVE REGULATION OF PEPTIDYL-TYROSINE PHOSPHORYLATION%GOBP%GO:0050731 | -1.48 | 0.002638523 | 0.11872881 |
| SIGNALING BY NOTCH%REACTOME%REACT\_299.6 | -1.60 | 0.002710027 | 0.06739087 |
| POSITIVE REGULATION OF PEPTIDASE ACTIVITY%GOBP%GO:0010952 | -1.49 | 0.002739726 | 0.11259863 |
| POSITIVE REGULATION OF ENDOPEPTIDASE ACTIVITY%GOBP%GO:0010950 | -1.46 | 0.002770083 | 0.12628485 |
| CHOLESTEROL BIOSYNTHESIS%REACTOME%REACT\_9405.3 | -1.90 | 0.002816901 | 0.00916828 |
| GLYCOSAMINOGLYCAN BIOSYNTHESIS - CHONDROITIN SULFATE%KEGG%HSA00532 | -1.98 | 0.002836879 | 0.0044617 |
| REGULATION OF ION TRANSMEMBRANE TRANSPORT%GOBP%GO:0034765 | -1.50 | 0.002881844 | 0.1113629 |
| STEROID BIOSYNTHESIS%KEGG%HSA00100 | -1.81 | 0.002902758 | 0.01962823 |
| PARKINSON'S DISEASE%KEGG%HSA05012 | -1.47 | 0.00295858 | 0.12541218 |
| SCAVENGING BY CLASS A RECEPTORS%REACTOME%REACT\_163699.2 | -1.77 | 0.002980626 | 0.02606566 |
| PEPTIDYL-GLUTAMIC ACID MODIFICATION%GOBP%GO:0018200 | -1.90 | 0.002985075 | 0.0098922 |
| REGULATION OF PEPTIDYL-TYROSINE PHOSPHORYLATION%GOBP%GO:0050730 | -1.47 | 0.003030303 | 0.12365082 |
| REGULATION OF SODIUM ION TRANSMEMBRANE TRANSPORT%GOBP%GO:1902305 | -1.81 | 0.003053435 | 0.01941954 |
| NEGATIVE REGULATION OF COAGULATION%GOBP%GO:0050819 | -1.74 | 0.003053435 | 0.03284425 |
| PHOSPHATIDYLCHOLINE ACYL-CHAIN REMODELING%GOBP%GO:0036151 | -1.88 | 0.003062787 | 0.01081847 |
| ACYL CHAIN REMODELLING OF PC%REACTOME%REACT\_120829.2 | -1.88 | 0.003062787 | 0.01083 |
| RENAL SYSTEM DEVELOPMENT%GOBP%GO:0072001 | -1.52 | 0.003067485 | 0.10087588 |
| EPHRIN RECEPTOR SIGNALING PATHWAY%GOBP%GO:0048013 | -1.85 | 0.00310559 | 0.01410065 |
| PENTOSE PHOSPHATE PATHWAY%KEGG%HSA00030 | -1.74 | 0.003189793 | 0.03271195 |
| POSITIVE REGULATION OF PROTEIN TYROSINE KINASE ACTIVITY%GOBP%GO:0061098 | -1.87 | 0.003220612 | 0.01168211 |

## Slide 11
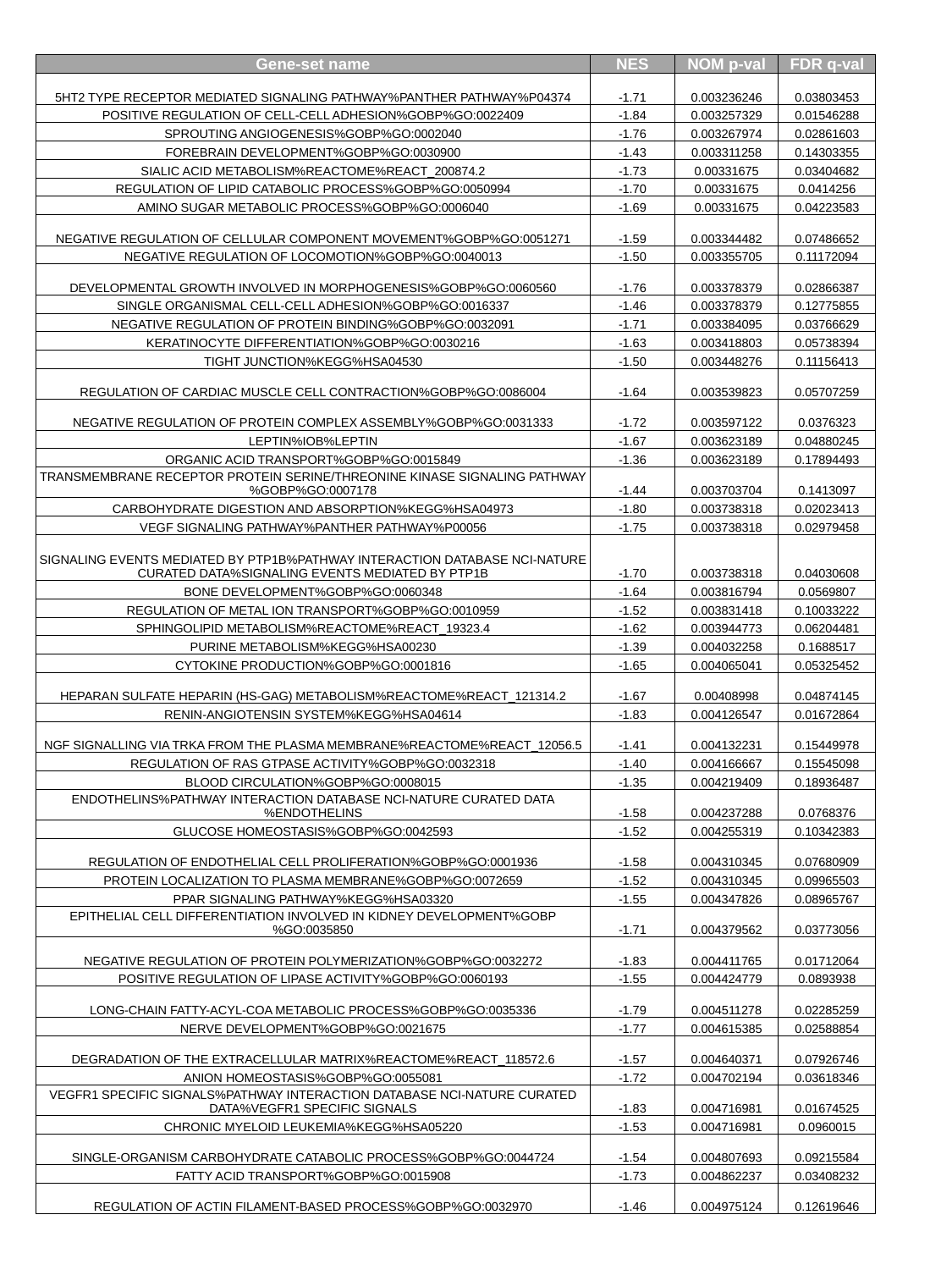

| Gene-set name | NES | NOM p-val | FDR q-val |
| --- | --- | --- | --- |
| 5HT2 TYPE RECEPTOR MEDIATED SIGNALING PATHWAY%PANTHER PATHWAY%P04374 | -1.71 | 0.003236246 | 0.03803453 |
| POSITIVE REGULATION OF CELL-CELL ADHESION%GOBP%GO:0022409 | -1.84 | 0.003257329 | 0.01546288 |
| SPROUTING ANGIOGENESIS%GOBP%GO:0002040 | -1.76 | 0.003267974 | 0.02861603 |
| FOREBRAIN DEVELOPMENT%GOBP%GO:0030900 | -1.43 | 0.003311258 | 0.14303355 |
| SIALIC ACID METABOLISM%REACTOME%REACT\_200874.2 | -1.73 | 0.00331675 | 0.03404682 |
| REGULATION OF LIPID CATABOLIC PROCESS%GOBP%GO:0050994 | -1.70 | 0.00331675 | 0.0414256 |
| AMINO SUGAR METABOLIC PROCESS%GOBP%GO:0006040 | -1.69 | 0.00331675 | 0.04223583 |
| NEGATIVE REGULATION OF CELLULAR COMPONENT MOVEMENT%GOBP%GO:0051271 | -1.59 | 0.003344482 | 0.07486652 |
| NEGATIVE REGULATION OF LOCOMOTION%GOBP%GO:0040013 | -1.50 | 0.003355705 | 0.11172094 |
| DEVELOPMENTAL GROWTH INVOLVED IN MORPHOGENESIS%GOBP%GO:0060560 | -1.76 | 0.003378379 | 0.02866387 |
| SINGLE ORGANISMAL CELL-CELL ADHESION%GOBP%GO:0016337 | -1.46 | 0.003378379 | 0.12775855 |
| NEGATIVE REGULATION OF PROTEIN BINDING%GOBP%GO:0032091 | -1.71 | 0.003384095 | 0.03766629 |
| KERATINOCYTE DIFFERENTIATION%GOBP%GO:0030216 | -1.63 | 0.003418803 | 0.05738394 |
| TIGHT JUNCTION%KEGG%HSA04530 | -1.50 | 0.003448276 | 0.11156413 |
| REGULATION OF CARDIAC MUSCLE CELL CONTRACTION%GOBP%GO:0086004 | -1.64 | 0.003539823 | 0.05707259 |
| NEGATIVE REGULATION OF PROTEIN COMPLEX ASSEMBLY%GOBP%GO:0031333 | -1.72 | 0.003597122 | 0.0376323 |
| LEPTIN%IOB%LEPTIN | -1.67 | 0.003623189 | 0.04880245 |
| ORGANIC ACID TRANSPORT%GOBP%GO:0015849 | -1.36 | 0.003623189 | 0.17894493 |
| TRANSMEMBRANE RECEPTOR PROTEIN SERINE/THREONINE KINASE SIGNALING PATHWAY%GOBP%GO:0007178 | -1.44 | 0.003703704 | 0.1413097 |
| CARBOHYDRATE DIGESTION AND ABSORPTION%KEGG%HSA04973 | -1.80 | 0.003738318 | 0.02023413 |
| VEGF SIGNALING PATHWAY%PANTHER PATHWAY%P00056 | -1.75 | 0.003738318 | 0.02979458 |
| SIGNALING EVENTS MEDIATED BY PTP1B%PATHWAY INTERACTION DATABASE NCI-NATURE CURATED DATA%SIGNALING EVENTS MEDIATED BY PTP1B | -1.70 | 0.003738318 | 0.04030608 |
| BONE DEVELOPMENT%GOBP%GO:0060348 | -1.64 | 0.003816794 | 0.0569807 |
| REGULATION OF METAL ION TRANSPORT%GOBP%GO:0010959 | -1.52 | 0.003831418 | 0.10033222 |
| SPHINGOLIPID METABOLISM%REACTOME%REACT\_19323.4 | -1.62 | 0.003944773 | 0.06204481 |
| PURINE METABOLISM%KEGG%HSA00230 | -1.39 | 0.004032258 | 0.1688517 |
| CYTOKINE PRODUCTION%GOBP%GO:0001816 | -1.65 | 0.004065041 | 0.05325452 |
| HEPARAN SULFATE HEPARIN (HS-GAG) METABOLISM%REACTOME%REACT\_121314.2 | -1.67 | 0.00408998 | 0.04874145 |
| RENIN-ANGIOTENSIN SYSTEM%KEGG%HSA04614 | -1.83 | 0.004126547 | 0.01672864 |
| NGF SIGNALLING VIA TRKA FROM THE PLASMA MEMBRANE%REACTOME%REACT\_12056.5 | -1.41 | 0.004132231 | 0.15449978 |
| REGULATION OF RAS GTPASE ACTIVITY%GOBP%GO:0032318 | -1.40 | 0.004166667 | 0.15545098 |
| BLOOD CIRCULATION%GOBP%GO:0008015 | -1.35 | 0.004219409 | 0.18936487 |
| ENDOTHELINS%PATHWAY INTERACTION DATABASE NCI-NATURE CURATED DATA%ENDOTHELINS | -1.58 | 0.004237288 | 0.0768376 |
| GLUCOSE HOMEOSTASIS%GOBP%GO:0042593 | -1.52 | 0.004255319 | 0.10342383 |
| REGULATION OF ENDOTHELIAL CELL PROLIFERATION%GOBP%GO:0001936 | -1.58 | 0.004310345 | 0.07680909 |
| PROTEIN LOCALIZATION TO PLASMA MEMBRANE%GOBP%GO:0072659 | -1.52 | 0.004310345 | 0.09965503 |
| PPAR SIGNALING PATHWAY%KEGG%HSA03320 | -1.55 | 0.004347826 | 0.08965767 |
| EPITHELIAL CELL DIFFERENTIATION INVOLVED IN KIDNEY DEVELOPMENT%GOBP%GO:0035850 | -1.71 | 0.004379562 | 0.03773056 |
| NEGATIVE REGULATION OF PROTEIN POLYMERIZATION%GOBP%GO:0032272 | -1.83 | 0.004411765 | 0.01712064 |
| POSITIVE REGULATION OF LIPASE ACTIVITY%GOBP%GO:0060193 | -1.55 | 0.004424779 | 0.0893938 |
| LONG-CHAIN FATTY-ACYL-COA METABOLIC PROCESS%GOBP%GO:0035336 | -1.79 | 0.004511278 | 0.02285259 |
| NERVE DEVELOPMENT%GOBP%GO:0021675 | -1.77 | 0.004615385 | 0.02588854 |
| DEGRADATION OF THE EXTRACELLULAR MATRIX%REACTOME%REACT\_118572.6 | -1.57 | 0.004640371 | 0.07926746 |
| ANION HOMEOSTASIS%GOBP%GO:0055081 | -1.72 | 0.004702194 | 0.03618346 |
| VEGFR1 SPECIFIC SIGNALS%PATHWAY INTERACTION DATABASE NCI-NATURE CURATED DATA%VEGFR1 SPECIFIC SIGNALS | -1.83 | 0.004716981 | 0.01674525 |
| CHRONIC MYELOID LEUKEMIA%KEGG%HSA05220 | -1.53 | 0.004716981 | 0.0960015 |
| SINGLE-ORGANISM CARBOHYDRATE CATABOLIC PROCESS%GOBP%GO:0044724 | -1.54 | 0.004807693 | 0.09215584 |
| FATTY ACID TRANSPORT%GOBP%GO:0015908 | -1.73 | 0.004862237 | 0.03408232 |
| REGULATION OF ACTIN FILAMENT-BASED PROCESS%GOBP%GO:0032970 | -1.46 | 0.004975124 | 0.12619646 |

## Slide 12
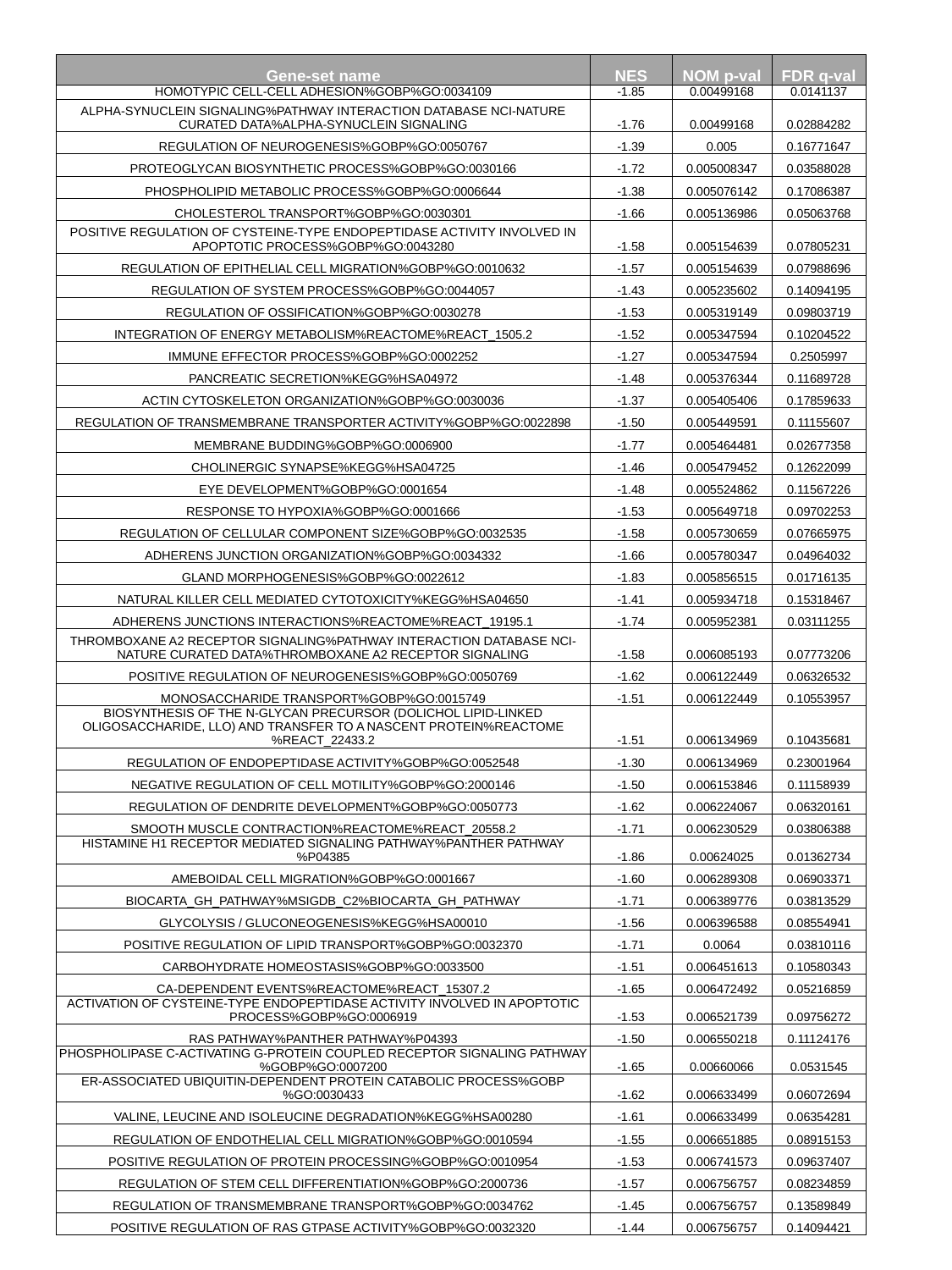

| Gene-set name | NES | NOM p-val | FDR q-val |
| --- | --- | --- | --- |
| HOMOTYPIC CELL-CELL ADHESION%GOBP%GO:0034109 | -1.85 | 0.00499168 | 0.0141137 |
| ALPHA-SYNUCLEIN SIGNALING%PATHWAY INTERACTION DATABASE NCI-NATURE CURATED DATA%ALPHA-SYNUCLEIN SIGNALING | -1.76 | 0.00499168 | 0.02884282 |
| REGULATION OF NEUROGENESIS%GOBP%GO:0050767 | -1.39 | 0.005 | 0.16771647 |
| PROTEOGLYCAN BIOSYNTHETIC PROCESS%GOBP%GO:0030166 | -1.72 | 0.005008347 | 0.03588028 |
| PHOSPHOLIPID METABOLIC PROCESS%GOBP%GO:0006644 | -1.38 | 0.005076142 | 0.17086387 |
| CHOLESTEROL TRANSPORT%GOBP%GO:0030301 | -1.66 | 0.005136986 | 0.05063768 |
| POSITIVE REGULATION OF CYSTEINE-TYPE ENDOPEPTIDASE ACTIVITY INVOLVED IN APOPTOTIC PROCESS%GOBP%GO:0043280 | -1.58 | 0.005154639 | 0.07805231 |
| REGULATION OF EPITHELIAL CELL MIGRATION%GOBP%GO:0010632 | -1.57 | 0.005154639 | 0.07988696 |
| REGULATION OF SYSTEM PROCESS%GOBP%GO:0044057 | -1.43 | 0.005235602 | 0.14094195 |
| REGULATION OF OSSIFICATION%GOBP%GO:0030278 | -1.53 | 0.005319149 | 0.09803719 |
| INTEGRATION OF ENERGY METABOLISM%REACTOME%REACT\_1505.2 | -1.52 | 0.005347594 | 0.10204522 |
| IMMUNE EFFECTOR PROCESS%GOBP%GO:0002252 | -1.27 | 0.005347594 | 0.2505997 |
| PANCREATIC SECRETION%KEGG%HSA04972 | -1.48 | 0.005376344 | 0.11689728 |
| ACTIN CYTOSKELETON ORGANIZATION%GOBP%GO:0030036 | -1.37 | 0.005405406 | 0.17859633 |
| REGULATION OF TRANSMEMBRANE TRANSPORTER ACTIVITY%GOBP%GO:0022898 | -1.50 | 0.005449591 | 0.11155607 |
| MEMBRANE BUDDING%GOBP%GO:0006900 | -1.77 | 0.005464481 | 0.02677358 |
| CHOLINERGIC SYNAPSE%KEGG%HSA04725 | -1.46 | 0.005479452 | 0.12622099 |
| EYE DEVELOPMENT%GOBP%GO:0001654 | -1.48 | 0.005524862 | 0.11567226 |
| RESPONSE TO HYPOXIA%GOBP%GO:0001666 | -1.53 | 0.005649718 | 0.09702253 |
| REGULATION OF CELLULAR COMPONENT SIZE%GOBP%GO:0032535 | -1.58 | 0.005730659 | 0.07665975 |
| ADHERENS JUNCTION ORGANIZATION%GOBP%GO:0034332 | -1.66 | 0.005780347 | 0.04964032 |
| GLAND MORPHOGENESIS%GOBP%GO:0022612 | -1.83 | 0.005856515 | 0.01716135 |
| NATURAL KILLER CELL MEDIATED CYTOTOXICITY%KEGG%HSA04650 | -1.41 | 0.005934718 | 0.15318467 |
| ADHERENS JUNCTIONS INTERACTIONS%REACTOME%REACT\_19195.1 | -1.74 | 0.005952381 | 0.03111255 |
| THROMBOXANE A2 RECEPTOR SIGNALING%PATHWAY INTERACTION DATABASE NCI-NATURE CURATED DATA%THROMBOXANE A2 RECEPTOR SIGNALING | -1.58 | 0.006085193 | 0.07773206 |
| POSITIVE REGULATION OF NEUROGENESIS%GOBP%GO:0050769 | -1.62 | 0.006122449 | 0.06326532 |
| MONOSACCHARIDE TRANSPORT%GOBP%GO:0015749 | -1.51 | 0.006122449 | 0.10553957 |
| BIOSYNTHESIS OF THE N-GLYCAN PRECURSOR (DOLICHOL LIPID-LINKED OLIGOSACCHARIDE, LLO) AND TRANSFER TO A NASCENT PROTEIN%REACTOME%REACT\_22433.2 | -1.51 | 0.006134969 | 0.10435681 |
| REGULATION OF ENDOPEPTIDASE ACTIVITY%GOBP%GO:0052548 | -1.30 | 0.006134969 | 0.23001964 |
| NEGATIVE REGULATION OF CELL MOTILITY%GOBP%GO:2000146 | -1.50 | 0.006153846 | 0.11158939 |
| REGULATION OF DENDRITE DEVELOPMENT%GOBP%GO:0050773 | -1.62 | 0.006224067 | 0.06320161 |
| SMOOTH MUSCLE CONTRACTION%REACTOME%REACT\_20558.2 | -1.71 | 0.006230529 | 0.03806388 |
| HISTAMINE H1 RECEPTOR MEDIATED SIGNALING PATHWAY%PANTHER PATHWAY%P04385 | -1.86 | 0.00624025 | 0.01362734 |
| AMEBOIDAL CELL MIGRATION%GOBP%GO:0001667 | -1.60 | 0.006289308 | 0.06903371 |
| BIOCARTA\_GH\_PATHWAY%MSIGDB\_C2%BIOCARTA\_GH\_PATHWAY | -1.71 | 0.006389776 | 0.03813529 |
| GLYCOLYSIS / GLUCONEOGENESIS%KEGG%HSA00010 | -1.56 | 0.006396588 | 0.08554941 |
| POSITIVE REGULATION OF LIPID TRANSPORT%GOBP%GO:0032370 | -1.71 | 0.0064 | 0.03810116 |
| CARBOHYDRATE HOMEOSTASIS%GOBP%GO:0033500 | -1.51 | 0.006451613 | 0.10580343 |
| CA-DEPENDENT EVENTS%REACTOME%REACT\_15307.2 | -1.65 | 0.006472492 | 0.05216859 |
| ACTIVATION OF CYSTEINE-TYPE ENDOPEPTIDASE ACTIVITY INVOLVED IN APOPTOTIC PROCESS%GOBP%GO:0006919 | -1.53 | 0.006521739 | 0.09756272 |
| RAS PATHWAY%PANTHER PATHWAY%P04393 | -1.50 | 0.006550218 | 0.11124176 |
| PHOSPHOLIPASE C-ACTIVATING G-PROTEIN COUPLED RECEPTOR SIGNALING PATHWAY%GOBP%GO:0007200 | -1.65 | 0.00660066 | 0.0531545 |
| ER-ASSOCIATED UBIQUITIN-DEPENDENT PROTEIN CATABOLIC PROCESS%GOBP%GO:0030433 | -1.62 | 0.006633499 | 0.06072694 |
| VALINE, LEUCINE AND ISOLEUCINE DEGRADATION%KEGG%HSA00280 | -1.61 | 0.006633499 | 0.06354281 |
| REGULATION OF ENDOTHELIAL CELL MIGRATION%GOBP%GO:0010594 | -1.55 | 0.006651885 | 0.08915153 |
| POSITIVE REGULATION OF PROTEIN PROCESSING%GOBP%GO:0010954 | -1.53 | 0.006741573 | 0.09637407 |
| REGULATION OF STEM CELL DIFFERENTIATION%GOBP%GO:2000736 | -1.57 | 0.006756757 | 0.08234859 |
| REGULATION OF TRANSMEMBRANE TRANSPORT%GOBP%GO:0034762 | -1.45 | 0.006756757 | 0.13589849 |
| POSITIVE REGULATION OF RAS GTPASE ACTIVITY%GOBP%GO:0032320 | -1.44 | 0.006756757 | 0.14094421 |

## Slide 13
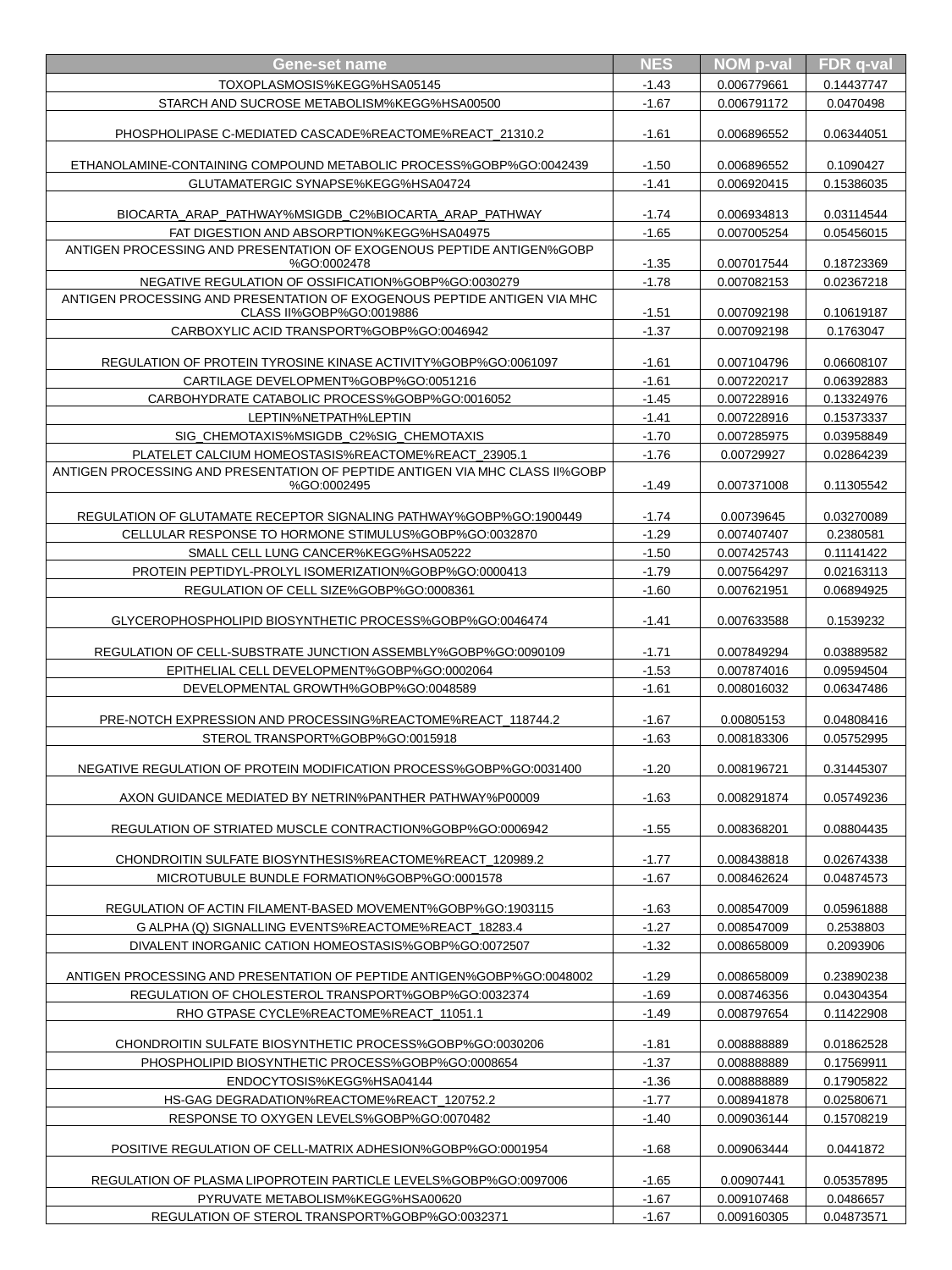

| Gene-set name | NES | NOM p-val | FDR q-val |
| --- | --- | --- | --- |
| TOXOPLASMOSIS%KEGG%HSA05145 | -1.43 | 0.006779661 | 0.14437747 |
| STARCH AND SUCROSE METABOLISM%KEGG%HSA00500 | -1.67 | 0.006791172 | 0.0470498 |
| PHOSPHOLIPASE C-MEDIATED CASCADE%REACTOME%REACT\_21310.2 | -1.61 | 0.006896552 | 0.06344051 |
| ETHANOLAMINE-CONTAINING COMPOUND METABOLIC PROCESS%GOBP%GO:0042439 | -1.50 | 0.006896552 | 0.1090427 |
| GLUTAMATERGIC SYNAPSE%KEGG%HSA04724 | -1.41 | 0.006920415 | 0.15386035 |
| BIOCARTA\_ARAP\_PATHWAY%MSIGDB\_C2%BIOCARTA\_ARAP\_PATHWAY | -1.74 | 0.006934813 | 0.03114544 |
| FAT DIGESTION AND ABSORPTION%KEGG%HSA04975 | -1.65 | 0.007005254 | 0.05456015 |
| ANTIGEN PROCESSING AND PRESENTATION OF EXOGENOUS PEPTIDE ANTIGEN%GOBP%GO:0002478 | -1.35 | 0.007017544 | 0.18723369 |
| NEGATIVE REGULATION OF OSSIFICATION%GOBP%GO:0030279 | -1.78 | 0.007082153 | 0.02367218 |
| ANTIGEN PROCESSING AND PRESENTATION OF EXOGENOUS PEPTIDE ANTIGEN VIA MHC CLASS II%GOBP%GO:0019886 | -1.51 | 0.007092198 | 0.10619187 |
| CARBOXYLIC ACID TRANSPORT%GOBP%GO:0046942 | -1.37 | 0.007092198 | 0.1763047 |
| REGULATION OF PROTEIN TYROSINE KINASE ACTIVITY%GOBP%GO:0061097 | -1.61 | 0.007104796 | 0.06608107 |
| CARTILAGE DEVELOPMENT%GOBP%GO:0051216 | -1.61 | 0.007220217 | 0.06392883 |
| CARBOHYDRATE CATABOLIC PROCESS%GOBP%GO:0016052 | -1.45 | 0.007228916 | 0.13324976 |
| LEPTIN%NETPATH%LEPTIN | -1.41 | 0.007228916 | 0.15373337 |
| SIG\_CHEMOTAXIS%MSIGDB\_C2%SIG\_CHEMOTAXIS | -1.70 | 0.007285975 | 0.03958849 |
| PLATELET CALCIUM HOMEOSTASIS%REACTOME%REACT\_23905.1 | -1.76 | 0.00729927 | 0.02864239 |
| ANTIGEN PROCESSING AND PRESENTATION OF PEPTIDE ANTIGEN VIA MHC CLASS II%GOBP%GO:0002495 | -1.49 | 0.007371008 | 0.11305542 |
| REGULATION OF GLUTAMATE RECEPTOR SIGNALING PATHWAY%GOBP%GO:1900449 | -1.74 | 0.00739645 | 0.03270089 |
| CELLULAR RESPONSE TO HORMONE STIMULUS%GOBP%GO:0032870 | -1.29 | 0.007407407 | 0.2380581 |
| SMALL CELL LUNG CANCER%KEGG%HSA05222 | -1.50 | 0.007425743 | 0.11141422 |
| PROTEIN PEPTIDYL-PROLYL ISOMERIZATION%GOBP%GO:0000413 | -1.79 | 0.007564297 | 0.02163113 |
| REGULATION OF CELL SIZE%GOBP%GO:0008361 | -1.60 | 0.007621951 | 0.06894925 |
| GLYCEROPHOSPHOLIPID BIOSYNTHETIC PROCESS%GOBP%GO:0046474 | -1.41 | 0.007633588 | 0.1539232 |
| REGULATION OF CELL-SUBSTRATE JUNCTION ASSEMBLY%GOBP%GO:0090109 | -1.71 | 0.007849294 | 0.03889582 |
| EPITHELIAL CELL DEVELOPMENT%GOBP%GO:0002064 | -1.53 | 0.007874016 | 0.09594504 |
| DEVELOPMENTAL GROWTH%GOBP%GO:0048589 | -1.61 | 0.008016032 | 0.06347486 |
| PRE-NOTCH EXPRESSION AND PROCESSING%REACTOME%REACT\_118744.2 | -1.67 | 0.00805153 | 0.04808416 |
| STEROL TRANSPORT%GOBP%GO:0015918 | -1.63 | 0.008183306 | 0.05752995 |
| NEGATIVE REGULATION OF PROTEIN MODIFICATION PROCESS%GOBP%GO:0031400 | -1.20 | 0.008196721 | 0.31445307 |
| AXON GUIDANCE MEDIATED BY NETRIN%PANTHER PATHWAY%P00009 | -1.63 | 0.008291874 | 0.05749236 |
| REGULATION OF STRIATED MUSCLE CONTRACTION%GOBP%GO:0006942 | -1.55 | 0.008368201 | 0.08804435 |
| CHONDROITIN SULFATE BIOSYNTHESIS%REACTOME%REACT\_120989.2 | -1.77 | 0.008438818 | 0.02674338 |
| MICROTUBULE BUNDLE FORMATION%GOBP%GO:0001578 | -1.67 | 0.008462624 | 0.04874573 |
| REGULATION OF ACTIN FILAMENT-BASED MOVEMENT%GOBP%GO:1903115 | -1.63 | 0.008547009 | 0.05961888 |
| G ALPHA (Q) SIGNALLING EVENTS%REACTOME%REACT\_18283.4 | -1.27 | 0.008547009 | 0.2538803 |
| DIVALENT INORGANIC CATION HOMEOSTASIS%GOBP%GO:0072507 | -1.32 | 0.008658009 | 0.2093906 |
| ANTIGEN PROCESSING AND PRESENTATION OF PEPTIDE ANTIGEN%GOBP%GO:0048002 | -1.29 | 0.008658009 | 0.23890238 |
| REGULATION OF CHOLESTEROL TRANSPORT%GOBP%GO:0032374 | -1.69 | 0.008746356 | 0.04304354 |
| RHO GTPASE CYCLE%REACTOME%REACT\_11051.1 | -1.49 | 0.008797654 | 0.11422908 |
| CHONDROITIN SULFATE BIOSYNTHETIC PROCESS%GOBP%GO:0030206 | -1.81 | 0.008888889 | 0.01862528 |
| PHOSPHOLIPID BIOSYNTHETIC PROCESS%GOBP%GO:0008654 | -1.37 | 0.008888889 | 0.17569911 |
| ENDOCYTOSIS%KEGG%HSA04144 | -1.36 | 0.008888889 | 0.17905822 |
| HS-GAG DEGRADATION%REACTOME%REACT\_120752.2 | -1.77 | 0.008941878 | 0.02580671 |
| RESPONSE TO OXYGEN LEVELS%GOBP%GO:0070482 | -1.40 | 0.009036144 | 0.15708219 |
| POSITIVE REGULATION OF CELL-MATRIX ADHESION%GOBP%GO:0001954 | -1.68 | 0.009063444 | 0.0441872 |
| REGULATION OF PLASMA LIPOPROTEIN PARTICLE LEVELS%GOBP%GO:0097006 | -1.65 | 0.00907441 | 0.05357895 |
| PYRUVATE METABOLISM%KEGG%HSA00620 | -1.67 | 0.009107468 | 0.0486657 |
| REGULATION OF STEROL TRANSPORT%GOBP%GO:0032371 | -1.67 | 0.009160305 | 0.04873571 |

## Slide 14
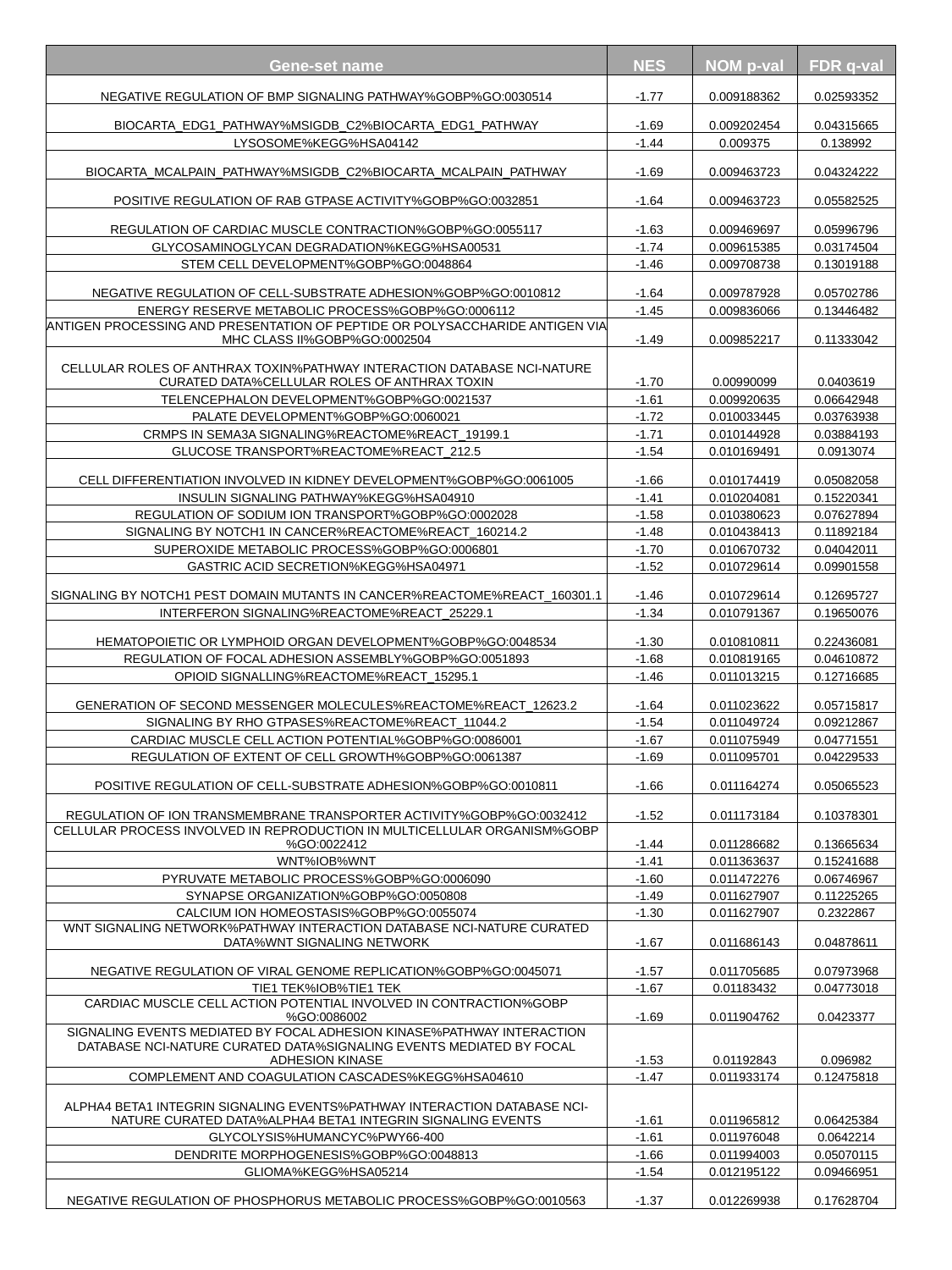

| Gene-set name | NES | NOM p-val | FDR q-val |
| --- | --- | --- | --- |
| NEGATIVE REGULATION OF BMP SIGNALING PATHWAY%GOBP%GO:0030514 | -1.77 | 0.009188362 | 0.02593352 |
| BIOCARTA\_EDG1\_PATHWAY%MSIGDB\_C2%BIOCARTA\_EDG1\_PATHWAY | -1.69 | 0.009202454 | 0.04315665 |
| LYSOSOME%KEGG%HSA04142 | -1.44 | 0.009375 | 0.138992 |
| BIOCARTA\_MCALPAIN\_PATHWAY%MSIGDB\_C2%BIOCARTA\_MCALPAIN\_PATHWAY | -1.69 | 0.009463723 | 0.04324222 |
| POSITIVE REGULATION OF RAB GTPASE ACTIVITY%GOBP%GO:0032851 | -1.64 | 0.009463723 | 0.05582525 |
| REGULATION OF CARDIAC MUSCLE CONTRACTION%GOBP%GO:0055117 | -1.63 | 0.009469697 | 0.05996796 |
| GLYCOSAMINOGLYCAN DEGRADATION%KEGG%HSA00531 | -1.74 | 0.009615385 | 0.03174504 |
| STEM CELL DEVELOPMENT%GOBP%GO:0048864 | -1.46 | 0.009708738 | 0.13019188 |
| NEGATIVE REGULATION OF CELL-SUBSTRATE ADHESION%GOBP%GO:0010812 | -1.64 | 0.009787928 | 0.05702786 |
| ENERGY RESERVE METABOLIC PROCESS%GOBP%GO:0006112 | -1.45 | 0.009836066 | 0.13446482 |
| ANTIGEN PROCESSING AND PRESENTATION OF PEPTIDE OR POLYSACCHARIDE ANTIGEN VIA MHC CLASS II%GOBP%GO:0002504 | -1.49 | 0.009852217 | 0.11333042 |
| CELLULAR ROLES OF ANTHRAX TOXIN%PATHWAY INTERACTION DATABASE NCI-NATURE CURATED DATA%CELLULAR ROLES OF ANTHRAX TOXIN | -1.70 | 0.00990099 | 0.0403619 |
| TELENCEPHALON DEVELOPMENT%GOBP%GO:0021537 | -1.61 | 0.009920635 | 0.06642948 |
| PALATE DEVELOPMENT%GOBP%GO:0060021 | -1.72 | 0.010033445 | 0.03763938 |
| CRMPS IN SEMA3A SIGNALING%REACTOME%REACT\_19199.1 | -1.71 | 0.010144928 | 0.03884193 |
| GLUCOSE TRANSPORT%REACTOME%REACT\_212.5 | -1.54 | 0.010169491 | 0.0913074 |
| CELL DIFFERENTIATION INVOLVED IN KIDNEY DEVELOPMENT%GOBP%GO:0061005 | -1.66 | 0.010174419 | 0.05082058 |
| INSULIN SIGNALING PATHWAY%KEGG%HSA04910 | -1.41 | 0.010204081 | 0.15220341 |
| REGULATION OF SODIUM ION TRANSPORT%GOBP%GO:0002028 | -1.58 | 0.010380623 | 0.07627894 |
| SIGNALING BY NOTCH1 IN CANCER%REACTOME%REACT\_160214.2 | -1.48 | 0.010438413 | 0.11892184 |
| SUPEROXIDE METABOLIC PROCESS%GOBP%GO:0006801 | -1.70 | 0.010670732 | 0.04042011 |
| GASTRIC ACID SECRETION%KEGG%HSA04971 | -1.52 | 0.010729614 | 0.09901558 |
| SIGNALING BY NOTCH1 PEST DOMAIN MUTANTS IN CANCER%REACTOME%REACT\_160301.1 | -1.46 | 0.010729614 | 0.12695727 |
| INTERFERON SIGNALING%REACTOME%REACT\_25229.1 | -1.34 | 0.010791367 | 0.19650076 |
| HEMATOPOIETIC OR LYMPHOID ORGAN DEVELOPMENT%GOBP%GO:0048534 | -1.30 | 0.010810811 | 0.22436081 |
| REGULATION OF FOCAL ADHESION ASSEMBLY%GOBP%GO:0051893 | -1.68 | 0.010819165 | 0.04610872 |
| OPIOID SIGNALLING%REACTOME%REACT\_15295.1 | -1.46 | 0.011013215 | 0.12716685 |
| GENERATION OF SECOND MESSENGER MOLECULES%REACTOME%REACT\_12623.2 | -1.64 | 0.011023622 | 0.05715817 |
| SIGNALING BY RHO GTPASES%REACTOME%REACT\_11044.2 | -1.54 | 0.011049724 | 0.09212867 |
| CARDIAC MUSCLE CELL ACTION POTENTIAL%GOBP%GO:0086001 | -1.67 | 0.011075949 | 0.04771551 |
| REGULATION OF EXTENT OF CELL GROWTH%GOBP%GO:0061387 | -1.69 | 0.011095701 | 0.04229533 |
| POSITIVE REGULATION OF CELL-SUBSTRATE ADHESION%GOBP%GO:0010811 | -1.66 | 0.011164274 | 0.05065523 |
| REGULATION OF ION TRANSMEMBRANE TRANSPORTER ACTIVITY%GOBP%GO:0032412 | -1.52 | 0.011173184 | 0.10378301 |
| CELLULAR PROCESS INVOLVED IN REPRODUCTION IN MULTICELLULAR ORGANISM%GOBP%GO:0022412 | -1.44 | 0.011286682 | 0.13665634 |
| WNT%IOB%WNT | -1.41 | 0.011363637 | 0.15241688 |
| PYRUVATE METABOLIC PROCESS%GOBP%GO:0006090 | -1.60 | 0.011472276 | 0.06746967 |
| SYNAPSE ORGANIZATION%GOBP%GO:0050808 | -1.49 | 0.011627907 | 0.11225265 |
| CALCIUM ION HOMEOSTASIS%GOBP%GO:0055074 | -1.30 | 0.011627907 | 0.2322867 |
| WNT SIGNALING NETWORK%PATHWAY INTERACTION DATABASE NCI-NATURE CURATED DATA%WNT SIGNALING NETWORK | -1.67 | 0.011686143 | 0.04878611 |
| NEGATIVE REGULATION OF VIRAL GENOME REPLICATION%GOBP%GO:0045071 | -1.57 | 0.011705685 | 0.07973968 |
| TIE1 TEK%IOB%TIE1 TEK | -1.67 | 0.01183432 | 0.04773018 |
| CARDIAC MUSCLE CELL ACTION POTENTIAL INVOLVED IN CONTRACTION%GOBP%GO:0086002 | -1.69 | 0.011904762 | 0.0423377 |
| SIGNALING EVENTS MEDIATED BY FOCAL ADHESION KINASE%PATHWAY INTERACTION DATABASE NCI-NATURE CURATED DATA%SIGNALING EVENTS MEDIATED BY FOCAL ADHESION KINASE | -1.53 | 0.01192843 | 0.096982 |
| COMPLEMENT AND COAGULATION CASCADES%KEGG%HSA04610 | -1.47 | 0.011933174 | 0.12475818 |
| ALPHA4 BETA1 INTEGRIN SIGNALING EVENTS%PATHWAY INTERACTION DATABASE NCI-NATURE CURATED DATA%ALPHA4 BETA1 INTEGRIN SIGNALING EVENTS | -1.61 | 0.011965812 | 0.06425384 |
| GLYCOLYSIS%HUMANCYC%PWY66-400 | -1.61 | 0.011976048 | 0.0642214 |
| DENDRITE MORPHOGENESIS%GOBP%GO:0048813 | -1.66 | 0.011994003 | 0.05070115 |
| GLIOMA%KEGG%HSA05214 | -1.54 | 0.012195122 | 0.09466951 |
| NEGATIVE REGULATION OF PHOSPHORUS METABOLIC PROCESS%GOBP%GO:0010563 | -1.37 | 0.012269938 | 0.17628704 |

## Slide 15
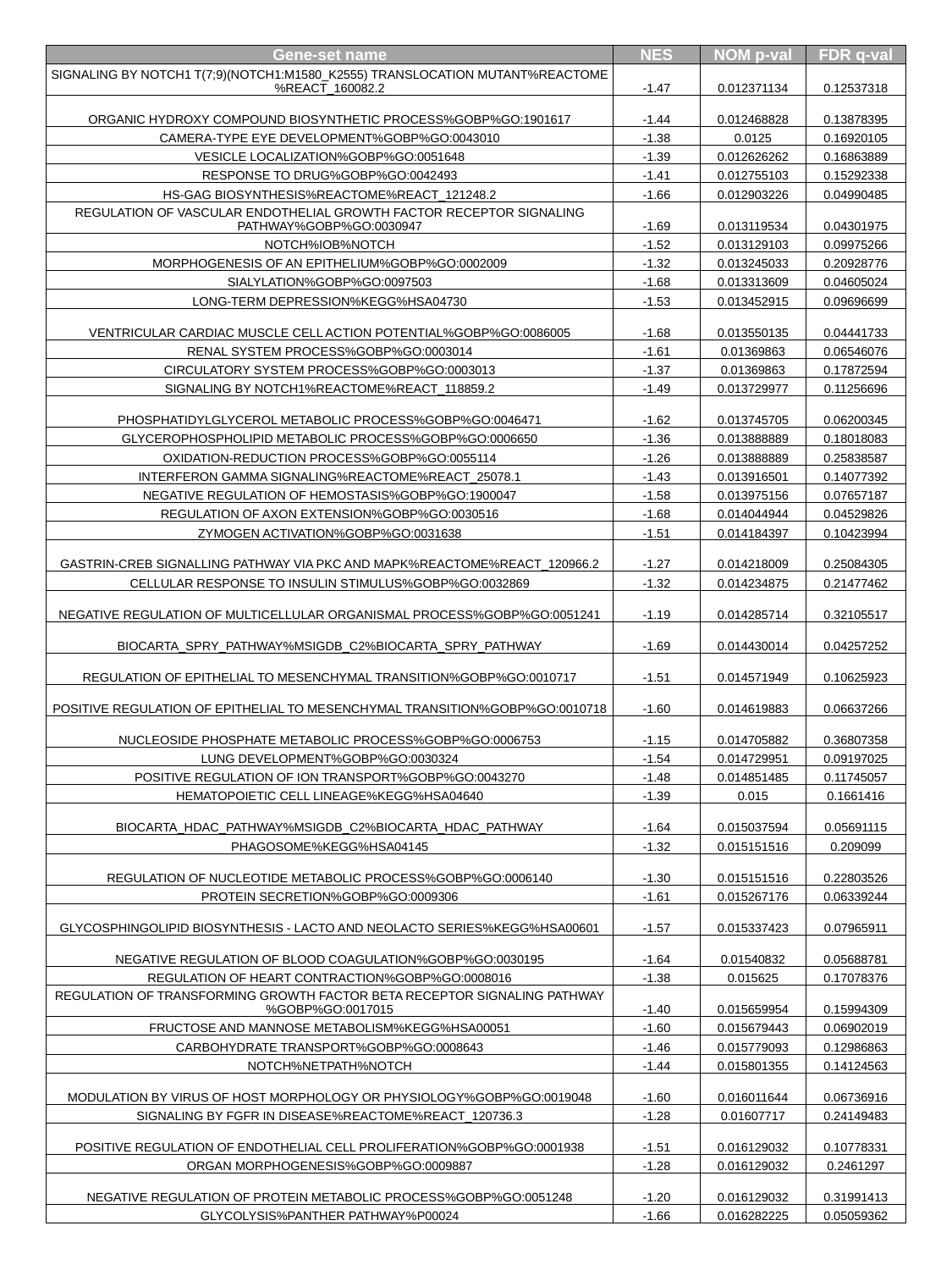

| Gene-set name | NES | NOM p-val | FDR q-val |
| --- | --- | --- | --- |
| SIGNALING BY NOTCH1 T(7;9)(NOTCH1:M1580\_K2555) TRANSLOCATION MUTANT%REACTOME%REACT\_160082.2 | -1.47 | 0.012371134 | 0.12537318 |
| ORGANIC HYDROXY COMPOUND BIOSYNTHETIC PROCESS%GOBP%GO:1901617 | -1.44 | 0.012468828 | 0.13878395 |
| CAMERA-TYPE EYE DEVELOPMENT%GOBP%GO:0043010 | -1.38 | 0.0125 | 0.16920105 |
| VESICLE LOCALIZATION%GOBP%GO:0051648 | -1.39 | 0.012626262 | 0.16863889 |
| RESPONSE TO DRUG%GOBP%GO:0042493 | -1.41 | 0.012755103 | 0.15292338 |
| HS-GAG BIOSYNTHESIS%REACTOME%REACT\_121248.2 | -1.66 | 0.012903226 | 0.04990485 |
| REGULATION OF VASCULAR ENDOTHELIAL GROWTH FACTOR RECEPTOR SIGNALING PATHWAY%GOBP%GO:0030947 | -1.69 | 0.013119534 | 0.04301975 |
| NOTCH%IOB%NOTCH | -1.52 | 0.013129103 | 0.09975266 |
| MORPHOGENESIS OF AN EPITHELIUM%GOBP%GO:0002009 | -1.32 | 0.013245033 | 0.20928776 |
| SIALYLATION%GOBP%GO:0097503 | -1.68 | 0.013313609 | 0.04605024 |
| LONG-TERM DEPRESSION%KEGG%HSA04730 | -1.53 | 0.013452915 | 0.09696699 |
| VENTRICULAR CARDIAC MUSCLE CELL ACTION POTENTIAL%GOBP%GO:0086005 | -1.68 | 0.013550135 | 0.04441733 |
| RENAL SYSTEM PROCESS%GOBP%GO:0003014 | -1.61 | 0.01369863 | 0.06546076 |
| CIRCULATORY SYSTEM PROCESS%GOBP%GO:0003013 | -1.37 | 0.01369863 | 0.17872594 |
| SIGNALING BY NOTCH1%REACTOME%REACT\_118859.2 | -1.49 | 0.013729977 | 0.11256696 |
| PHOSPHATIDYLGLYCEROL METABOLIC PROCESS%GOBP%GO:0046471 | -1.62 | 0.013745705 | 0.06200345 |
| GLYCEROPHOSPHOLIPID METABOLIC PROCESS%GOBP%GO:0006650 | -1.36 | 0.013888889 | 0.18018083 |
| OXIDATION-REDUCTION PROCESS%GOBP%GO:0055114 | -1.26 | 0.013888889 | 0.25838587 |
| INTERFERON GAMMA SIGNALING%REACTOME%REACT\_25078.1 | -1.43 | 0.013916501 | 0.14077392 |
| NEGATIVE REGULATION OF HEMOSTASIS%GOBP%GO:1900047 | -1.58 | 0.013975156 | 0.07657187 |
| REGULATION OF AXON EXTENSION%GOBP%GO:0030516 | -1.68 | 0.014044944 | 0.04529826 |
| ZYMOGEN ACTIVATION%GOBP%GO:0031638 | -1.51 | 0.014184397 | 0.10423994 |
| GASTRIN-CREB SIGNALLING PATHWAY VIA PKC AND MAPK%REACTOME%REACT\_120966.2 | -1.27 | 0.014218009 | 0.25084305 |
| CELLULAR RESPONSE TO INSULIN STIMULUS%GOBP%GO:0032869 | -1.32 | 0.014234875 | 0.21477462 |
| NEGATIVE REGULATION OF MULTICELLULAR ORGANISMAL PROCESS%GOBP%GO:0051241 | -1.19 | 0.014285714 | 0.32105517 |
| BIOCARTA\_SPRY\_PATHWAY%MSIGDB\_C2%BIOCARTA\_SPRY\_PATHWAY | -1.69 | 0.014430014 | 0.04257252 |
| REGULATION OF EPITHELIAL TO MESENCHYMAL TRANSITION%GOBP%GO:0010717 | -1.51 | 0.014571949 | 0.10625923 |
| POSITIVE REGULATION OF EPITHELIAL TO MESENCHYMAL TRANSITION%GOBP%GO:0010718 | -1.60 | 0.014619883 | 0.06637266 |
| NUCLEOSIDE PHOSPHATE METABOLIC PROCESS%GOBP%GO:0006753 | -1.15 | 0.014705882 | 0.36807358 |
| LUNG DEVELOPMENT%GOBP%GO:0030324 | -1.54 | 0.014729951 | 0.09197025 |
| POSITIVE REGULATION OF ION TRANSPORT%GOBP%GO:0043270 | -1.48 | 0.014851485 | 0.11745057 |
| HEMATOPOIETIC CELL LINEAGE%KEGG%HSA04640 | -1.39 | 0.015 | 0.1661416 |
| BIOCARTA\_HDAC\_PATHWAY%MSIGDB\_C2%BIOCARTA\_HDAC\_PATHWAY | -1.64 | 0.015037594 | 0.05691115 |
| PHAGOSOME%KEGG%HSA04145 | -1.32 | 0.015151516 | 0.209099 |
| REGULATION OF NUCLEOTIDE METABOLIC PROCESS%GOBP%GO:0006140 | -1.30 | 0.015151516 | 0.22803526 |
| PROTEIN SECRETION%GOBP%GO:0009306 | -1.61 | 0.015267176 | 0.06339244 |
| GLYCOSPHINGOLIPID BIOSYNTHESIS - LACTO AND NEOLACTO SERIES%KEGG%HSA00601 | -1.57 | 0.015337423 | 0.07965911 |
| NEGATIVE REGULATION OF BLOOD COAGULATION%GOBP%GO:0030195 | -1.64 | 0.01540832 | 0.05688781 |
| REGULATION OF HEART CONTRACTION%GOBP%GO:0008016 | -1.38 | 0.015625 | 0.17078376 |
| REGULATION OF TRANSFORMING GROWTH FACTOR BETA RECEPTOR SIGNALING PATHWAY%GOBP%GO:0017015 | -1.40 | 0.015659954 | 0.15994309 |
| FRUCTOSE AND MANNOSE METABOLISM%KEGG%HSA00051 | -1.60 | 0.015679443 | 0.06902019 |
| CARBOHYDRATE TRANSPORT%GOBP%GO:0008643 | -1.46 | 0.015779093 | 0.12986863 |
| NOTCH%NETPATH%NOTCH | -1.44 | 0.015801355 | 0.14124563 |
| MODULATION BY VIRUS OF HOST MORPHOLOGY OR PHYSIOLOGY%GOBP%GO:0019048 | -1.60 | 0.016011644 | 0.06736916 |
| SIGNALING BY FGFR IN DISEASE%REACTOME%REACT\_120736.3 | -1.28 | 0.01607717 | 0.24149483 |
| POSITIVE REGULATION OF ENDOTHELIAL CELL PROLIFERATION%GOBP%GO:0001938 | -1.51 | 0.016129032 | 0.10778331 |
| ORGAN MORPHOGENESIS%GOBP%GO:0009887 | -1.28 | 0.016129032 | 0.2461297 |
| NEGATIVE REGULATION OF PROTEIN METABOLIC PROCESS%GOBP%GO:0051248 | -1.20 | 0.016129032 | 0.31991413 |
| GLYCOLYSIS%PANTHER PATHWAY%P00024 | -1.66 | 0.016282225 | 0.05059362 |

## Slide 16
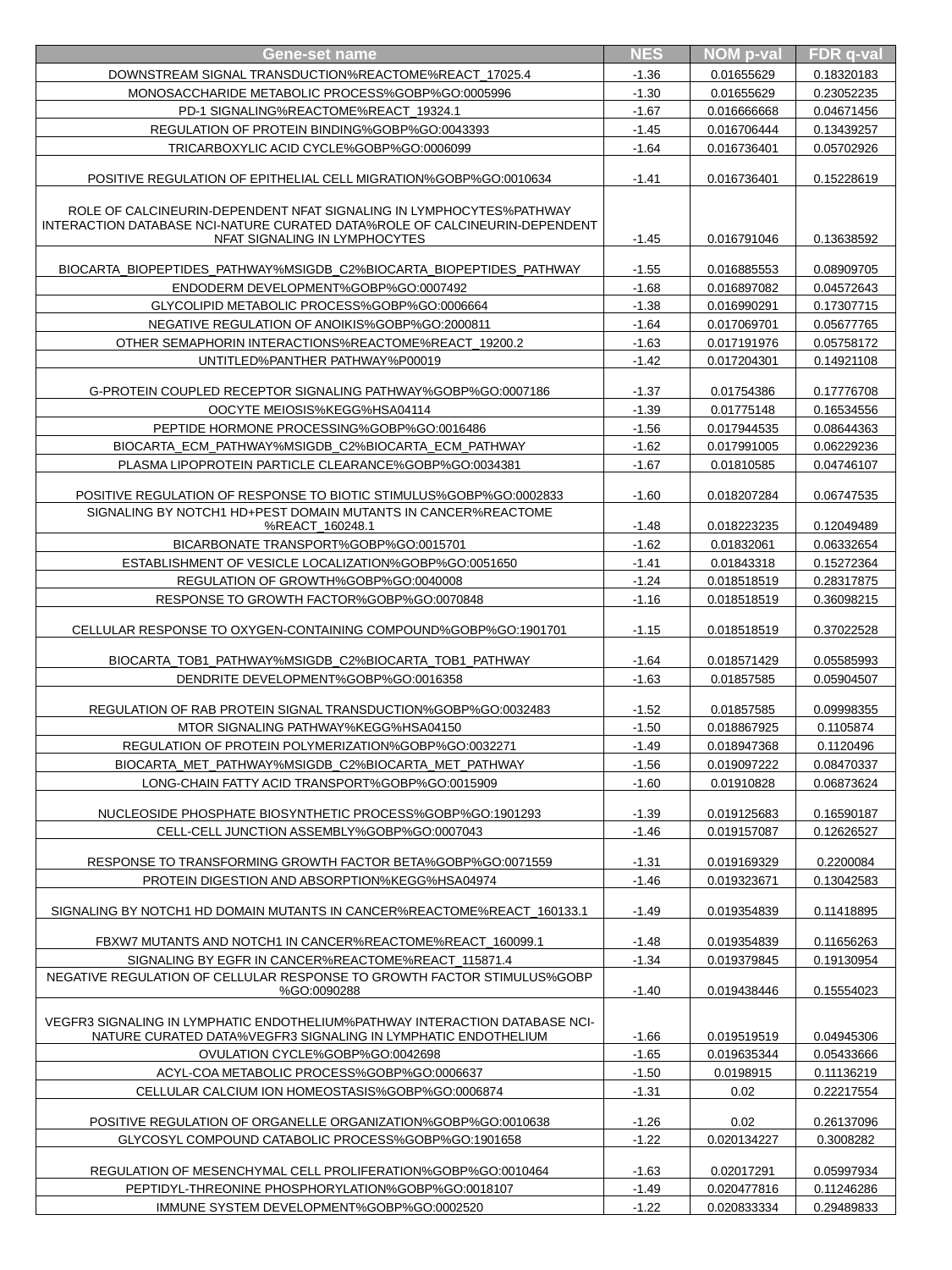

| Gene-set name | NES | NOM p-val | FDR q-val |
| --- | --- | --- | --- |
| DOWNSTREAM SIGNAL TRANSDUCTION%REACTOME%REACT\_17025.4 | -1.36 | 0.01655629 | 0.18320183 |
| MONOSACCHARIDE METABOLIC PROCESS%GOBP%GO:0005996 | -1.30 | 0.01655629 | 0.23052235 |
| PD-1 SIGNALING%REACTOME%REACT\_19324.1 | -1.67 | 0.016666668 | 0.04671456 |
| REGULATION OF PROTEIN BINDING%GOBP%GO:0043393 | -1.45 | 0.016706444 | 0.13439257 |
| TRICARBOXYLIC ACID CYCLE%GOBP%GO:0006099 | -1.64 | 0.016736401 | 0.05702926 |
| POSITIVE REGULATION OF EPITHELIAL CELL MIGRATION%GOBP%GO:0010634 | -1.41 | 0.016736401 | 0.15228619 |
| ROLE OF CALCINEURIN-DEPENDENT NFAT SIGNALING IN LYMPHOCYTES%PATHWAY INTERACTION DATABASE NCI-NATURE CURATED DATA%ROLE OF CALCINEURIN-DEPENDENT NFAT SIGNALING IN LYMPHOCYTES | -1.45 | 0.016791046 | 0.13638592 |
| BIOCARTA\_BIOPEPTIDES\_PATHWAY%MSIGDB\_C2%BIOCARTA\_BIOPEPTIDES\_PATHWAY | -1.55 | 0.016885553 | 0.08909705 |
| ENDODERM DEVELOPMENT%GOBP%GO:0007492 | -1.68 | 0.016897082 | 0.04572643 |
| GLYCOLIPID METABOLIC PROCESS%GOBP%GO:0006664 | -1.38 | 0.016990291 | 0.17307715 |
| NEGATIVE REGULATION OF ANOIKIS%GOBP%GO:2000811 | -1.64 | 0.017069701 | 0.05677765 |
| OTHER SEMAPHORIN INTERACTIONS%REACTOME%REACT\_19200.2 | -1.63 | 0.017191976 | 0.05758172 |
| UNTITLED%PANTHER PATHWAY%P00019 | -1.42 | 0.017204301 | 0.14921108 |
| G-PROTEIN COUPLED RECEPTOR SIGNALING PATHWAY%GOBP%GO:0007186 | -1.37 | 0.01754386 | 0.17776708 |
| OOCYTE MEIOSIS%KEGG%HSA04114 | -1.39 | 0.01775148 | 0.16534556 |
| PEPTIDE HORMONE PROCESSING%GOBP%GO:0016486 | -1.56 | 0.017944535 | 0.08644363 |
| BIOCARTA\_ECM\_PATHWAY%MSIGDB\_C2%BIOCARTA\_ECM\_PATHWAY | -1.62 | 0.017991005 | 0.06229236 |
| PLASMA LIPOPROTEIN PARTICLE CLEARANCE%GOBP%GO:0034381 | -1.67 | 0.01810585 | 0.04746107 |
| POSITIVE REGULATION OF RESPONSE TO BIOTIC STIMULUS%GOBP%GO:0002833 | -1.60 | 0.018207284 | 0.06747535 |
| SIGNALING BY NOTCH1 HD+PEST DOMAIN MUTANTS IN CANCER%REACTOME%REACT\_160248.1 | -1.48 | 0.018223235 | 0.12049489 |
| BICARBONATE TRANSPORT%GOBP%GO:0015701 | -1.62 | 0.01832061 | 0.06332654 |
| ESTABLISHMENT OF VESICLE LOCALIZATION%GOBP%GO:0051650 | -1.41 | 0.01843318 | 0.15272364 |
| REGULATION OF GROWTH%GOBP%GO:0040008 | -1.24 | 0.018518519 | 0.28317875 |
| RESPONSE TO GROWTH FACTOR%GOBP%GO:0070848 | -1.16 | 0.018518519 | 0.36098215 |
| CELLULAR RESPONSE TO OXYGEN-CONTAINING COMPOUND%GOBP%GO:1901701 | -1.15 | 0.018518519 | 0.37022528 |
| BIOCARTA\_TOB1\_PATHWAY%MSIGDB\_C2%BIOCARTA\_TOB1\_PATHWAY | -1.64 | 0.018571429 | 0.05585993 |
| DENDRITE DEVELOPMENT%GOBP%GO:0016358 | -1.63 | 0.01857585 | 0.05904507 |
| REGULATION OF RAB PROTEIN SIGNAL TRANSDUCTION%GOBP%GO:0032483 | -1.52 | 0.01857585 | 0.09998355 |
| MTOR SIGNALING PATHWAY%KEGG%HSA04150 | -1.50 | 0.018867925 | 0.1105874 |
| REGULATION OF PROTEIN POLYMERIZATION%GOBP%GO:0032271 | -1.49 | 0.018947368 | 0.1120496 |
| BIOCARTA\_MET\_PATHWAY%MSIGDB\_C2%BIOCARTA\_MET\_PATHWAY | -1.56 | 0.019097222 | 0.08470337 |
| LONG-CHAIN FATTY ACID TRANSPORT%GOBP%GO:0015909 | -1.60 | 0.01910828 | 0.06873624 |
| NUCLEOSIDE PHOSPHATE BIOSYNTHETIC PROCESS%GOBP%GO:1901293 | -1.39 | 0.019125683 | 0.16590187 |
| CELL-CELL JUNCTION ASSEMBLY%GOBP%GO:0007043 | -1.46 | 0.019157087 | 0.12626527 |
| RESPONSE TO TRANSFORMING GROWTH FACTOR BETA%GOBP%GO:0071559 | -1.31 | 0.019169329 | 0.2200084 |
| PROTEIN DIGESTION AND ABSORPTION%KEGG%HSA04974 | -1.46 | 0.019323671 | 0.13042583 |
| SIGNALING BY NOTCH1 HD DOMAIN MUTANTS IN CANCER%REACTOME%REACT\_160133.1 | -1.49 | 0.019354839 | 0.11418895 |
| FBXW7 MUTANTS AND NOTCH1 IN CANCER%REACTOME%REACT\_160099.1 | -1.48 | 0.019354839 | 0.11656263 |
| SIGNALING BY EGFR IN CANCER%REACTOME%REACT\_115871.4 | -1.34 | 0.019379845 | 0.19130954 |
| NEGATIVE REGULATION OF CELLULAR RESPONSE TO GROWTH FACTOR STIMULUS%GOBP%GO:0090288 | -1.40 | 0.019438446 | 0.15554023 |
| VEGFR3 SIGNALING IN LYMPHATIC ENDOTHELIUM%PATHWAY INTERACTION DATABASE NCI-NATURE CURATED DATA%VEGFR3 SIGNALING IN LYMPHATIC ENDOTHELIUM | -1.66 | 0.019519519 | 0.04945306 |
| OVULATION CYCLE%GOBP%GO:0042698 | -1.65 | 0.019635344 | 0.05433666 |
| ACYL-COA METABOLIC PROCESS%GOBP%GO:0006637 | -1.50 | 0.0198915 | 0.11136219 |
| CELLULAR CALCIUM ION HOMEOSTASIS%GOBP%GO:0006874 | -1.31 | 0.02 | 0.22217554 |
| POSITIVE REGULATION OF ORGANELLE ORGANIZATION%GOBP%GO:0010638 | -1.26 | 0.02 | 0.26137096 |
| GLYCOSYL COMPOUND CATABOLIC PROCESS%GOBP%GO:1901658 | -1.22 | 0.020134227 | 0.3008282 |
| REGULATION OF MESENCHYMAL CELL PROLIFERATION%GOBP%GO:0010464 | -1.63 | 0.02017291 | 0.05997934 |
| PEPTIDYL-THREONINE PHOSPHORYLATION%GOBP%GO:0018107 | -1.49 | 0.020477816 | 0.11246286 |
| IMMUNE SYSTEM DEVELOPMENT%GOBP%GO:0002520 | -1.22 | 0.020833334 | 0.29489833 |

## Slide 17
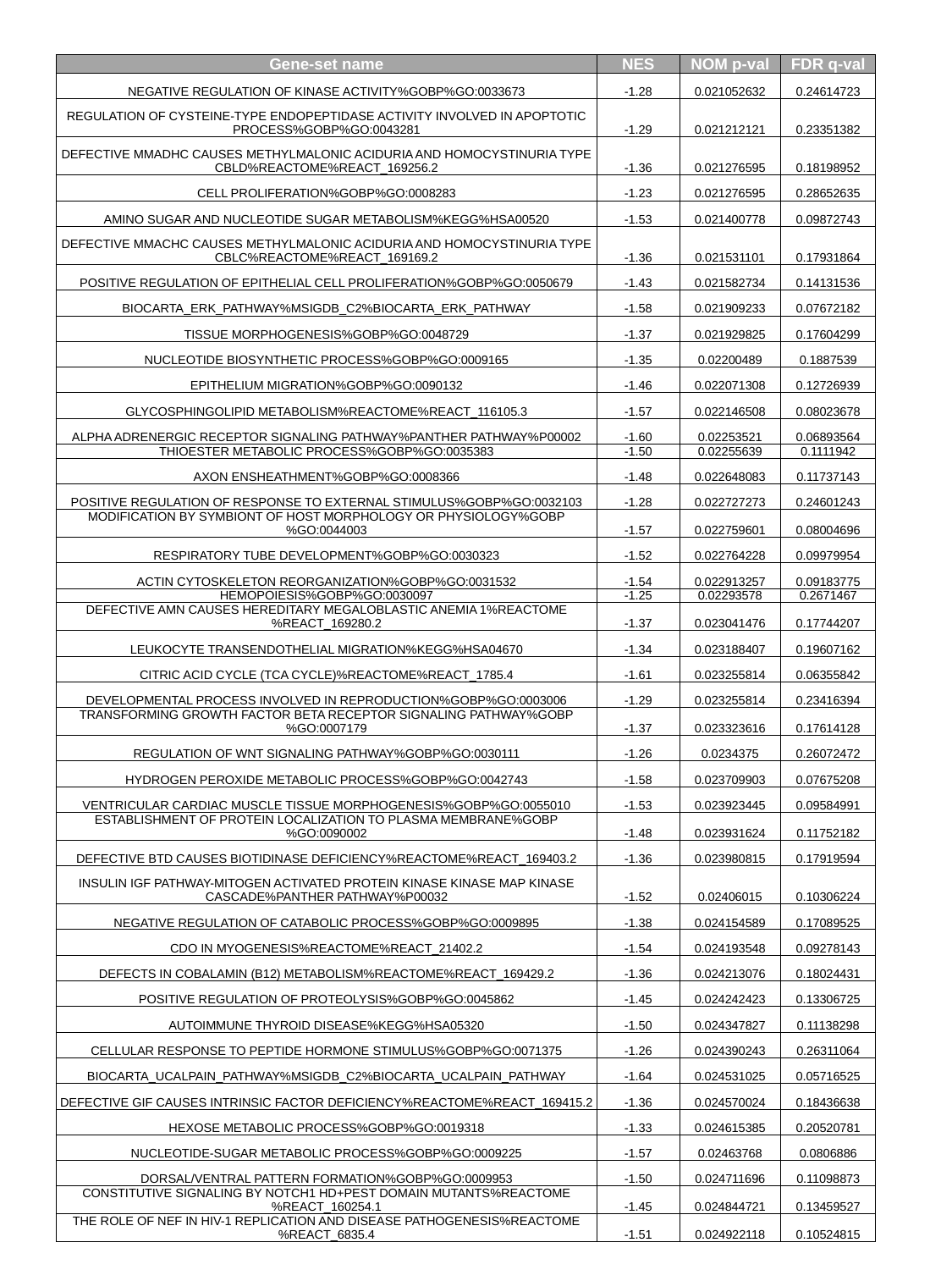

| Gene-set name | NES | NOM p-val | FDR q-val |
| --- | --- | --- | --- |
| NEGATIVE REGULATION OF KINASE ACTIVITY%GOBP%GO:0033673 | -1.28 | 0.021052632 | 0.24614723 |
| REGULATION OF CYSTEINE-TYPE ENDOPEPTIDASE ACTIVITY INVOLVED IN APOPTOTIC PROCESS%GOBP%GO:0043281 | -1.29 | 0.021212121 | 0.23351382 |
| DEFECTIVE MMADHC CAUSES METHYLMALONIC ACIDURIA AND HOMOCYSTINURIA TYPE CBLD%REACTOME%REACT\_169256.2 | -1.36 | 0.021276595 | 0.18198952 |
| CELL PROLIFERATION%GOBP%GO:0008283 | -1.23 | 0.021276595 | 0.28652635 |
| AMINO SUGAR AND NUCLEOTIDE SUGAR METABOLISM%KEGG%HSA00520 | -1.53 | 0.021400778 | 0.09872743 |
| DEFECTIVE MMACHC CAUSES METHYLMALONIC ACIDURIA AND HOMOCYSTINURIA TYPE CBLC%REACTOME%REACT\_169169.2 | -1.36 | 0.021531101 | 0.17931864 |
| POSITIVE REGULATION OF EPITHELIAL CELL PROLIFERATION%GOBP%GO:0050679 | -1.43 | 0.021582734 | 0.14131536 |
| BIOCARTA\_ERK\_PATHWAY%MSIGDB\_C2%BIOCARTA\_ERK\_PATHWAY | -1.58 | 0.021909233 | 0.07672182 |
| TISSUE MORPHOGENESIS%GOBP%GO:0048729 | -1.37 | 0.021929825 | 0.17604299 |
| NUCLEOTIDE BIOSYNTHETIC PROCESS%GOBP%GO:0009165 | -1.35 | 0.02200489 | 0.1887539 |
| EPITHELIUM MIGRATION%GOBP%GO:0090132 | -1.46 | 0.022071308 | 0.12726939 |
| GLYCOSPHINGOLIPID METABOLISM%REACTOME%REACT\_116105.3 | -1.57 | 0.022146508 | 0.08023678 |
| ALPHA ADRENERGIC RECEPTOR SIGNALING PATHWAY%PANTHER PATHWAY%P00002 | -1.60 | 0.02253521 | 0.06893564 |
| THIOESTER METABOLIC PROCESS%GOBP%GO:0035383 | -1.50 | 0.02255639 | 0.1111942 |
| AXON ENSHEATHMENT%GOBP%GO:0008366 | -1.48 | 0.022648083 | 0.11737143 |
| POSITIVE REGULATION OF RESPONSE TO EXTERNAL STIMULUS%GOBP%GO:0032103 | -1.28 | 0.022727273 | 0.24601243 |
| MODIFICATION BY SYMBIONT OF HOST MORPHOLOGY OR PHYSIOLOGY%GOBP%GO:0044003 | -1.57 | 0.022759601 | 0.08004696 |
| RESPIRATORY TUBE DEVELOPMENT%GOBP%GO:0030323 | -1.52 | 0.022764228 | 0.09979954 |
| ACTIN CYTOSKELETON REORGANIZATION%GOBP%GO:0031532 | -1.54 | 0.022913257 | 0.09183775 |
| HEMOPOIESIS%GOBP%GO:0030097 | -1.25 | 0.02293578 | 0.2671467 |
| DEFECTIVE AMN CAUSES HEREDITARY MEGALOBLASTIC ANEMIA 1%REACTOME%REACT\_169280.2 | -1.37 | 0.023041476 | 0.17744207 |
| LEUKOCYTE TRANSENDOTHELIAL MIGRATION%KEGG%HSA04670 | -1.34 | 0.023188407 | 0.19607162 |
| CITRIC ACID CYCLE (TCA CYCLE)%REACTOME%REACT\_1785.4 | -1.61 | 0.023255814 | 0.06355842 |
| DEVELOPMENTAL PROCESS INVOLVED IN REPRODUCTION%GOBP%GO:0003006 | -1.29 | 0.023255814 | 0.23416394 |
| TRANSFORMING GROWTH FACTOR BETA RECEPTOR SIGNALING PATHWAY%GOBP%GO:0007179 | -1.37 | 0.023323616 | 0.17614128 |
| REGULATION OF WNT SIGNALING PATHWAY%GOBP%GO:0030111 | -1.26 | 0.0234375 | 0.26072472 |
| HYDROGEN PEROXIDE METABOLIC PROCESS%GOBP%GO:0042743 | -1.58 | 0.023709903 | 0.07675208 |
| VENTRICULAR CARDIAC MUSCLE TISSUE MORPHOGENESIS%GOBP%GO:0055010 | -1.53 | 0.023923445 | 0.09584991 |
| ESTABLISHMENT OF PROTEIN LOCALIZATION TO PLASMA MEMBRANE%GOBP%GO:0090002 | -1.48 | 0.023931624 | 0.11752182 |
| DEFECTIVE BTD CAUSES BIOTIDINASE DEFICIENCY%REACTOME%REACT\_169403.2 | -1.36 | 0.023980815 | 0.17919594 |
| INSULIN IGF PATHWAY-MITOGEN ACTIVATED PROTEIN KINASE KINASE MAP KINASE CASCADE%PANTHER PATHWAY%P00032 | -1.52 | 0.02406015 | 0.10306224 |
| NEGATIVE REGULATION OF CATABOLIC PROCESS%GOBP%GO:0009895 | -1.38 | 0.024154589 | 0.17089525 |
| CDO IN MYOGENESIS%REACTOME%REACT\_21402.2 | -1.54 | 0.024193548 | 0.09278143 |
| DEFECTS IN COBALAMIN (B12) METABOLISM%REACTOME%REACT\_169429.2 | -1.36 | 0.024213076 | 0.18024431 |
| POSITIVE REGULATION OF PROTEOLYSIS%GOBP%GO:0045862 | -1.45 | 0.024242423 | 0.13306725 |
| AUTOIMMUNE THYROID DISEASE%KEGG%HSA05320 | -1.50 | 0.024347827 | 0.11138298 |
| CELLULAR RESPONSE TO PEPTIDE HORMONE STIMULUS%GOBP%GO:0071375 | -1.26 | 0.024390243 | 0.26311064 |
| BIOCARTA\_UCALPAIN\_PATHWAY%MSIGDB\_C2%BIOCARTA\_UCALPAIN\_PATHWAY | -1.64 | 0.024531025 | 0.05716525 |
| DEFECTIVE GIF CAUSES INTRINSIC FACTOR DEFICIENCY%REACTOME%REACT\_169415.2 | -1.36 | 0.024570024 | 0.18436638 |
| HEXOSE METABOLIC PROCESS%GOBP%GO:0019318 | -1.33 | 0.024615385 | 0.20520781 |
| NUCLEOTIDE-SUGAR METABOLIC PROCESS%GOBP%GO:0009225 | -1.57 | 0.02463768 | 0.0806886 |
| DORSAL/VENTRAL PATTERN FORMATION%GOBP%GO:0009953 | -1.50 | 0.024711696 | 0.11098873 |
| CONSTITUTIVE SIGNALING BY NOTCH1 HD+PEST DOMAIN MUTANTS%REACTOME%REACT\_160254.1 | -1.45 | 0.024844721 | 0.13459527 |
| THE ROLE OF NEF IN HIV-1 REPLICATION AND DISEASE PATHOGENESIS%REACTOME%REACT\_6835.4 | -1.51 | 0.024922118 | 0.10524815 |
