# Supplementary material for: Antitumor activity of the novel multi-kinase inhibitor EC-70124 in triple negative breast cancer
Source: Oncotarget. 2015 Aug 12;6(29):27923–37. doi: 10.18632/oncotarget.4736 (PMC4695035; doi:10.18632/oncotarget.4736)
Supplement: Supplementary file 3 [file oncotarget-06-27923-s003.ppt]

## Slide 1
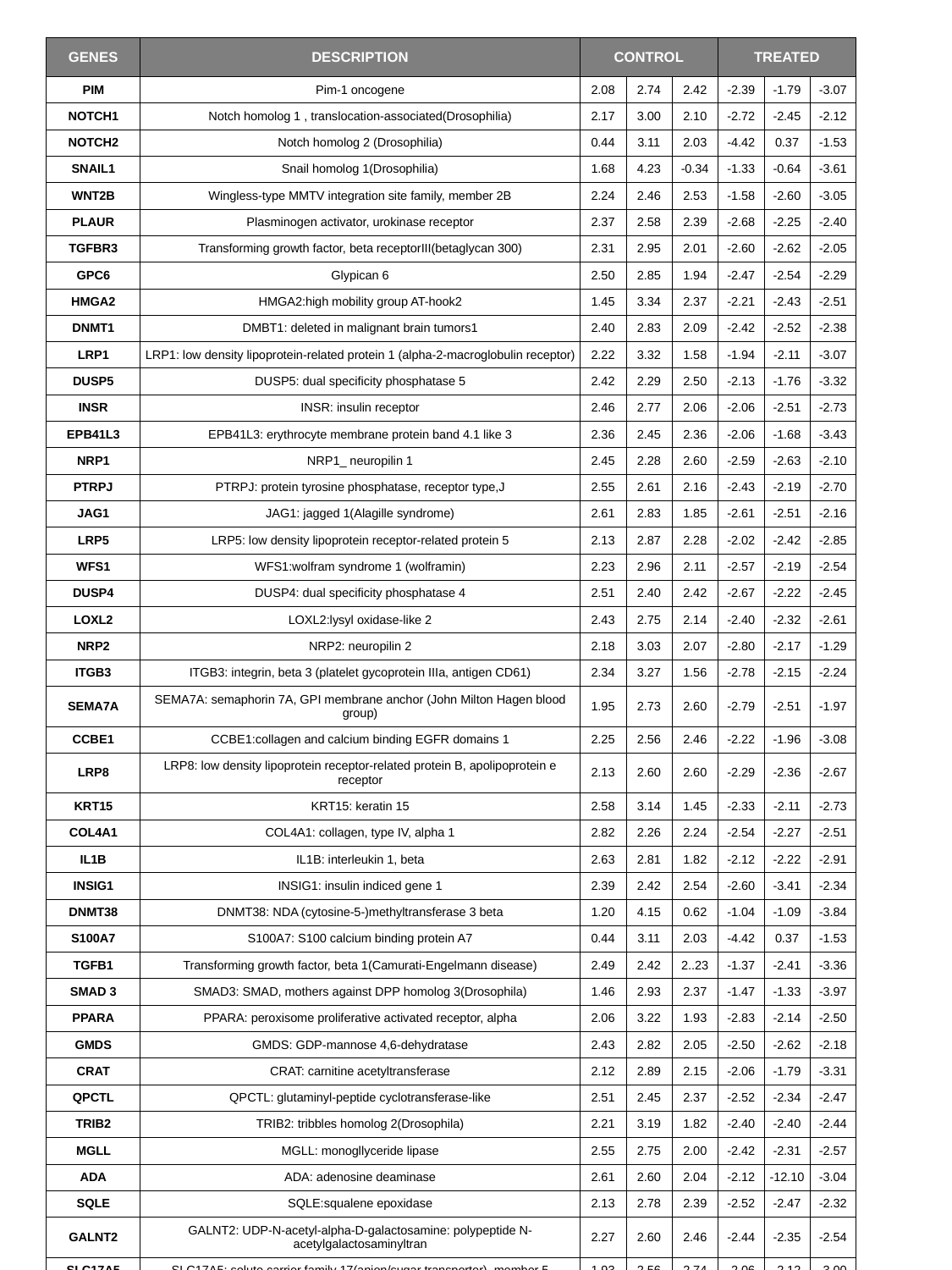

| GENES | DESCRIPTION | CONTROL | | | TREATED | | |
| --- | --- | --- | --- | --- | --- | --- | --- |
| PIM | Pim-1 oncogene | 2.08 | 2.74 | 2.42 | -2.39 | -1.79 | -3.07 |
| NOTCH1 | Notch homolog 1 , translocation-associated(Drosophilia) | 2.17 | 3.00 | 2.10 | -2.72 | -2.45 | -2.12 |
| NOTCH2 | Notch homolog 2 (Drosophilia) | 0.44 | 3.11 | 2.03 | -4.42 | 0.37 | -1.53 |
| SNAIL1 | Snail homolog 1(Drosophilia) | 1.68 | 4.23 | -0.34 | -1.33 | -0.64 | -3.61 |
| WNT2B | Wingless-type MMTV integration site family, member 2B | 2.24 | 2.46 | 2.53 | -1.58 | -2.60 | -3.05 |
| PLAUR | Plasminogen activator, urokinase receptor | 2.37 | 2.58 | 2.39 | -2.68 | -2.25 | -2.40 |
| TGFBR3 | Transforming growth factor, beta receptorIII(betaglycan 300) | 2.31 | 2.95 | 2.01 | -2.60 | -2.62 | -2.05 |
| GPC6 | Glypican 6 | 2.50 | 2.85 | 1.94 | -2.47 | -2.54 | -2.29 |
| HMGA2 | HMGA2:high mobility group AT-hook2 | 1.45 | 3.34 | 2.37 | -2.21 | -2.43 | -2.51 |
| DNMT1 | DMBT1: deleted in malignant brain tumors1 | 2.40 | 2.83 | 2.09 | -2.42 | -2.52 | -2.38 |
| LRP1 | LRP1: low density lipoprotein-related protein 1 (alpha-2-macroglobulin receptor) | 2.22 | 3.32 | 1.58 | -1.94 | -2.11 | -3.07 |
| DUSP5 | DUSP5: dual specificity phosphatase 5 | 2.42 | 2.29 | 2.50 | -2.13 | -1.76 | -3.32 |
| INSR | INSR: insulin receptor | 2.46 | 2.77 | 2.06 | -2.06 | -2.51 | -2.73 |
| EPB41L3 | EPB41L3: erythrocyte membrane protein band 4.1 like 3 | 2.36 | 2.45 | 2.36 | -2.06 | -1.68 | -3.43 |
| NRP1 | NRP1\_ neuropilin 1 | 2.45 | 2.28 | 2.60 | -2.59 | -2.63 | -2.10 |
| PTRPJ | PTRPJ: protein tyrosine phosphatase, receptor type,J | 2.55 | 2.61 | 2.16 | -2.43 | -2.19 | -2.70 |
| JAG1 | JAG1: jagged 1(Alagille syndrome) | 2.61 | 2.83 | 1.85 | -2.61 | -2.51 | -2.16 |
| LRP5 | LRP5: low density lipoprotein receptor-related protein 5 | 2.13 | 2.87 | 2.28 | -2.02 | -2.42 | -2.85 |
| WFS1 | WFS1:wolfram syndrome 1 (wolframin) | 2.23 | 2.96 | 2.11 | -2.57 | -2.19 | -2.54 |
| DUSP4 | DUSP4: dual specificity phosphatase 4 | 2.51 | 2.40 | 2.42 | -2.67 | -2.22 | -2.45 |
| LOXL2 | LOXL2:lysyl oxidase-like 2 | 2.43 | 2.75 | 2.14 | -2.40 | -2.32 | -2.61 |
| NRP2 | NRP2: neuropilin 2 | 2.18 | 3.03 | 2.07 | -2.80 | -2.17 | -1.29 |
| ITGB3 | ITGB3: integrin, beta 3 (platelet gycoprotein IIIa, antigen CD61) | 2.34 | 3.27 | 1.56 | -2.78 | -2.15 | -2.24 |
| SEMA7A | SEMA7A: semaphorin 7A, GPI membrane anchor (John Milton Hagen blood group) | 1.95 | 2.73 | 2.60 | -2.79 | -2.51 | -1.97 |
| CCBE1 | CCBE1:collagen and calcium binding EGFR domains 1 | 2.25 | 2.56 | 2.46 | -2.22 | -1.96 | -3.08 |
| LRP8 | LRP8: low density lipoprotein receptor-related protein B, apolipoprotein e receptor | 2.13 | 2.60 | 2.60 | -2.29 | -2.36 | -2.67 |
| KRT15 | KRT15: keratin 15 | 2.58 | 3.14 | 1.45 | -2.33 | -2.11 | -2.73 |
| COL4A1 | COL4A1: collagen, type IV, alpha 1 | 2.82 | 2.26 | 2.24 | -2.54 | -2.27 | -2.51 |
| IL1B | IL1B: interleukin 1, beta | 2.63 | 2.81 | 1.82 | -2.12 | -2.22 | -2.91 |
| INSIG1 | INSIG1: insulin indiced gene 1 | 2.39 | 2.42 | 2.54 | -2.60 | -3.41 | -2.34 |
| DNMT38 | DNMT38: NDA (cytosine-5-)methyltransferase 3 beta | 1.20 | 4.15 | 0.62 | -1.04 | -1.09 | -3.84 |
| S100A7 | S100A7: S100 calcium binding protein A7 | 0.44 | 3.11 | 2.03 | -4.42 | 0.37 | -1.53 |
| TGFB1 | Transforming growth factor, beta 1(Camurati-Engelmann disease) | 2.49 | 2.42 | 2..23 | -1.37 | -2.41 | -3.36 |
| SMAD 3 | SMAD3: SMAD, mothers against DPP homolog 3(Drosophila) | 1.46 | 2.93 | 2.37 | -1.47 | -1.33 | -3.97 |
| PPARA | PPARA: peroxisome proliferative activated receptor, alpha | 2.06 | 3.22 | 1.93 | -2.83 | -2.14 | -2.50 |
| GMDS | GMDS: GDP-mannose 4,6-dehydratase | 2.43 | 2.82 | 2.05 | -2.50 | -2.62 | -2.18 |
| CRAT | CRAT: carnitine acetyltransferase | 2.12 | 2.89 | 2.15 | -2.06 | -1.79 | -3.31 |
| QPCTL | QPCTL: glutaminyl-peptide cyclotransferase-like | 2.51 | 2.45 | 2.37 | -2.52 | -2.34 | -2.47 |
| TRIB2 | TRIB2: tribbles homolog 2(Drosophila) | 2.21 | 3.19 | 1.82 | -2.40 | -2.40 | -2.44 |
| MGLL | MGLL: monogllyceride lipase | 2.55 | 2.75 | 2.00 | -2.42 | -2.31 | -2.57 |
| ADA | ADA: adenosine deaminase | 2.61 | 2.60 | 2.04 | -2.12 | -12.10 | -3.04 |
| SQLE | SQLE:squalene epoxidase | 2.13 | 2.78 | 2.39 | -2.52 | -2.47 | -2.32 |
| GALNT2 | GALNT2: UDP-N-acetyl-alpha-D-galactosamine: polypeptide N-acetylgalactosaminyltran | 2.27 | 2.60 | 2.46 | -2.44 | -2.35 | -2.54 |
| SLC17A5 | SLC17A5: solute carrier family 17(anion/sugar transporter), member 5 | 1.93 | 2.56 | 2.74 | -2.06 | -2.12 | -3.00 |
| ST6GAL1 | ST6GAL1: ST6 beta-galactosamide alpha-2,6- slalytransferase 1 | 2.68 | 3.26 | 1.14 | -2.40 | -2.20 | -2.46 |
| ENPP1 | ENPP1: ectonucleotide pyrophosphatase/phosphodiesterase 1 | 2.32 | 2.40 | 2.59 | -2.48 | -2.14 | -2.70 |
| LIPG | LIPG: lipase, endothelial | 2.14 | 2.28 | 2.32 | -2.48 | -2.44 | -2.39 |
| CST2 | CST2: cystatin SA | 2.12 | 23.28 | 1.63 | -2.83 | -2.84 | -1.36 |
| KIAA1199 | KIAA1199: KIAA1199 | 2.56 | 2.90 | 1.79 | -2.37 | -2.18 | -2.71 |
| ADAMTSL1 | ADAMTSL1: ADAMTS-like 1 | 2.66 | 2.58 | 2.07 | -2.31 | -2.39 | -2.61 |
| KAL1 | KAL1: Kallmann syndrome 1 sequence | 2.18 | 2.78 | 2.33 | -2.75 | -2.32 | -2.22 |
| FN1 | FN1: fibronectin 1 | 2.40 | 2.81 | 2.10 | -2.41 | -2.40 | -2.49 |
| PRTRS | PRTRS: protein tyrosine phosphatase, receptor type, S | 2.05 | 2.76 | 2.47 | -2.33 | -2.30 | -2.67 |
| ITGA6 | ITGA6: integrin, alpha 6 | 2.27 | 2.44 | 2.60 | -2.68 | -2.17 | -2.47 |
| MMP14 | MMP14: matrix metallopeptidase 14(membrane-inserted) | 2.42 | 2.67 | 2.25 | -2.21 | -2.32 | -2.78 |
| ITGA10 | ITGA10: integrin, alpha 10 | 2.28 | 3.00 | 1.89 | -2.30 | -2.29 | -2.67 |
| PLXNA2 | PLXNA2: plexin A2 | 2.41 | 2.99 | 1.87 | -2.47 | -2.34 | -2.46 |
| COL4A2 | COL4A2: collagen. type IV, alpha 2 | 2.37 | 2.84 | 2.08 | -2.56 | -2.22 | -2.51 |
| ATP2B4 | ATP2B4: ATPase, Ca++ transporting, plasma membrane 4 | 2.33 | 2.66 | 2.27 | -2.22 | -1.96 | -3.09 |
| THBD | THBD: thrombomodulin | 2.93 | 2.54 | 1.80 | -2.52 | -2.39 | -2.35 |
| LOX | LOX: lysyl oxidase | 2.45 | 2.72 | 2.14 | 2.45 | -2.24 | -2.62 |
| LTBP1 | LTBP1: latent transforming growth factor beta binding protein 1 | 2.41 | 2.43 | 2.49 | -2.42 | -2.34 | -2.57 |
